# Supplementary material for: Novel 1,3,4-Thiadiazole Derivatives: Synthesis, Antiviral Bioassay and Regulation the Photosynthetic Pathway of Tobacco against TMV Infection
Source: Int J Mol Sci. 2023 May 17;24(10):8881. doi: 10.3390/ijms24108881 (PMC10219444; doi:10.3390/ijms24108881)
Supplement: Supplementary file 1 [file ijms-24-08881-s001.zip › ijms-2353468-supplementary.pdf]

Novel 1,3,4-Thiadiazole Derivatives: Synthesis, Antiviral Bioassay, and Regulation the Photosynthetic Pathway of Tobacco against TMV Infection

Huanlin Zheng [1], Fanglin Wen [1], Chengzhi Zhang [1], Rui Luo [2] and Zhibing Wu [1,*]

[1] State Key Laboratory Breeding Base of Green Pesticide and Agricultural Bioengineering, Key Laboratory of Green Pesticide and Agricultural Bioengineering, Ministry of Education, Center for R&D of Fine Chemicals of Guizhou University, Guiyang 550025, China
[2] College of Life Sciences, Guizhou University, Guiyang 550025, China
* Correspondence: zbwu@gzu.edu.cn

Supporting Information

1. Detailed Antiviral Biological Assay Methods 2-3

2. Detailed Inhibition Rate of EC_{50} Values 3-4

3. Crystallographic Data of Title Compound **E_3** 4-5

4. Protective and Curative Activity Images of Title Compounds 5-6

5. ^{1}H NMR, ^{13}C NMR and HRMS Data of Intermediates and Title Compounds 7-13

6. Copies of Intermediates and Title Compounds 13-64

1. Detailed Antiviral Biological Assay Methods

The antiviral biological assay was carried out in a growth chamber at a temperature of 25 °C. The synthesized compounds were first dissolved within a suitable amount of DMSO and diluted with water containing 0.1% TW-80 to make a concentration of 500 μg/mL, and the commercialized antiviral agent ningnanmycin was used as the positive control. Ningnanmycin is 8% aqueous solution (Huaqiang biological, Wuhan, China), which can be diluted directly with water to the corresponding concentration.

1.1 Inactivation Effect of Compounds against TMV *in vivo*

To test viral inhibition, equal volumes of the virus (1.2 x 10^{-2} μg/mL) and the solution (1.0 mg/mL) of synthesized compounds or controls were mixed together for 30 min. The mixture was then inoculated into the growing *N. glutinosa*. leaves of the same age (4−5 leaves) by rubbing emery, and another pot was inoculated with the mixture of solvent and the virus by rubbing emery as the control. Then, the leaves were washed with water after 30 min. The local lesion numbers were recorded 2−3 days after inoculation. There are three replicates for each compound.

1.2 Curative Effect of Compounds against TMV *in vivo*

TMV (6 x 10^{-3} μg/mL) was inoculated on the growing leaves of *N. glutinosa*. of the same age by rubbing emery. Then, the leaves were washed with water and dried. The solution (500 μg/mL) of synthesized compounds or controls was smeared on the inoculated leaves, while inoculated leaves in another pot were smeared with the solvent as a control. The local lesion numbers were recorded 2−3 days after inoculation. There are three replicates for each compound.

1.3 Protective Effect of Compounds against TMV *in vivo*

The solution (500 μg/mL) of synthesized compounds and ningnanmycin was sprayed on growing *N. glutinosa*. leaves of the same age. In another pot, the leaves were sprayed with the solvent as a control. The leaves were then inoculated with the virus after 24 h by rubbing emery, and then wash the leaves with clear water after 30 min. The total local lesion numbers appearing on the leaves 2−3 days after inoculation were recorded. There are three replicates for each compound.

1.4 Determination of EC_{50} Values

Taking the process of EC_{50} value testing for the target $\mathbf{E_2}$ as an example, 4 mg compound $\mathbf{E_2}$ was accurately weighed and dissolved in 80 μL of DMSO to give a mother liquor. Then, 40 μL solution

was taken out and mixed with 4 mL 0.1% TW-80 water to give the first concentration (500 μg/mL); after adding 40 μL DMSO to the remaining 40 μL of solution, 40 μL solution was taken out and mixed with another 4 mL 0.1% TW-80 water to give the second concentration (250 μg/mL). According to the same operation to prepare solutions of different concentrations. And the EC_{50} values of target compounds against TMV were measured. There are three replicates for each compound.

The calculation formula for the inhibition rate of the test compound against TMV is as follows:

$Y = (C–A)/C \times 100\%$.

Y: the inhibitory rate of the compound against plant viruses; C: the number of dead spots in the control group (the left half of the leaf); A: the number of dead spots in the compound treatment group (the right half of the leaf).

2. Detailed Inhibition Rate of EC_{50} Values

The EC_{50} value is calculated by the linear equation that determines the inhibitory activity of compounds against viruses at five concentration gradients, respectively, and the inhibitory activity at different concentrations was shown in Table S1 and Table S2.

Table S1. The EC_{50} values of some title compounds for curative activity [a] (μg/mL).

NO.	Inhibition rate ± SD (%)				
	500	250	125	62.5	31.25
**E_2**	50.6±2.2	46.4±6.3	41.5±6.8	34.2±3.1	26.5±2.3
**E_8**	59.2±6.6	45.4±3.4	39.4±4.8	31.6±2.7	29.8±3.1
**E_9**	50.6±4.1	46.7±7.6	36.4±5.5	32.0±5.5	27.8±2.4
**E_{20}**	52.2±6.6	46.9±4.8	39.1±3.2	35.6±4.6	28.5±2.6
**E_{21}**	50.5±5.6	46.1±4.8	40.0±4.2	35.8±3.4	31.2±4.3
NNM*	57.9±6.6	53.3±6.8	47.3±6.8	41.8±5.7	32.0±7.4

* ningnanmycin; [a] Each experiment was performed in triplicate.

Table S2. The EC_{50} values of some title compounds for protective activity [a] (μg/mL).

NO.	Inhibition rate ± SD (%)				
	500	250	125	62.5	31.25
**E_2**	65.1±4.1	48.1±7.4	41.0±5.4	36.5±4.8	32.3±3.7
**E_{10}**	50.3±4.5	44.8±2.4	37.8±6.4	32.9±4.4	28.0±4.9
**E_{14}**	63.0±5.0	46.9±3.3	39.4±5.1	32.7±5.4	27.4±4.0
**E_{17}**	61.3±4.3	44.4±5.9	40.5±5.2	36.4±7.3	30.5±6.4
**E_{21}**	56.6±3.7	41.5±3.4	37.4±2.3	32.5±5.1	27.8±4.8
NNM*	56.0±3.6	48.7±5.2	43.5±3.8	38.9±5.8	34.2±3.9

* ningnanmycin; [a] Each experiment was performed in triplicate.

3. Crystallographic Data of Title Compound E_3

Table S3. The crystal data of compound **E_3**.

Empirical formula	$C_{18}H_{14}ClN_5S_2$
Formula weight	399.91
Temperature/K	273.15
Crystal system	monoclinic
Space group	$P2_1/c$
a/Å	20.6463(17)
b/Å	7.6779(7)
c/Å	11.7492(11)
α/°	90
β/°	90.755(4)
γ/°	90
Volume/Å^3	1862.3(3)
Z	4
ρ_{calc}g/cm^3	1.426
μ/mm^{-1}	4.011
F (000)	824.0
Crystal size/mm^3	0.23 × 0.22 × 0.19

Radiation	CuKα (λ = 1.54178)
2Θ range for data collection/°	8.566 to 139.782
Index ranges	$-25 \leq h \leq 25$, $-9 \leq k \leq 6$, $-14 \leq l \leq 14$
Reflections collected	15356
Independent reflections	3506 [R_{int} = 0.1163, R_{sigma} = 0.2355]
Data/restraints/parameters	3506/0/236
Goodness-of-fit on F^2	1.062
Final R indexes [I>=2σ (I)]	R_1 = 0.0734, wR_2 = 0.1998
Final R indexes [all data]	R_1 = 0.1821, wR_2 = 0.2243
Largest diff. peak/hole / e $Å^{-3}$	0.38/-0.61

4. Protective and Curative Activity Images of Title Compounds

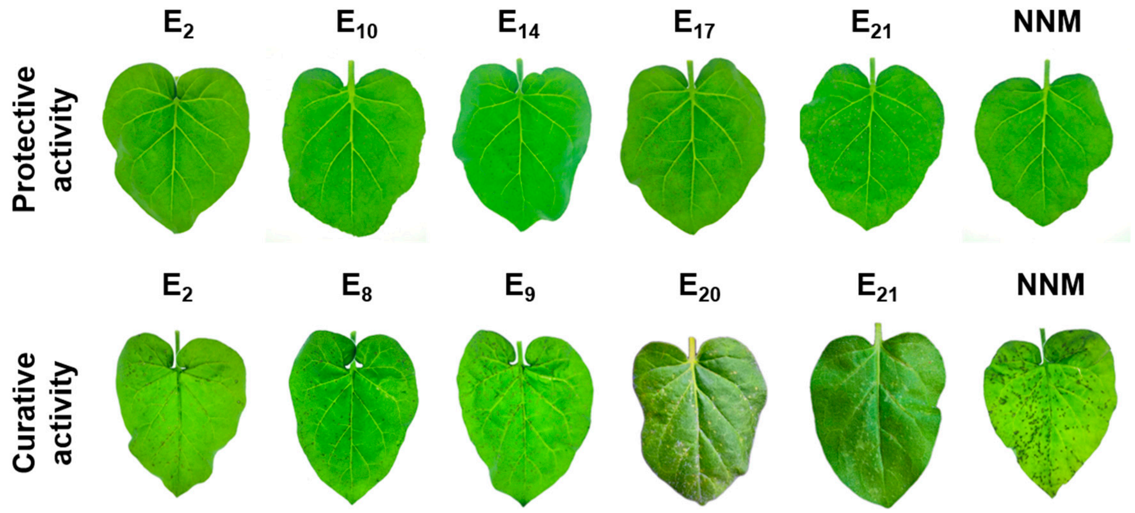

Figure S1. The protective and curative activity of some title compounds at 500 μg/mL.

(NNM: Ningnanmycin; Right leaf: with the treatment of compounds; Left leaf: with the treatment of PBS.)

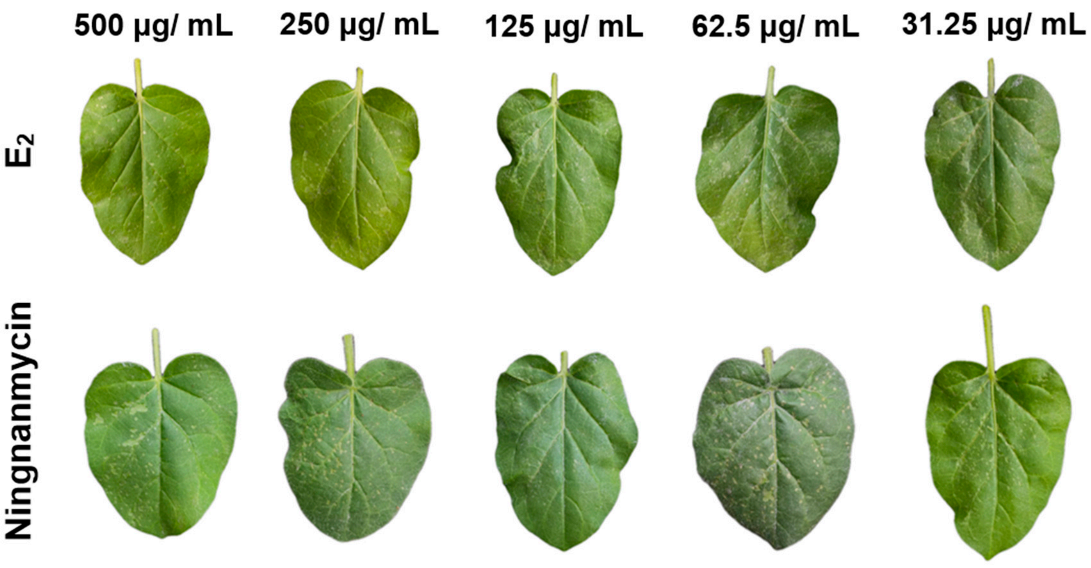

Figure S2. Protective activity images of title compound $\mathbf{E_2}$ and ningnanmycin at different concentrations.

(Right leaf: with the treatment of compounds; Left leaf: with the treatment of PBS.)

5. ^{1}H NMR, ^{13}C NMR and HRMS Data of Intermediates and Title Compounds

date for **B**: light yellow solid, yield 94%, m.p. 91–92 ºC. ^{1}H NMR (400 MHz, $CDCl_3$) δ 7.71 (s, 1H, benzene H), 7.53–7.40 (m, 4H, benzene H), 7.39–7.31 (m, 1H, benzene H), 5.34 (s, 2H, NH_2), 4.25 (q, *J* = 7.1 Hz, 2H, $\underline{CH_2}CH_3$), 1.32 (t, *J* = 7.1 Hz, 3H, $CH_2\underline{CH_3}$); ^{13}C NMR (101 MHz, $CDCl_3$) δ 164.41, 148.92, 140.47, 137.43, 129.57, 127.93, 123.61, 95.95, 59.50, 14.38; HRMS (ESI): m/z calcd for $C_{12}H_{14}O_2N_3$ $[M+H]^+$, 232.10805; found, 232.10805.

date for **C**: white solid, yield 80%, m.p.182–183 ºC. ^{1}H NMR (400 MHz, DMSO-d_6) δ 9.16 (s, 1H, $\underline{NH}NH_2$), 7.89 (s, 1H, pyrazole H), 7.57–7.50 (m, 4H, benzene H), 7.40 (t, *J* = 16.0 Hz, 1H, benzene H), 6.33 (s, 2H, $NH\underline{NH_2}$), 4.27 (s, 2H, NH_2); ^{13}C NMR (101 MHz, DMSO-d_6) δ 164.88, 149.36, 138.65, 138.42, 129.85, 127.53, 123.52, 96.55; HRMS (ESI): m/z calcd for $C_{12}H_{12}ON_5$ $[M+H]^+$, 218.10364; found, 218.10362.

date for **D**: yellow solid, yield 50%, m.p. 210–211 ºC. ^{1}H NMR (400 MHz, DMSO-d_6) δ 14.44 (s, 1H, SH), 7.80 (s, 1H, pyrazole H), 7.56 (d, *J* = 4.0 Hz, 4H, benzene H), 7.46–7.41 (m, 1H, benzene H), 6.21 (s, 2H, NH_2); ^{13}C NMR (101 MHz, DMSO-d_6) δ 185.01, 155.69, 145.52, 139.45, 138.15, 130.00, 128.20, 124.13, 94.74; HRMS (ESI): m/z cald for $C_9H_{14}N_5S_2$ $[M+H]^+$, 256.06851; found, 256.06848.

date for **E_1**: light yellow solid, yield 72%, m.p. 155–156 ºC. ^{1}H NMR (400 MHz, $CDCl_3$) δ 8.32 (s, 1H, pyrazole H), 8.14 (dd, *J* = 8.2, 2.2 Hz, 1H, benzene H), 7.80 (d, *J* = 7.4 Hz, 1H, benzene H), 7.62–7.46 (m, 6H, benzene H), 7.42 (t, *J* = 7.2 Hz, 1H, benzene H), 5.64 (s, 2H, NH_2), 4.62 (s, 2H, CH_2); ^{13}C NMR (101 MHz, $CDCl_3$) δ 162.90, 158.32, 148.29, 144.71, 139.35, 138.59, 137.48, 135.20, 129.84, 129.64, 128.28, 123.98, 123.77, 122.83, 95.30, 37.06; HRMS (ESI): m/z calcd for $C_{19}H_{18}N_6O_2S_2$ $[M+H]^+$, 380.0998; found, 380.0989.

date for **E_2**: yellow solid, yield 52%, m.p. 134–135 ºC. ^{1}H NMR (400 MHz, $CDCl_3$) δ 7.62–7.56 (m, 3H, benzene H), 7.53 (t, *J* = 7.5 Hz, 3H, benzene H), 7.41 (t, *J* = 8.0 Hz, 2H, benzene H), 7.22 (dd, *J* = 14.0, 7.3 Hz, 2H, benzene H), 5.66 (s, 1H, NH_2), 4.66 (s, 1H, CH_2); ^{13}C NMR (101 MHz, $CDCl_3$) δ 162.74, 159.51, 144.69, 139.37, 137.58, 134.35, 133.98, 131.24, 129.80, 129.35, 128.21, 127.02, 123.77, 95.47, 36.12; HRMS (ESI): m/z calcd for $C_{18}H_{15}N_5ClS_2$ $[M+H]^+$, 400.0452; found, 400.0446.

date for **E_3**: white solid, yield 60%, m.p. 163–164 ºC. ^{1}H NMR (400 MHz, $CDCl_3$) δ 7.65 (s, 1H, pyrazole H), 7.55 (dt, *J* = 15.5, 7.7 Hz, 4H, benzene H), 7.43 (t, *J* = 7.1 Hz, 1H, benzene H), 7.36 (d,

J = 8.5 Hz, 2H, benzene H), 7.30 (d, *J* = 8.5 Hz, 2H, benzene H), 4.50 (s, 2H, CH_2); ^{13}C NMR (101 MHz, $CDCl_3$) δ 162.46, 159.61, 144.88, 139.04, 136.99, 134.46, 133.79, 130.45, 129.92, 128.90, 128.57, 123.95, 95.26, 37.67; HRMS (ESI): m/z calcd for $C_{18}H_{15}N_5ClS_2$ $[M+H]^+$, 400.0452; found, 400.0443.

date for **E_4**: light yellow solid, yield 60%, m.p. 140–141ºC. ^1H NMR (400 MHz, DMSO-d_6) δ 7.86 (s, 2H, pyrazole H), 7.61–7.54 (m, 5H, benzene H), 7.45–7.36 (m, 4H, benzene H), 6.55 (s, 2H, NH_2), 4.56 (s, 2H, CH_2); ^{13}C NMR (101 MHz, DMSO-d_6) δ 163.08, 159.04, 145.86, 140.24, 139.93, 138.35, 133.49, 130.88, 129.97, 129.32, 128.25, 128.06, 128.03, 123.95, 95.06, 37.43; HRMS (ESI): m/z calcd for $C_{18}H_{15}N_5ClS_2$ $[M+H]^+$, 400.0452; found, 400.0445.

date for **E_5**: light yellow solid, yield 49%, m.p. 129–130 ºC. ^1H NMR (400 MHz, $CDCl_3$) δ 7.65 (s, 1H, pyrazole H), 7.62–7.49 (m, 4H, benzene H), 7.44 (t, *J* = 7.2 Hz, 1H, benzene H), 7.36 (d, *J* = 7.3 Hz, 1H, benzene H), 7.25–7.13 (m, 3H, benzene H), 4.59 (s, 2H, CH_2), 2.45 (s, 3H, CH_3); ^{13}C NMR (101 MHz, $CDCl_3$) δ 162.34, 160.49, 144.96, 139.15, 137.21, 137.11, 133.25, 130.77, 130.28, 129.98, 128.61, 128.46, 126.41, 124.01, 95.33, 36.92, 19.28; HRMS (ESI): m/z calcd for $C_{19}H_{18}N_5S_2$ $[M+H]^+$, 380.0998; found, 380.0992.

date for **E_6**: white solid, yield 44%, m.p. 163–164 ºC. ^1H NMR (400 MHz, $CDCl_3$) δ 7.64 (s, 1H, pyrazole H), 7.55 (dt, *J* = 15.5, 7.7 Hz, 4H, benzene H), 7.43 (t, *J* = 7.2 Hz, 1H, benzene H), 7.31 (d, *J* = 8.0 Hz, 2H, benzene H), 7.14 (d, *J* = 7.9 Hz, 2H, benzene H), 5.43 (s, 2H, NH_2), 4.51 (s, 2H, CH_2), 2.34 (s, 3H, CH_3); ^{13}C NMR (101 MHz, $CDCl_3$) δ 162.27, 160.50, 144.94, 139.10, 137.85, 137.07, 132.59, 129.97, 129.52, 129.09, 128.61, 124.01, 95.34, 38.47, 21.21; HRMS (ESI): m/z calcd for $C_{19}H_{18}N_5S_2$ $[M+H]^+$, 380.0998; found, 380.0992.

date for **E_7**: yellow solid, yield 71%, m.p. 150–151 ºC. ^1H NMR (400 MHz, DMSO-d_6) δ 7.86 (s, 1H, pyrazole H), 7.65–7.53 (m, 4H, benzene H), 7.49 (dd, *J* = 8.8, 5.5 Hz, 2H, benzene H), 7.43 (t, *J* = 7.1 Hz, 1H, benzene H), 7.17 (t, *J* = 8.9 Hz, 2H, benzene H), 6.55 (s, 2H, NH_2), 4.54 (s, 2H, CH_2); ^{13}C NMR (101 MHz, DMSO-d_6) δ 163.23, 162.98, 160.80, 159.24, 145.84, 140.23, 138.36, 133.48–133.45 (d, *J* = 3.0 Hz), 131.66 (d, *J* = 8.0 Hz), 129.97, 128.03, 123.94, 115.97, 115.75, 95.08, 37.45; ^{19}F NMR (376 MHz, DMSO) δ -114.51 – -114.61 (m); HRMS (ESI): m/z calcd for $C_{18}H_{15}N_5FS_2$ $[M+H]^+$, 384.0708; found, 384.0747.

date for **E_8**: white solid, yield 67%, m.p. 107–108 ºC. ^1H NMR (400 MHz, $CDCl_3$) δ 7.63 (s, 1H,

pyrazole H), 7.54 (dt, *J* = 15.6, 7.7 Hz, 4H, benzene H), 7.42 (t, *J* = 7.2 Hz, 1H, benzene H), 7.25–7.20 (m, 1H, benzene H), 7.02–6.92 (m, 2H, benzene H), 6.83 (d, *J* = 8.2 Hz, 1H, benzene H), 4.51 (s, 2H, CH_2), 3.79 (s, 3H, OCH_3); ^{13}C NMR (101 MHz, $CDCl_3$) δ 162.39, 160.24, 159.85, 144.93, 139.14, 137.18, 129.97, 129.85, 128.58, 123.99, 121.47, 114.68, 114.61, 113.64, 95.34, 55.31, 38.63; HRMS (ESI): m/z calcd for $C_{19}H_{18}ON_5S_2$ $[M+H]^+$, 396.0947; found, 396.0940.

date for **E$_9$**: white solid, yield 66%, m.p. 157–158 ºC. ^1H NMR (400 MHz, DMSO-*d*$_6$) δ 7.86 (s, 1H, pyrazole H), 7.67–7.51 (m, 4H, benzene H), 7.43 (t, *J* = 7.2 Hz, 1H, benzene H), 7.37 (d, *J* = 8.7 Hz, 2H, benzene H), 6.90 (d, *J* = 8.7 Hz, 2H, benzene H), 6.56 (s, 2H, NH_2), 4.49 (s, 2H, CH_2), 3.73 (s, 3H, OCH_3); ^{13}C NMR (101 MHz, DMSO-*d*$_6$) δ 162.80, 159.63, 159.22, 145.83, 140.22, 138.37, 130.84, 129.97, 128.67, 128.01, 123.93, 114.44, 95.12, 55.55, 38.02; HRMS (ESI): m/z calcd for $C_{19}H_{18}ON_5S_2$ $[M+H]^+$, 396.0947; found, 396.0939.

date for **E$_{10}$**: yellow solid, yield 63%, m.p. 136–137 ºC. ^1H NMR (500 MHz, DMSO-*d*$_6$) δ 7.87 (s, 1H, pyrazole H), 7.75 (m, 2H, benzene H), 7.68 (t, *J* = 7.6 Hz, 1H, benzene H), 7.61 (d, *J* = 7.3 Hz, 2H, benzene H), 7.55 (m, 3H, benzene H), 7.43 (t, *J* = 7.3 Hz, 1H, benzene H), 6.57 (s, 2H, NH_2), 4.71 (s, 2H, CH_2); ^{13}C NMR (126 MHz, DMSO-*d*$_6$) δ 162.94, 158.03, 145.45, 139.80, 137.88, 134.54, 133.01, 131.98, 129.49, 128.57, 127.56, 127.30, 127.06, 126.34, 125.37, 123.49, 123.19, 94.59, 34.74; ^{19}F NMR (376 MHz, DMSO-*d*$_6$) δ -59.32 – -59.54 (d, *J* = 80.4 Hz); HRMS (ESI): m/z calcd for $C_{19}H_{15}F_3N_5S_2$ $[M+H]^+$, 434.0716; found, 434.0707.

date for **E$_{11}$**: white solid, yield 64%, m.p. 154–155 ºC. ^1H NMR (400 MHz, $CDCl_3$) δ 7.69 (s, 1H, pyrazole H), 7.63 (d, *J* = 4.0 Hz, 2H, benzene H), 7.56 (dt, *J* = 15.6, 7.8 Hz, 5H, benzene H), 7.5 (dd, *J* = 18.3, 7.5 Hz, 2H, benzene H), 4.59 (s, 2H, CH_2); ^{13}C NMR (101 MHz, $CDCl_3$) δ 162.65, 159.25, 144.91, 139.14, 137.15, 132.54, 129.97, 129.29, 128.57, 125.92, 125.88, 124.81, 124.78, 123.98, 95.35, 37.74; ^{19}F NMR (376 MHz, $CDCl_3$) δ -62.63 (s); HRMS (ESI): m/z calcd for $C_{19}H_{15}F_3N_5S_2$ $[M+H]^+$, 434.0716; found, 434.0708.

date for **E$_{12}$**: white solid, yield 70%, m.p. 173–174 ºC. ^1H NMR (400 MHz, $CDCl_3$) δ 7.66 (s, 1H, pyrazole H), 7.57 (m, 8H, benzene H), 7.44 (t, *J* = 7.0 Hz, 1H, benzene H), 5.46 (s, 2H, NH_2), 4.58 (s, 2H, CH_2); ^{13}C NMR (101 MHz, $CDCl_3$) δ 162.56, 159.44, 144.98, 140.20, 138.99, 136.88, 130.02, 129.48, 128.75, 125.80–125.69 (*J* = 11.0 Hz), 124.06, 95.26, 37.62; ^{19}F NMR (376 MHz, $CDCl_3$) δ -62.59 (s); HRMS (ESI): m/z calcd for $C_{19}H_{15}F_3N_5S_2$ $[M+H]^+$, 434.0716; found, 434.0703.

date for **E_{13}**: yellow solid, yield 62%, m.p. 116–117 ºC. ^1H NMR (400 MHz, $CDCl_3$) δ 7.62 (s, 1H, pyrazole H), 7.58 (d, *J* = 7.4 Hz, 2H, benzene H), 7.52 (t, *J* = 7.6 Hz, 2H, benzene H), 7.41 (t, *J* = 7.3 Hz, 1H, benzene H), 6.05–5.95 (m, 1H, $\underline{CH}$=CH_2), 5.66 (s, 2H, NH_2), 5.36 (d, *J* = 16.0 Hz, 1H, CH=$\underline{CH_2}$), 3.93 (d, *J* = 8.0 Hz, 2H, SCH_2); ^{13}C NMR (101 MHz, $CDCl_3$) δ 162.68, 159.63, 144.77, 139.42, 137.66, 132.17, 129.86, 128.25, 123.81, 119.47, 95.55, 37.15; HRMS (ESI): m/z calcd for $C_{14}H_{14}N_5S_2$ $[M+H]^+$, 316.0686; found, 316.0678.

date for **E_{14}**: light yellow solid, yield 61%, m.p. 165–166 ºC. ^1H NMR (400 MHz, $CDCl_3$) δ 7.66 (s, 1H, pyrazole H), 7.61–7.50 (m, 4H, benzene H), 7.44 (t, *J* = 7.2 Hz, 1H, benzene H), 5.41 (s, 2H, NH_2), 3.33 (q, *J* = 7.4 Hz, 2H, $CH_3\underline{CH_2}$), 1.48 (t, *J* = 7.4 Hz, 3H, $\underline{CH_3}CH_2$); ^{13}C NMR (101 MHz, $CDCl_3$) δ 162.05, 161.02, 144.91, 139.14, 137.15, 129.95, 128.56, 123.99, 95.34, 28.82, 14.65; HRMS (ESI): m/z calcd for $C_{13}H_{14}N_5S_2$ $[M+H]^+$, 304.0686; found, 304.0678.

date for **E_{15}**: light yellow solid, yield 52%, m.p. 156–157 ºC. ^1H NMR (400 MHz, DMSO-d_6) δ 7.86 (s, 1H, pyrazole H), 7.63–7.52 (m, 5H, benzene H), 7.48–7.41 (m, 3H, benzene H), 7.35 (t, *J* = 7.2 Hz, 2H, benzene H), 7.29 (t, *J* = 7.2 Hz, 1H, benzene H), 6.56 (s, 2H, NH_2), 4.55 (s, 2H, CH_2); ^{13}C NMR (101 MHz, DMSO-d_6) δ 162.90, 159.47, 145.84, 140.22, 138.36, 137.06, 129.97, 129.55, 129.06, 128.14, 128.02, 123.94, 95.09, 78.37, 38.34; HRMS (ESI): m/z calcd for $C_{18}H_{18}N_5S_2$ $[M+H]^+$, 366.0842; found, 366.0837.

date for **E_{16}**: white solid, yield 46%, m.p. 131–132 ºC. ^1H NMR (400 MHz, DMSO-d_6) δ 7.87 (s, 1H, pyrazole H), 7.64–7.48 (m, 5H, benzene H), 7.46–7.32 (m, 2H, benzene H), 7.27–7.15 (m, 2H, benzene H), 6.56 (s, 2H, NH_2), 4.56 (s, 2H, CH_2); ^{13}C NMR (101 MHz, DMSO-d_6) δ 163.36, 162.11, 159.66, 158.68, 145.88, 140.26, 138.35, 131.91, 131.87, 130.60, 130.51, 129.97, 128.03, 125.09, 125.05, 124.19, 123.96, 116.11, 115.90, 95.08, 32.15; ^{19}F NMR (376 MHz, DMSO-d_6) δ -116.85 – -116.93 (m); HRMS (ESI): m/z calcd for $C_{18}H_{14}FN_5S_2$ $[M+H]^+$, 384.0747; found, 384.0746.

date for **E_{17}**: white solid, yield 65%, m.p. 135–136 ºC. ^1H NMR (400 MHz, DMSO-d_6) δ 7.87 (s, 1H, pyrazole H), 7.61–7.54 (m, 4H, benzene H), 7.45 (t, *J* = 16.0 Hz, 1H, benzene H), 7.38 (d, *J* = 8.0 Hz, 1H, benzene H), 7.32 (t, *J* = 16.0 Hz, 1H, benzene H), 7.04 (d, *J* = 8.0 Hz, 1H, benzene H), 6.92 (t, *J* = 16.0 Hz, 1H, benzene H), 6.55 (s, 2H, NH_2), 4.47 (s, 2H, CH_2), 3.82 (s, 3H, OCH_3); ^{13}C NMR (101 MHz, DMSO-d_6) δ 162.89, 160.00, 157.63, 145.80, 140.24, 138.33, 130.90, 130.01, 129.98, 128.04, 124.45, 123.93, 120.80, 111.57, 95.11, 56.01, 33.88; HRMS (ESI): m/z calcd for $C_{19}H_{18}N_5OS_2$

[M+H]$^+$, 396.0947; found, 396.0947.

date for **E$_{18}$**: yellow solid, yield 81%, m.p. 157–158 ºC. ^{1}H NMR (400 MHz, DMSO-d_6) δ 7.86 (s, 1H, pyrazole H), 7.57 (dt, J = 15.6, 7.7 Hz, 4H, benzene H), 7.47–7.34 (m, 2H, benzene H), 7.32–7.26 (m, 2H, benzene H), 7.12 (t, J = 9.9 Hz, 1H, benzene H), 6.55 (s, 2H, NH_2), 4.56 (s, 2H, CH_2); ^{13}C NMR (101 MHz, DMSO-d_6) δ 163.08, 159.13, 145.83, 140.24, 138.30, 131.04, 130.95, 129.99, 128.06, 125.66, 123.95, 116.36, 116.14, 115.08, 114.87, 95.04, 37.56; ^{19}F NMR (376 MHz, DMSO-d_6) δ -112.92 – -113.04 (m); HRMS (ESI): m/z calcd for $C_{18}H_{15}FN_5S_2$ [M+H]$^+$, 384.0747; found, 384.0744.

date for **E$_{19}$**: white solid, yield 80%, m.p. 140–141 ºC. ^{1}H NMR (400 MHz, DMSO-d_6) δ 7.87 (s, 1H, pyrazole H), 7.67 (d, J = 7.9 Hz, 1H, benzene H), 7.57 (dt, J = 15.7, 7.9 Hz, 5H, benzene H), 7.43 (t, J = 7.1 Hz, 1H, benzene H), 7.36 (t, J = 8.1 Hz, 1H, benzene H), 7.26 (t, J = 8.5 Hz, 1H, benzene H), 6.56 (s, 2H, NH_2), 4.61 (s, 2H, CH_2); ^{13}C NMR (101 MHz, DMSO-d_6) δ 163.32, 158.70, 145.85, 140.28, 138.29, 136.05, 133.41, 132.01, 130.51, 129.99, 128.51, 128.07, 124.57, 123.95, 95.06; HRMS (ESI): m/z calcd for $C_{18}H_{15}BrN_5S_2$ [M+H]$^+$, 443.9947; found, 443.9943.

date for **E$_{20}$**: white solid, yield 80%, m.p. 146–147 ºC. ^{1}H NMR (400 MHz, DMSO-d_6) δ 7.86 (s, 1H, pyrazole H), 7.68 (s, 1H, benzene H), 7.60–7.53 (m, 4H, benzene H), 7.45 (dt, J = 12.4, 7.5 Hz, 3H, benzene H), 7.30 (t, J = 7.8 Hz, 1H, benzene H), 6.55 (s, 2H, NH_2), 4.55 (s, 2H, CH_2); ^{13}C NMR (101 MHz, DMSO-d_6) δ 163.08, 159.05, 145.84, 140.25, 140.20, 138.32, 132.19, 131.16, 130.94, 129.98, 128.62, 128.04, 123.95, 122.09, 95.05, 37.35; HRMS (ESI): m/z calcd for $C_{18}H_{15}BrN_5S_2$ [M+H]$^+$, 443.9947; found, 443.9947.

date for **E$_{21}$**: white solid, yield 56%, m.p. 166–167 ºC. ^{1}H NMR (400 MHz, DMSO-d_6) δ 7.86 (s, 1H, pyrazole H), 7.61–7.53 (m, 6H, benzene H), 7.53–7.39 (m, 3H, benzene H), 6.55 (s, 2H, NH_2), 4.52 (s, 2H, CH_2); ^{13}C NMR (101 MHz, DMSO-d_6) δ 163.04, 159.11, 145.83, 140.25, 138.32, 136.87, 131.92, 131.73, 129.98, 128.05, 123.94, 121.25, 95.06, 37.47; HRMS (ESI): m/z calcd for $C_{18}H_{15}BrN_5S_2$ [M+H]$^+$, 443.9947; found, 443.9945.

date for **E$_{22}$**: yellow solid, yield 72%, m.p. 113–114 ºC. ^{1}H NMR (400 MHz, DMSO-d_6) δ 7.88 (s, 1H, pyrazole H), 7.58 (dt, J = 13.4, 6.7 Hz, 4H, benzene H), 7.45 (t, J = 7.2 Hz, 1H, benzene H), 6.56 (s, 2H, NH_2), 3.19 (d, J = 6.8 Hz, 2H, CH_2), 2.04–1.96 (m, 1H, $\underline{CH}$ $(CH_3)_2$), 1.03–1.01 (d, J = 8.0 Hz, 6H, CH $\underline{(CH_3)_2}$); ^{13}C NMR (101 MHz, DMSO-d_6) δ 162.40, 160.66, 145.79, 140.21, 138.38, 129.96, 127.99, 123.89, 95.12, 42.82, 28.60, 21.89; HRMS (ESI): m/z calcd for $C_{15}H_{18}N_5S_2$ [M+H]$^+$, 322.0998;

found, 322.0996.

date for **E_{23}**: yellow solid, yield 73%, m.p. 116–117 ºC. ^{1}H NMR (400 MHz, $CDCl_3$) δ 7.61 (s, 1H, pyrazole H), 7.60 (d, *J* = 8.5 Hz, 2H, benzene H), 7.54 (t, *J* = 7.8 Hz, 2H, benzene H), 7.42 (t, *J* = 7.3 Hz, 1H, benzene H), 5.65 (s, 2H, NH_2), 3.30 (t, *J* = 12.0 Hz, 2H, CH_2), 1.90–1.81 (m, 2H, $\underline{CH_2}CH_3$), 1.07 (t, *J* = 7.4 Hz, 3H, $CH_2\underline{CH_3}$); ^{13}C NMR (101 MHz, $CDCl_3$) δ 162.10, 160.90, 144.67, 139.35, 137.62, 129.82, 128.19, 123.75, 95.52, 36.22, 22.72, 13.34; HRMS (ESI): m/z calcd for $C_{14}H_{16}N_5S_2$ $[M+H]^+$, 318.0842; found, 318.0841.

date for **E_{24}**: light yellow solid, yield 60%, m.p. 200–201 ºC. ^{1}H NMR (400 MHz, $CDCl_3$) δ 8.21 (d, *J* = 12.0 Hz, 2H, benzene H), 7.64 (s, 1H, pyrazole H), 7.62 (d, *J* = 4.0 Hz, 2H, benzene H), 7.59–7.52 (m, 4H, benzene H), 7.45–7.41 (m, 1H, benzene H), 5.64 (s, 2H, NH_2), 4.61 (s, 2H, CH_2); ^{13}C NMR (101 MHz, $CDCl_3$) δ 163.07, 158.26, 147.43, 144.77, 144.08, 139.42, 137.55, 130.02, 129.92, 128.38, 123.96, 123.85, 95.36, 37.19; HRMS (ESI): m/z calcd for $C_{18}H_{15}N_6O_2S_2$ $[M+H]^+$, 411.0692; found, 411.0686.

date for **E_{25}**: white solid, yield 44%, m.p. 162–163 ºC. ^{1}H NMR (400 MHz, $CDCl_3$) δ 8.10 (d, *J* = 8.2 Hz, 1H, pyrazole H), 7.74 (d, *J* = 7.7 Hz, 1H, benzene H), 7.57 (d, *J* = 7.4 Hz, 4H, benzene H), 7.53 (t, *J* = 7.3 Hz, 2H, benzene H), 7.47–7.38 (m, 2H, benzene H), 5.64 (s, 2H, NH_2), 4.88 (s, 2H, CH_2); ^{13}C NMR (101 MHz, $CDCl_3$) δ 162.92, 159.48, 147.85, 144.71, 139.43, 137.60, 133.84, 133.00, 132.83, 129.87, 129.07, 128.28, 125.61, 123.79, 95.45, 35.40; HRMS (ESI): m/z calcd for $C_{18}H_{15}N_6O_2S_2$ $[M+H]^+$, 411.0692; found, 411.0686.

date for **E_{26}**: light yellow solid, yield 83%, m.p. 176–177 ºC. ^{1}H NMR (400 MHz, $CDCl_3$) δ 8.32 (s, 1H, pyrazole H), 8.14 (dd, *J* = 8.2, 2.2 Hz, 1H, benzene H), 7.80 (d, *J* = 7.4 Hz, 1H, benzene H), 7.62–7.46 (m, 6H, benzene H), 7.42 (t, *J* = 7.2 Hz, 1H, benzene H), 5.64 (s, 2H, NH_2), 4.62 (s, 2H, CH_2); ^{13}C NMR (101 MHz, $CDCl_3$) δ 162.51, 160.05, 144.77, 139.41, 138.55, 137.67, 135.70, 129.86, 128.75, 128.69, 128.25, 126.21, 123.83, 95.55, 38.62, 21.39; HRMS (ESI): m/z calcd for $C_{19}H_{15}N_5O_2S_2$ $[M+H]^+$, 411.0692; found, 380.0691.

date for **E_{27}**: white solid, yield 66%, m.p. 168–169 ºC. ^{1}H NMR (400 MHz, $CDCl_3$) δ 7.61 (s, 1H, pyrazole H), 7.59 (d, *J* = 8.5 Hz, 2H, benzene H), 7.53 (t, *J* = 7.8 Hz, 2H, benzene H), 7.42 (t, *J* = 7.3 Hz, 1H, benzene H), 5.66 (s, 2H, NH_2) 2.78 (s, 3H, CH_3); ^{13}C NMR (101 MHz, $CDCl_3$) δ 161.97, 161.65, 144.68, 139.30, 137.61, 129.80, 128.18, 123.74, 95.45, 16.48; HRMS (ESI): m/z calcd for

$C_{12}H_{12}N_5S_2$ [M+H]$^+$, 290.0529; found, 290.0531.

date for **E_{28}**: white solid, yield 73%, m.p. 122–123 ºC. ^{1}H NMR (500 MHz, DMSO-d_6) δ 7.87 (s, 1H, pyrazole H), 7.57 (d, J = 20.9 Hz, 4H, benzene H), 7.44 (s, 1H, benzene H), 6.53 (s, 2H, NH_2), 3.26 (s, 2H, SCH_2), 1.72 (s, 2H, $\underline{CH_2}(CH_2)_2CH_3$), 1.35 (d, J = 33.5 Hz, 2H, $CH_2\underline{CH_2}CH_2CH_3$), 1.35 (d, J = 33.5 Hz, 2H, $CH_2CH_2\underline{CH_2}CH_3$), 0.86 (s, 3H, $CH_2CH_2CH_2\underline{CH_3}$); ^{13}C NMR (101 MHz, DMSO-d_6) δ 162.05, 159.99, 145.41, 139.81, 138.01, 129.55, 127.58, 123.51, 94.76, 34.11, 30.25, 28.67, 21.68, 13.90; HRMS (ESI): m/z calcd for $C_{16}H_{20}N_5S_2$ [M+H]$^+$, 346.1154; found, 346.1154.

6. Copies of Intermediates and Title Compounds

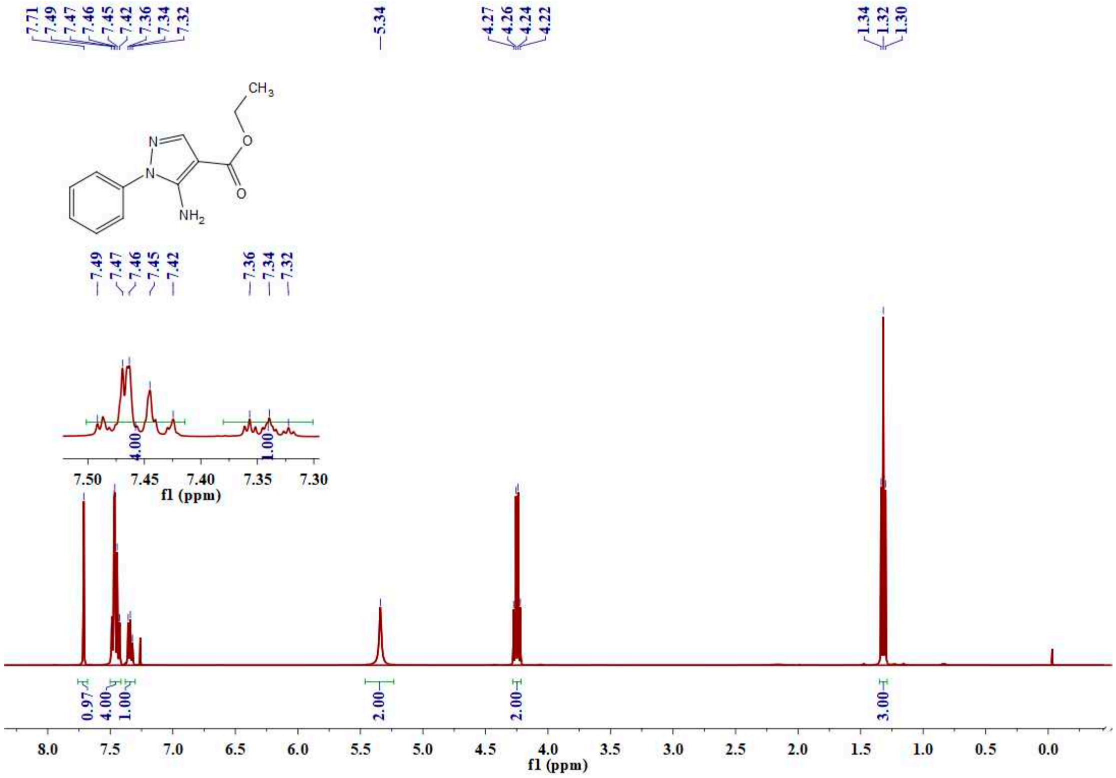

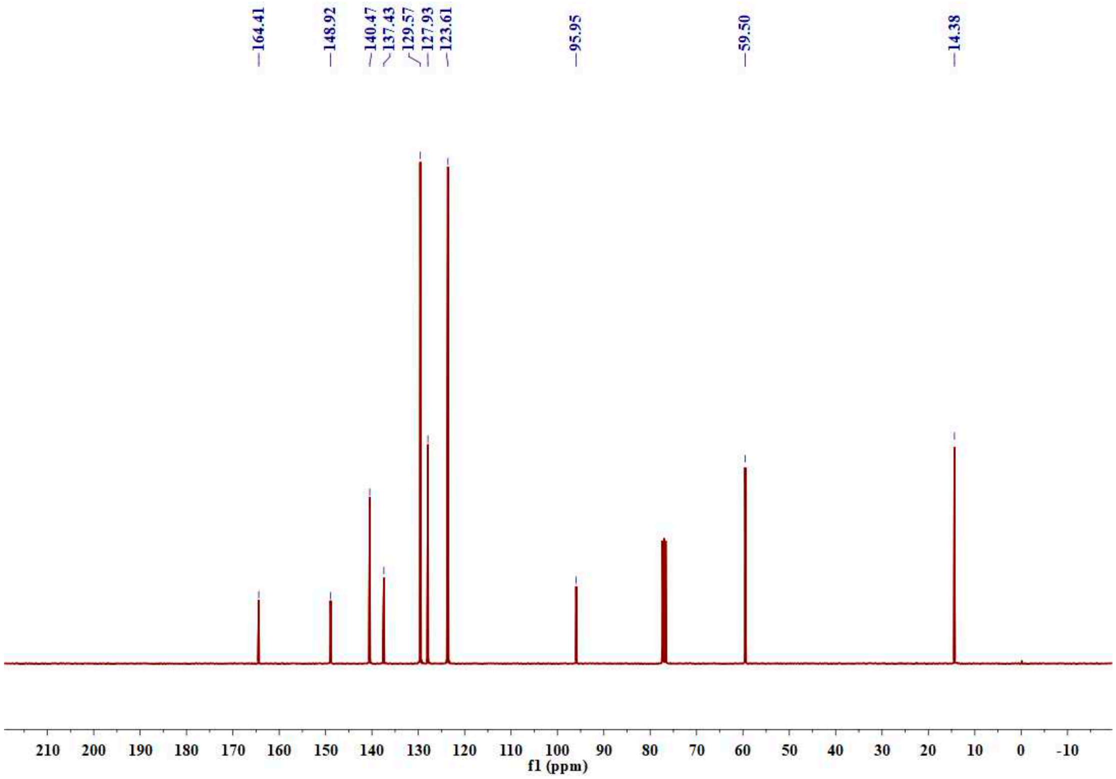

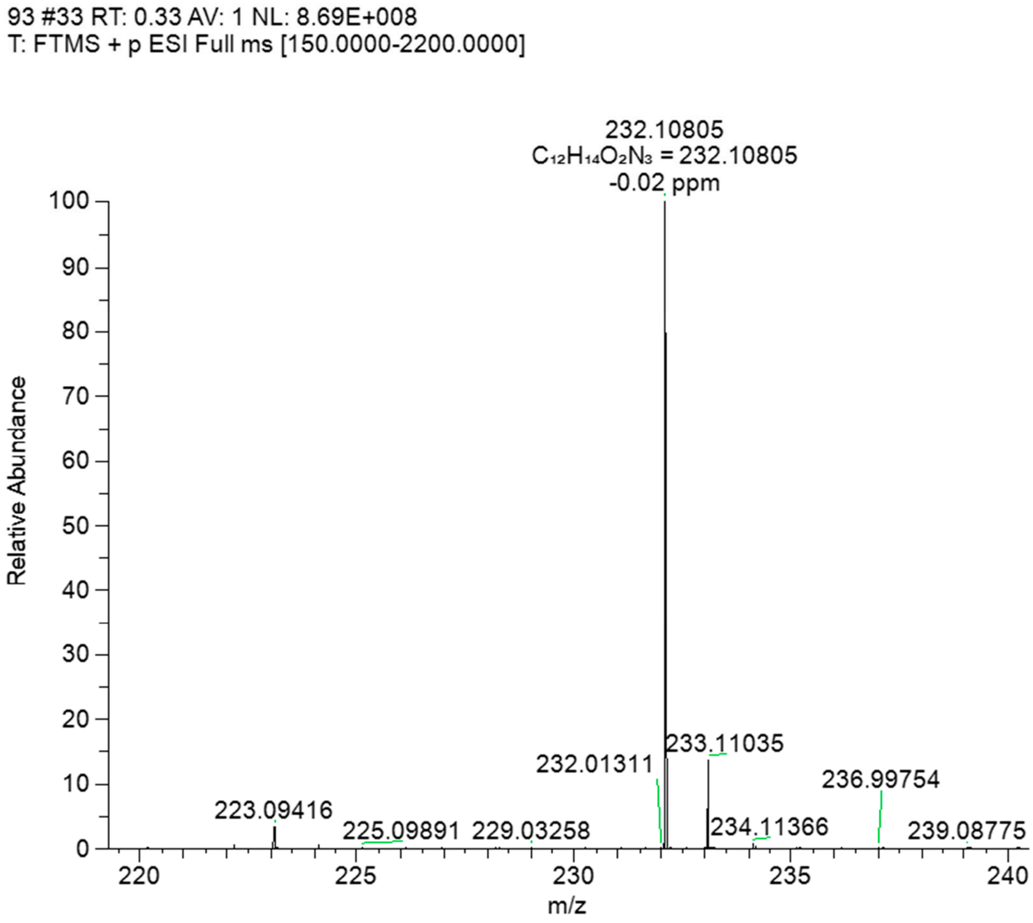

Figure S3. ^{1}H NMR, ^{13}C NMR, HRMS for intermediate **B**.

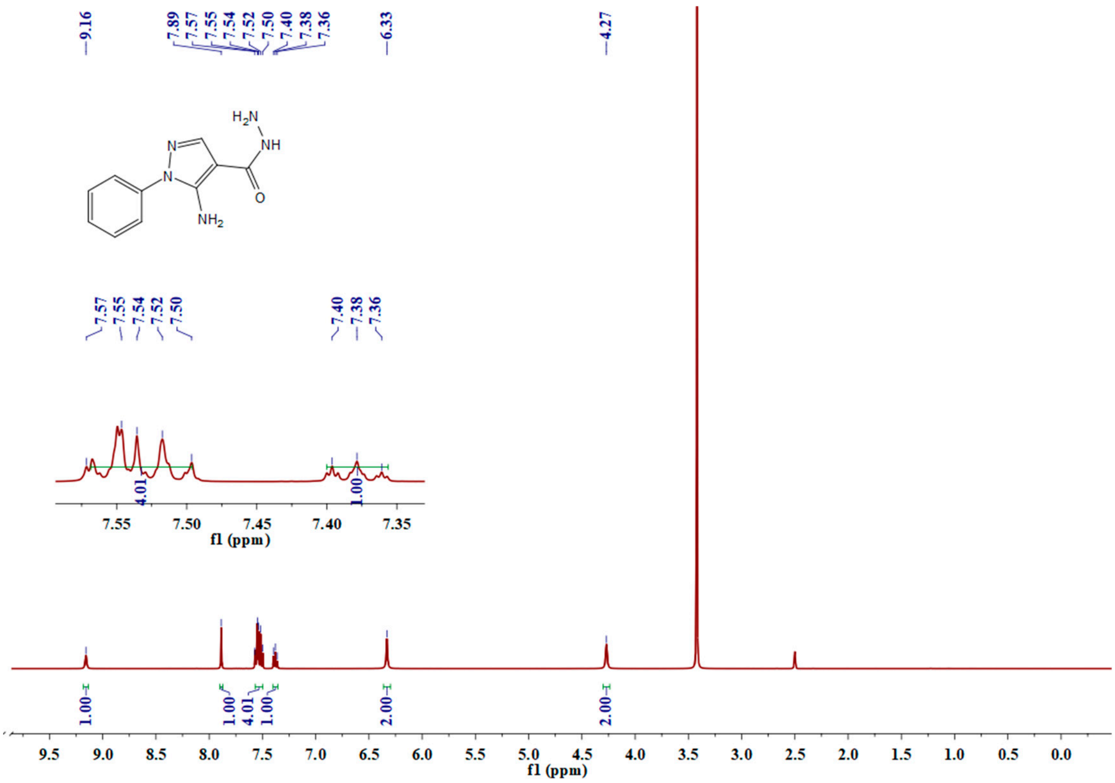
9.16
7.89
7.57
7.55
7.54
7.52
7.50
7.40
7.38
7.36
6.33
4.27
H2N
NH
N
N
O
NH2
1.00
4.01
f1 (ppm)
2.00
9.5 9.0 8.5 8.0 7.5 7.0 6.5 6.0 5.5 5.0 4.5 4.0 3.5 3.0 2.5 2.0 1.5 1.0 0.5 0.0

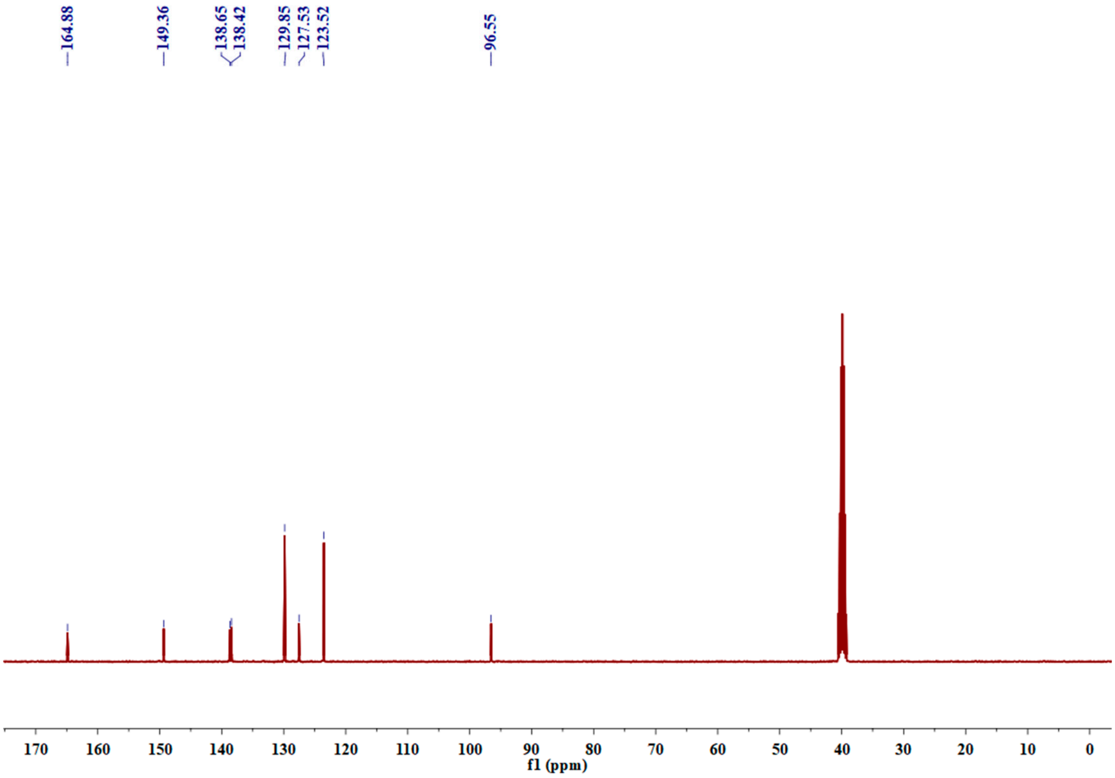
164.88
149.36
138.65
138.42
129.85
127.53
123.52
96.55
170 160 150 140 130 120 110 100 90 80 70 60 50 40 30 20 10 0
f1 (ppm)

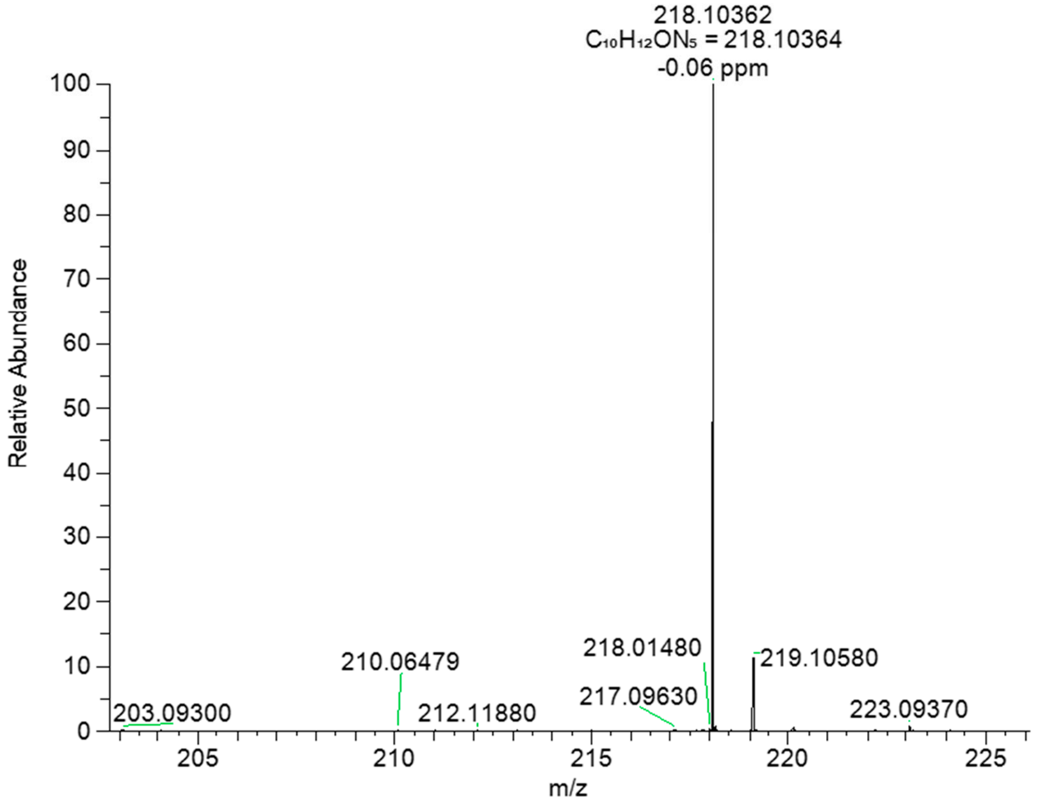

Figure S4. ^{1}H NMR, ^{13}C NMR, HRMS for intermediate **C**.

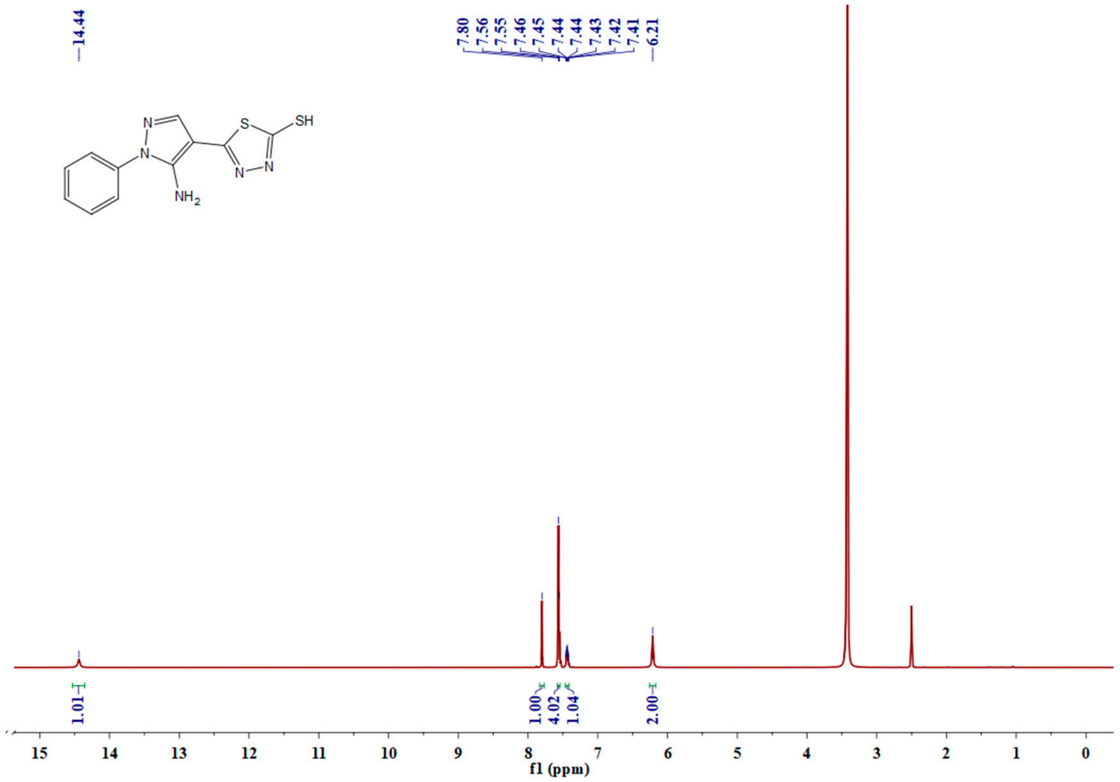

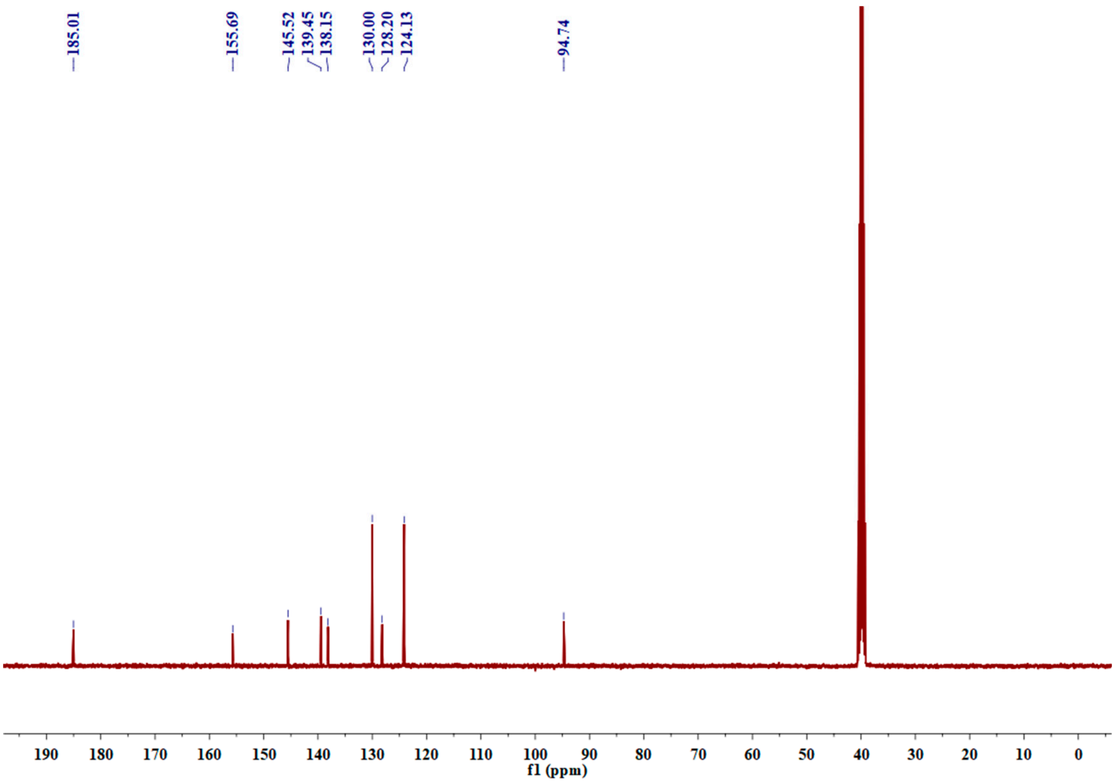

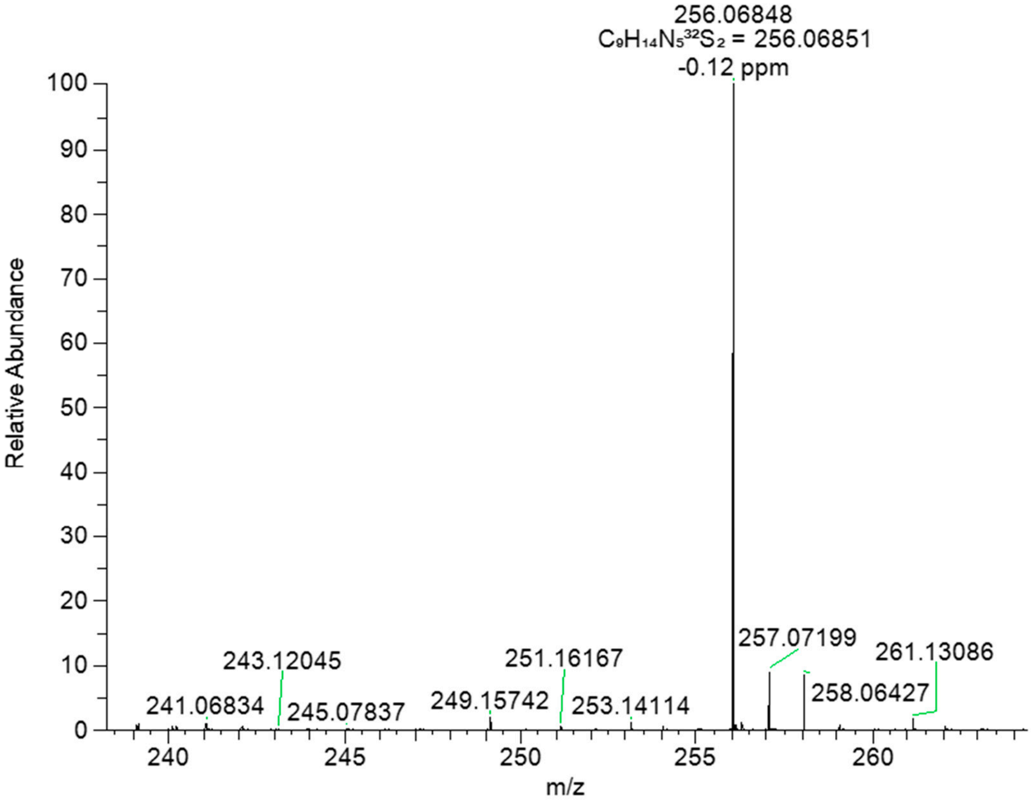

Figure S5. ^{1}H NMR, ^{13}C NMR, HRMS for intermediate **D**.

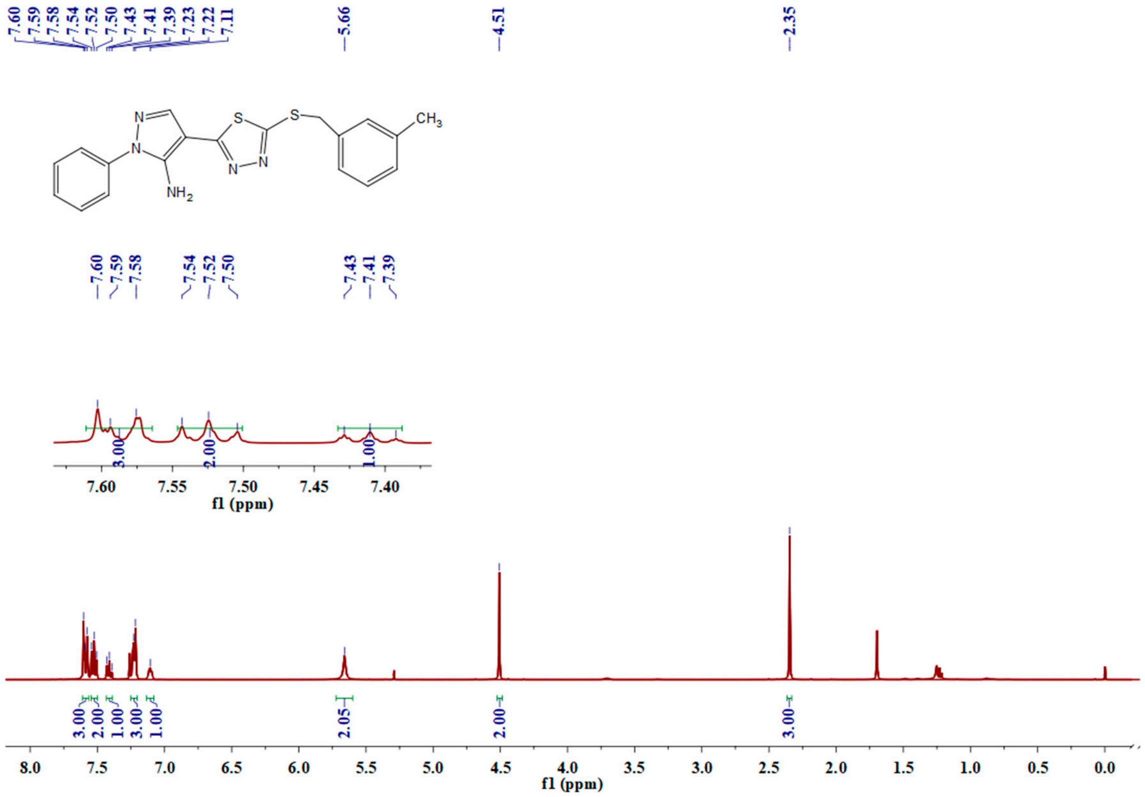
7.60 7.59 7.58 7.54 7.52 7.50 7.43 7.41 7.39 7.23 7.22 7.11
5.66
4.51
2.35
N N S S N N CH_3 NH_2
7.60 7.59 7.58 7.54 7.52 7.50 7.43 7.41 7.39
3.00 2.00 1.00
7.60 7.55 7.50 7.45 7.40
f1 (ppm)
3.00 2.00 1.00 3.00 1.00 2.05 2.00 3.00
8.0 7.5 7.0 6.5 6.0 5.5 5.0 4.5 4.0 3.5 3.0 2.5 2.0 1.5 1.0 0.5 0.0
f1 (ppm)

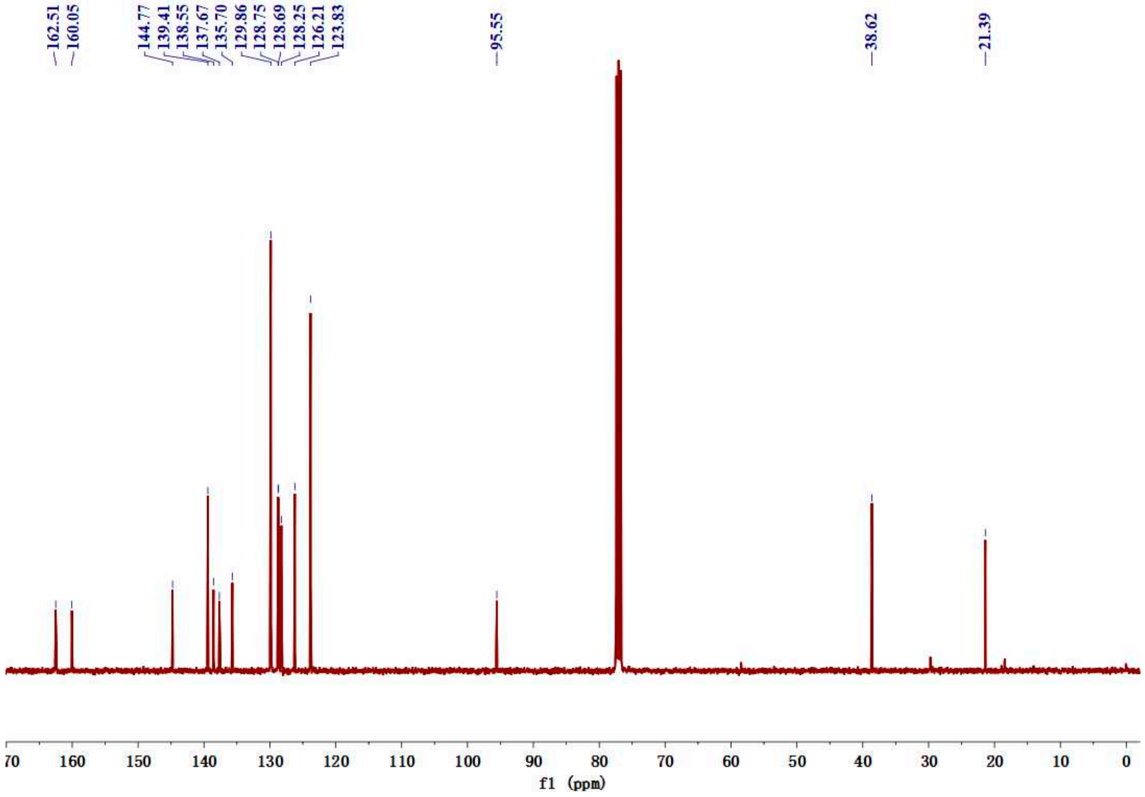
162.51 160.05
144.77 139.41 138.55 137.67 135.70 129.86 128.75 128.69 128.25 126.21 123.83
95.55
38.62
21.39
70 160 150 140 130 120 110 100 90 80 70 60 50 40 30 20 10 0
f1 (ppm)

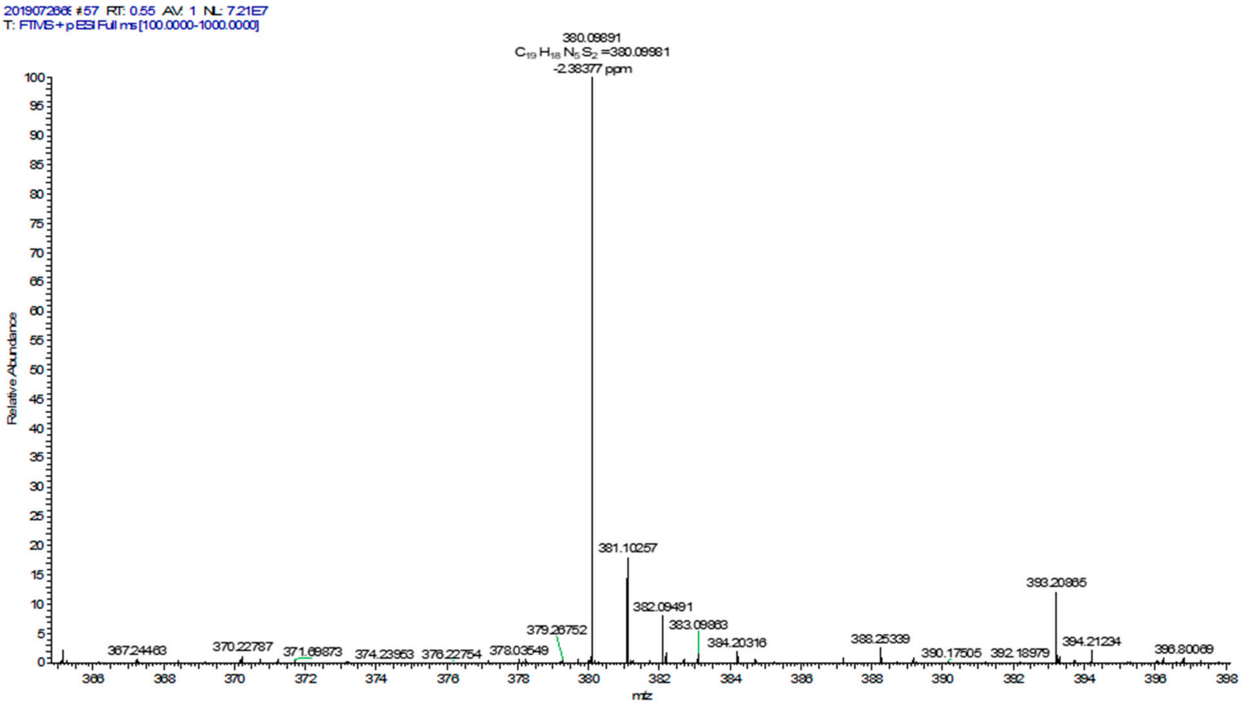

Figure S6. ^{1}H NMR, ^{13}C NMR and HRMS for title compound $\mathbf{E_1}$.

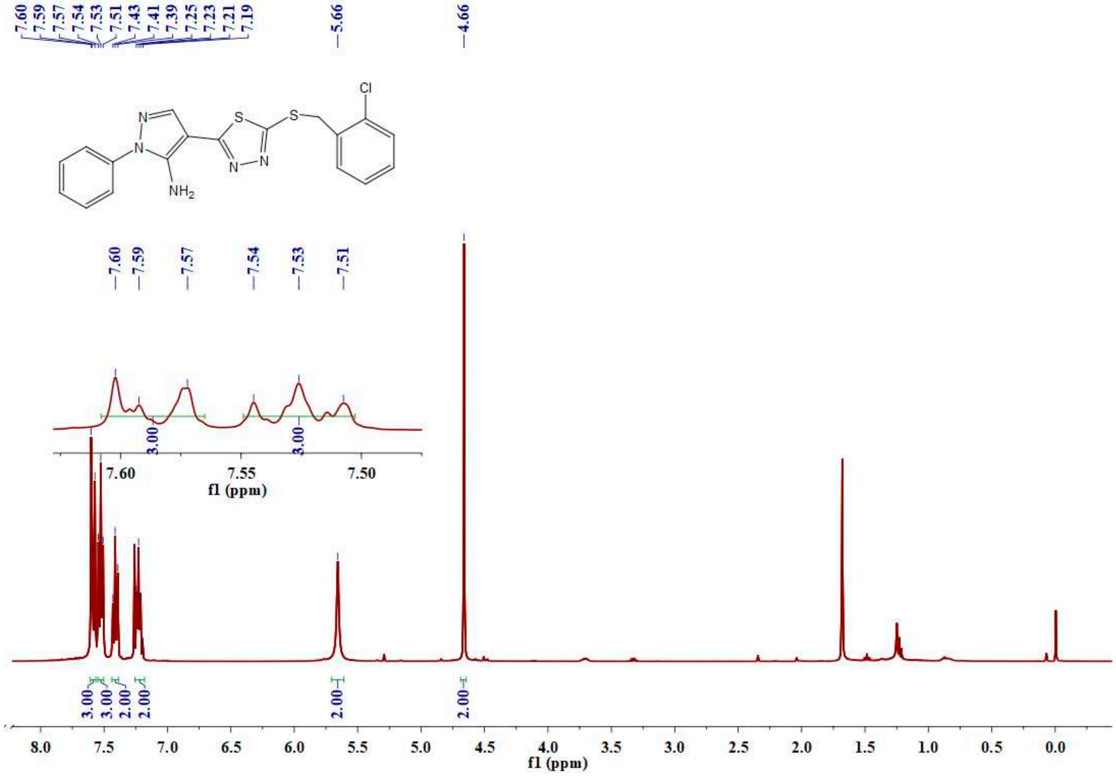

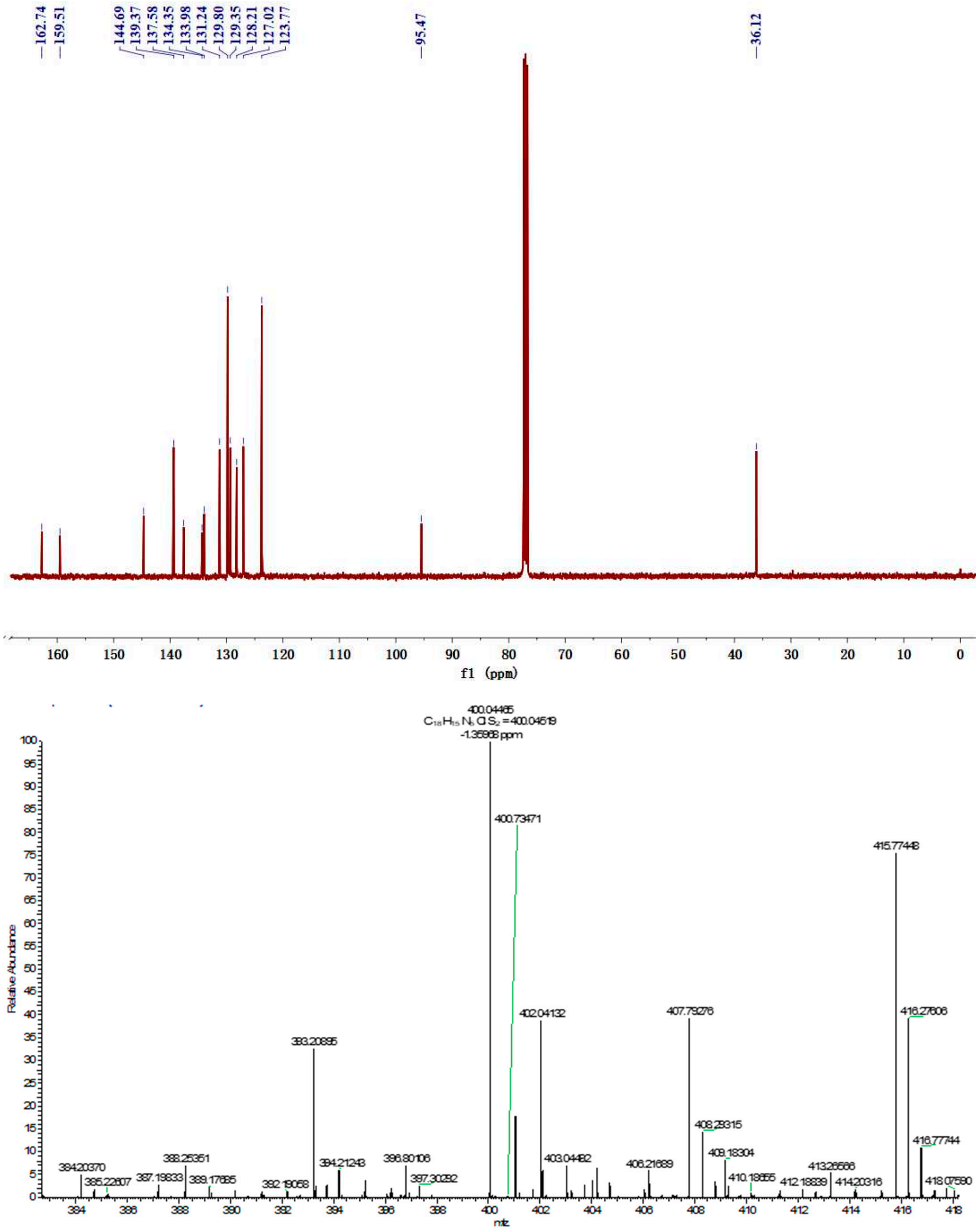

Figure S7. ^{1}H NMR, ^{13}C NMR and HRMS for title compound **E$_2$**.

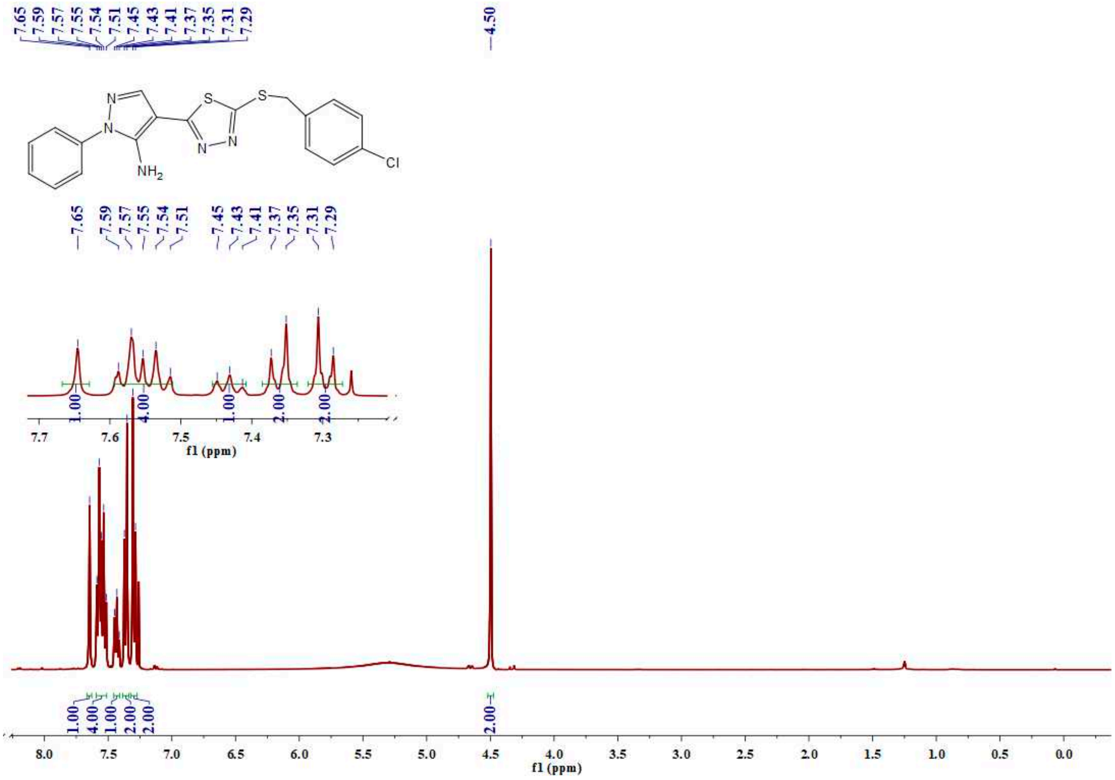
7.65 7.59 7.57 7.55 7.54 7.51 7.45 7.43 7.41 7.37 7.35 7.31 7.29
4.50
NH2
Cl
S
N
1.00 4.00 1.00 2.00 2.00
2.00
f1 (ppm)

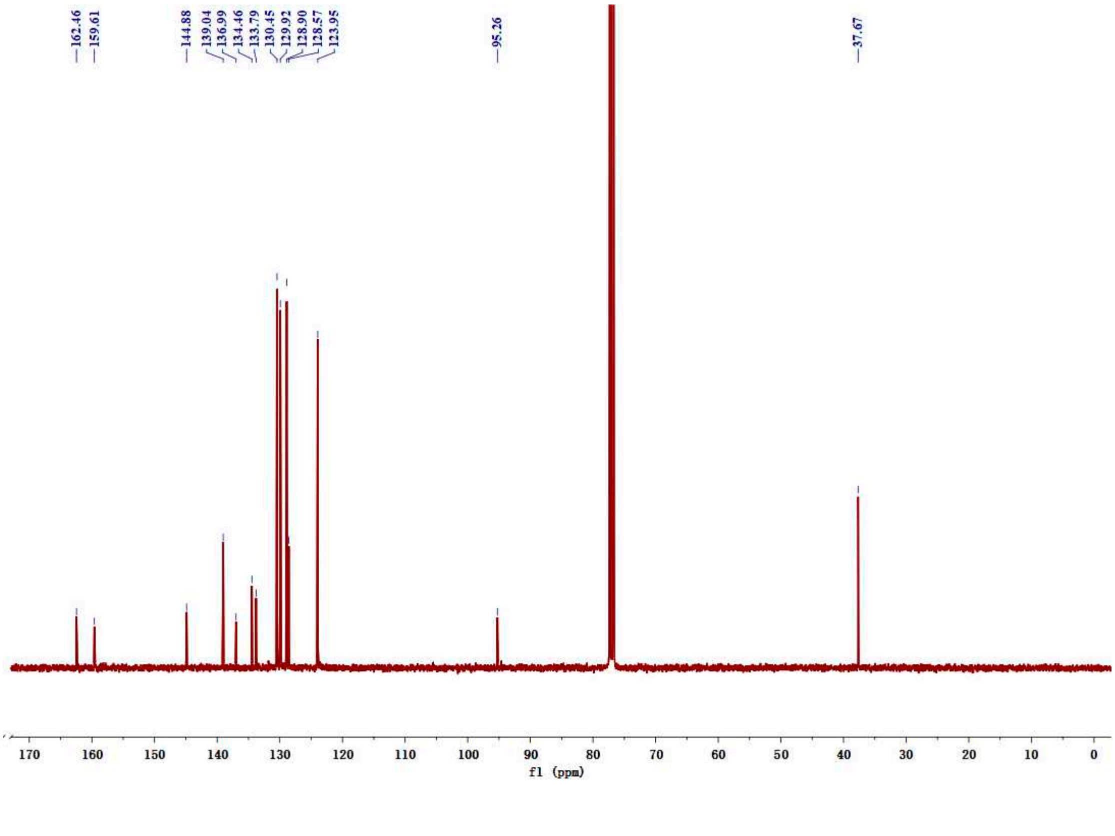
162.46 159.61
144.88 139.04 136.99 134.46 133.79 130.45 129.92 128.90 128.57 123.95
95.26
37.67
f1 (ppm)

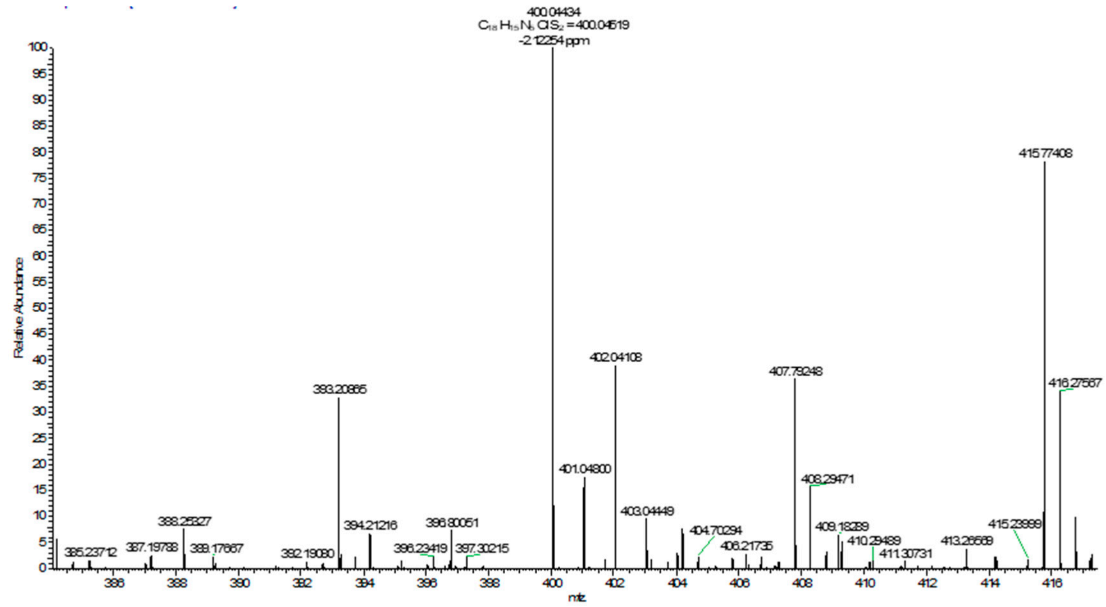

Figure S8. ^{1}H NMR, ^{13}C NMR and HRMS for title compound **E**$_3$.

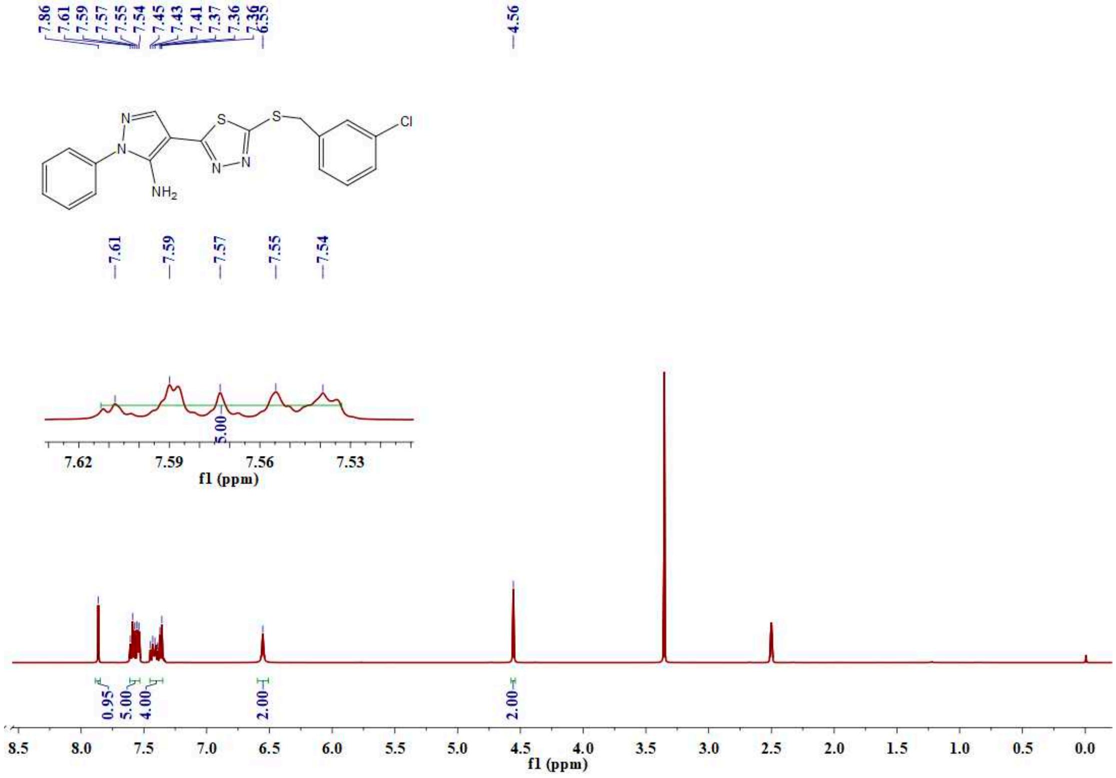

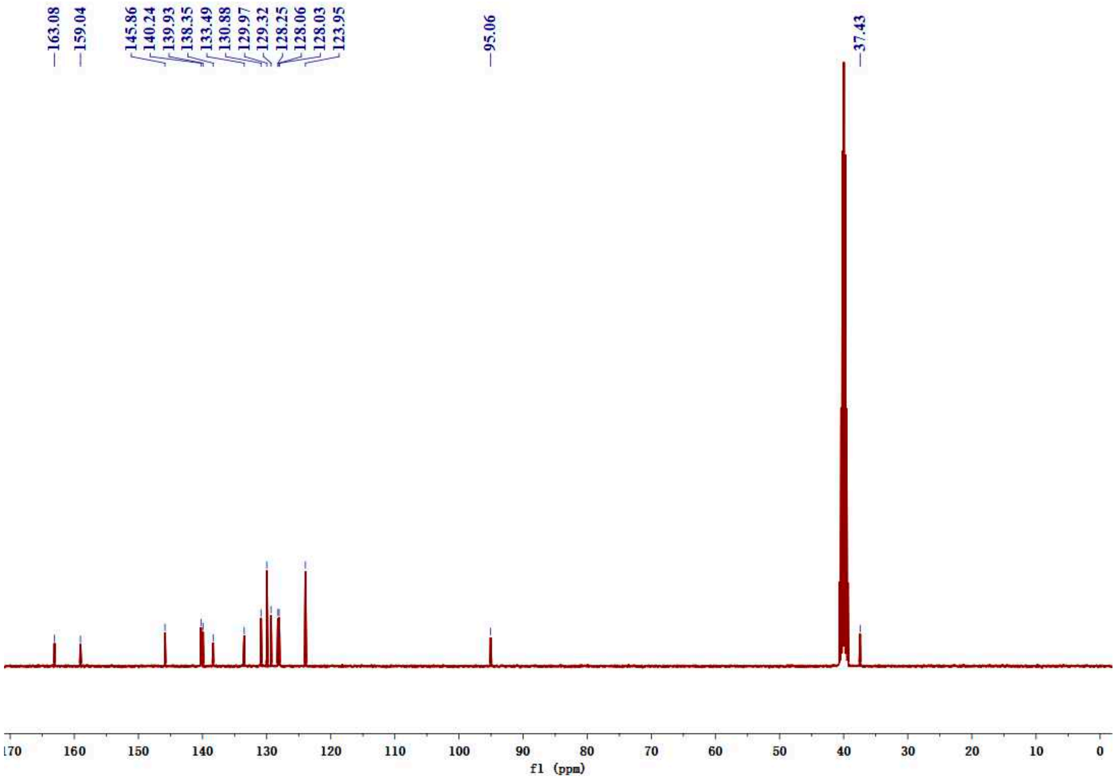

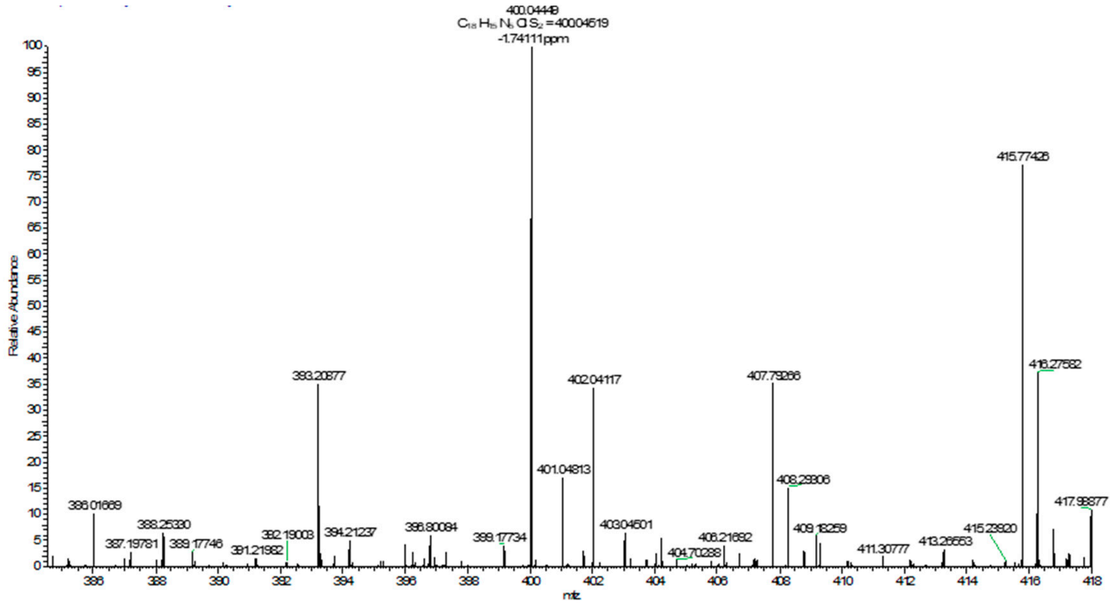

Figure S9. ^{1}H NMR, ^{13}C NMR and HRMS for title compound **E$_4$**.

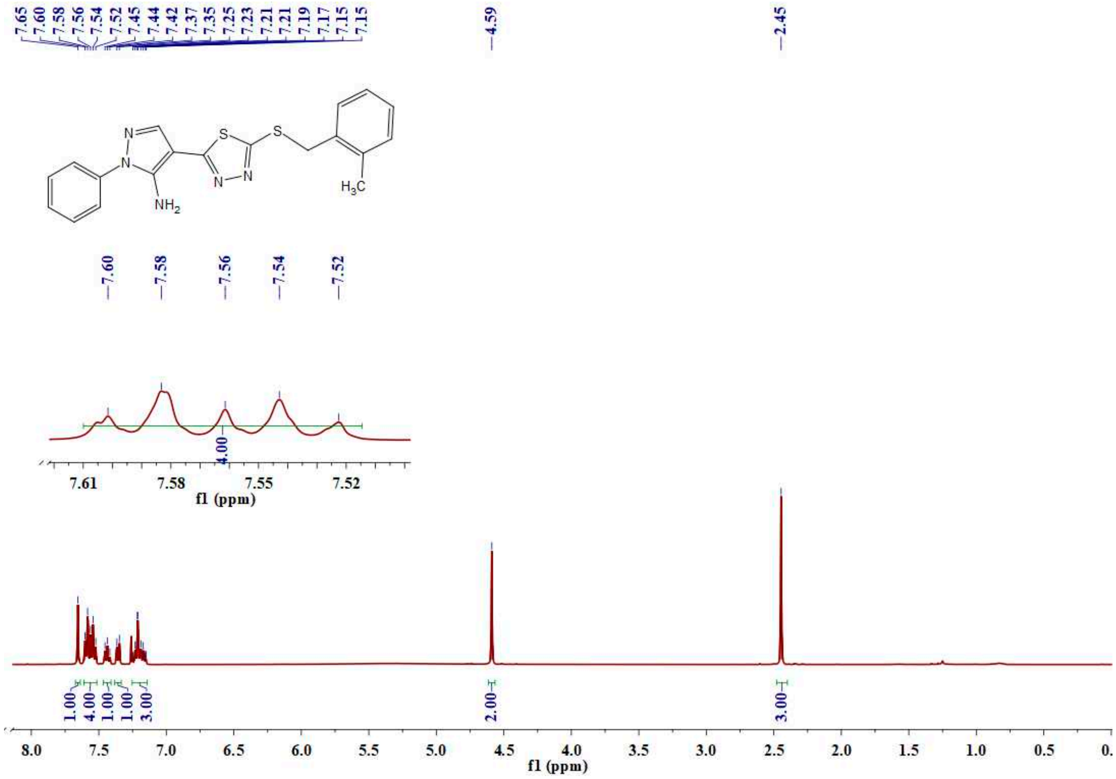
7.65 7.60 7.58 7.56 7.54 7.52 7.45 7.44 7.42 7.37 7.35 7.25 7.23 7.21 7.21 7.19 7.17 7.15 7.15
4.59
2.45
N
N
S
S
N
N
NH2
H3C
7.60 7.58 7.56 7.54 7.52
4.00
7.61 7.58 7.55 7.52
f1 (ppm)
1.00 4.00 1.00 1.00 3.00
2.00
3.00
8.0 7.5 7.0 6.5 6.0 5.5 5.0 4.5 4.0 3.5 3.0 2.5 2.0 1.5 1.0 0.5 0.
f1 (ppm)

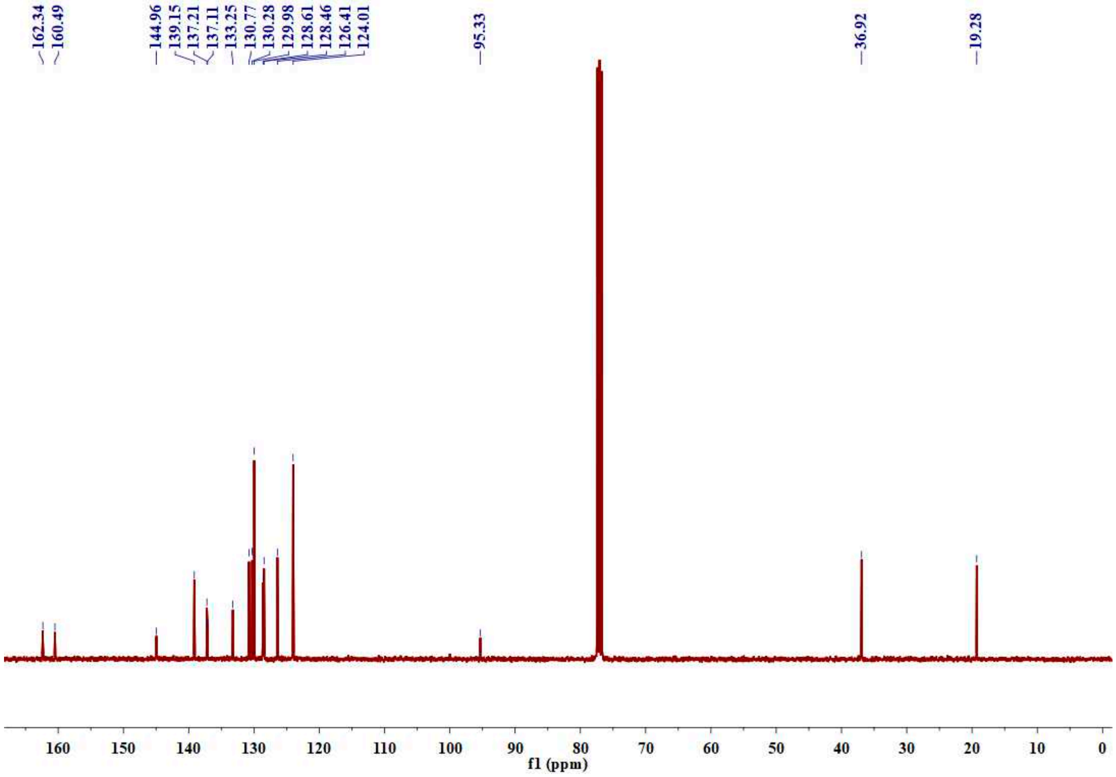
162.34 160.49
144.96 139.15 137.21 137.11 133.25 130.77 130.28 129.98 128.61 128.46 126.41 124.01
95.33
36.92
19.28
160 150 140 130 120 110 100 90 80 70 60 50 40 30 20 10 0
f1 (ppm)

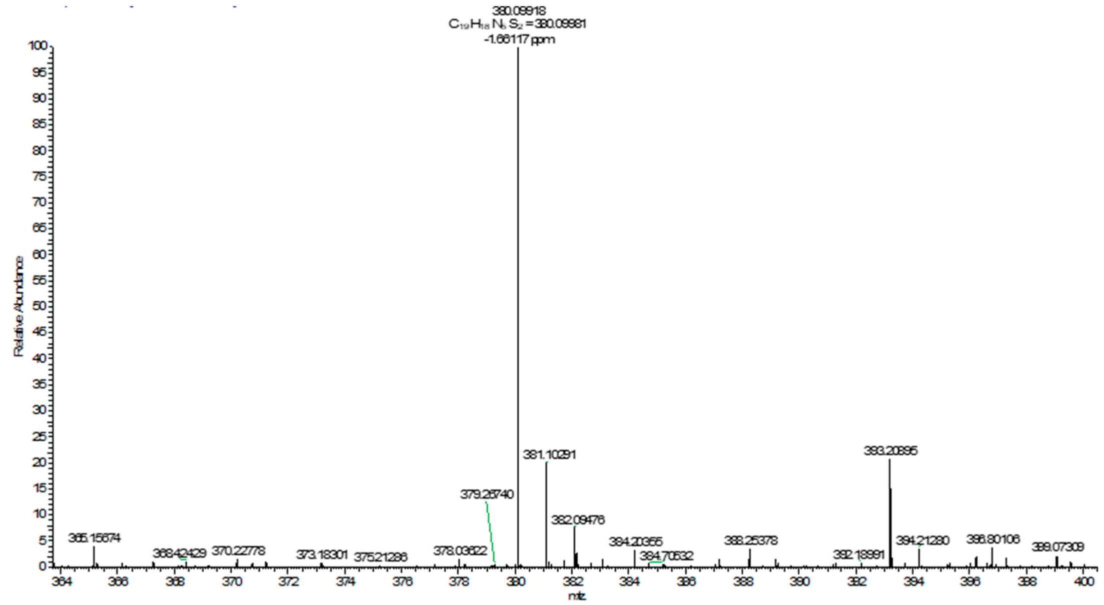

Figure S10. ^{1}H NMR, ^{13}C NMR and HRMS for title compound **E5**.

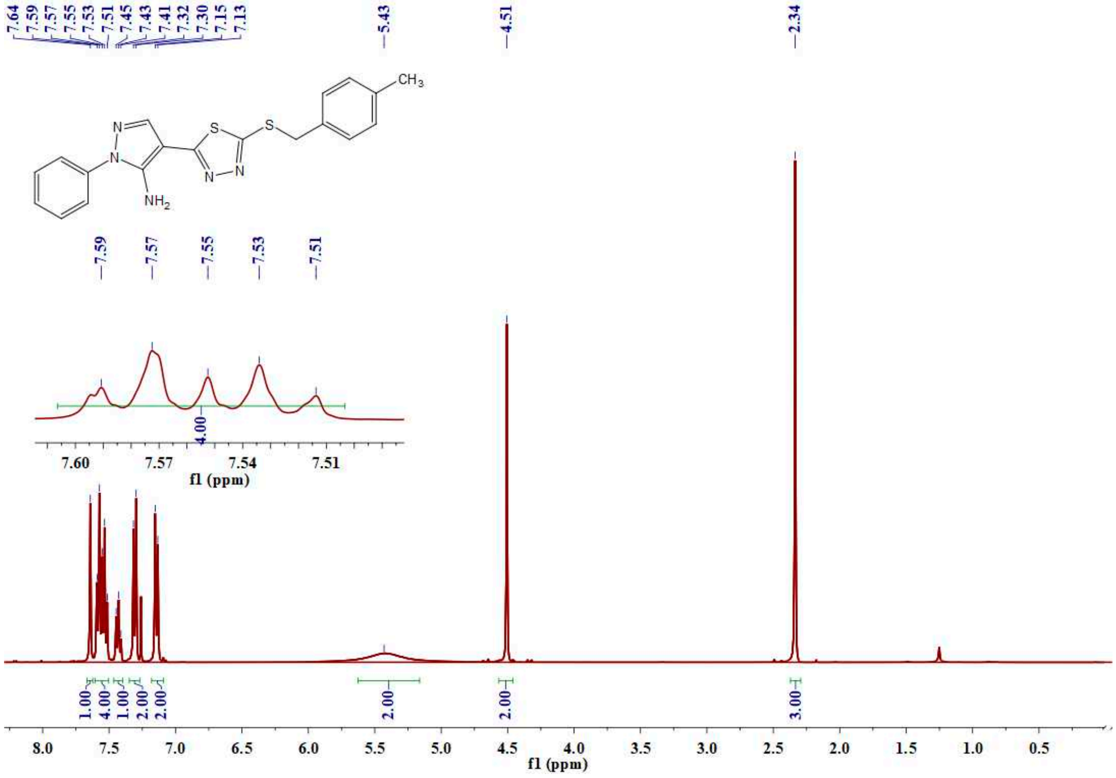

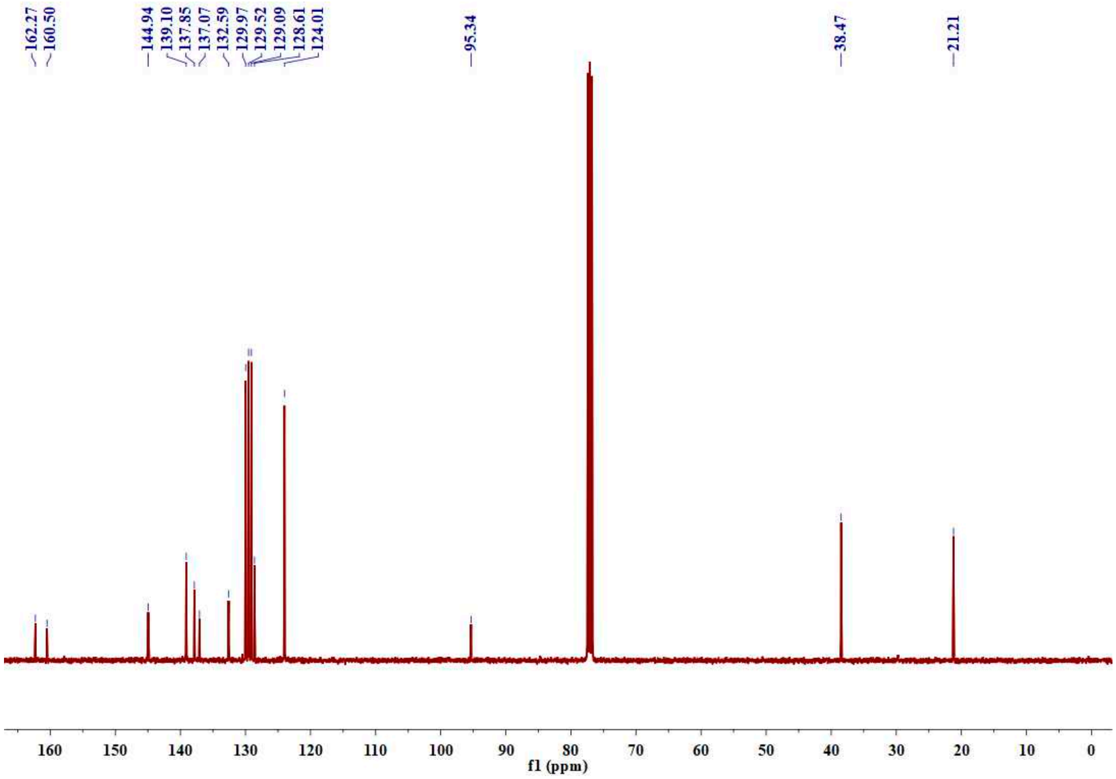

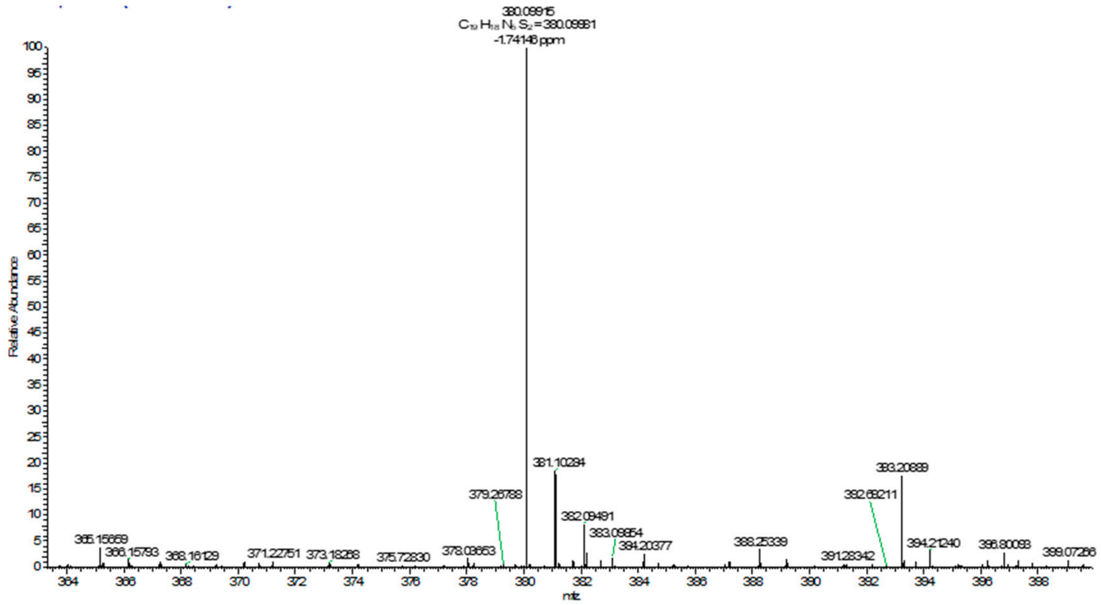

Figure S11. ^{1}H NMR, ^{13}C NMR and HRMS for title compound **E₆**.

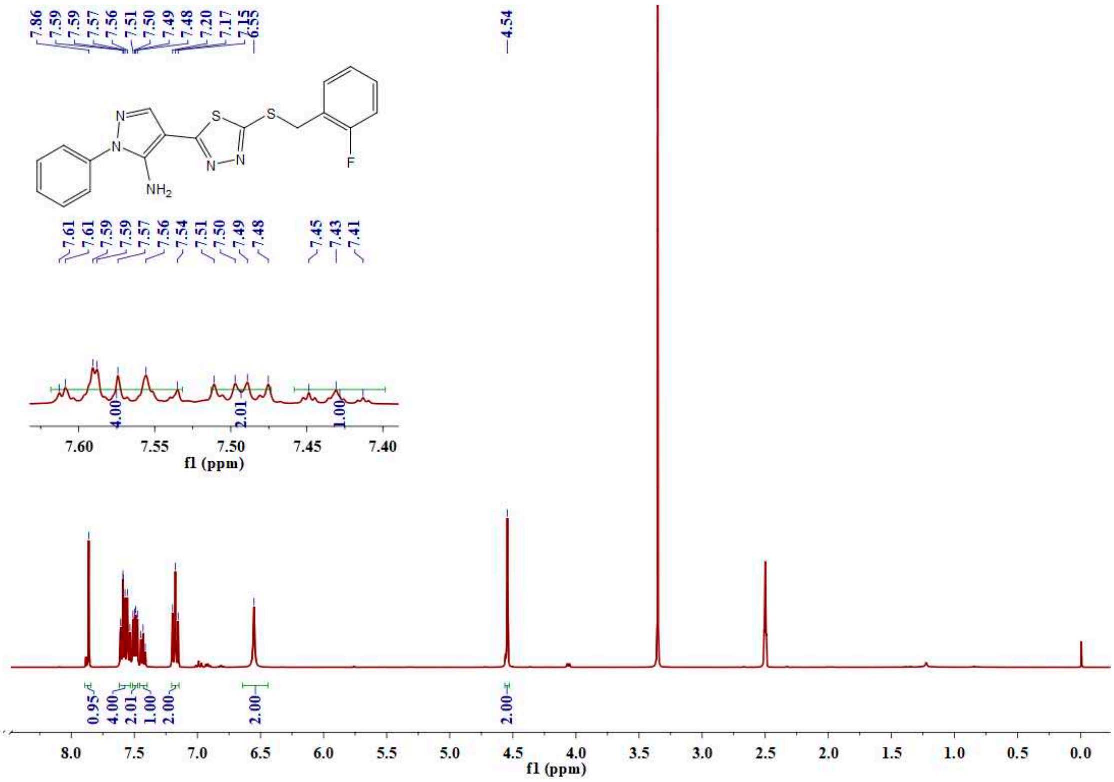
7.86 7.59 7.59 7.57 7.56 7.51 7.50 7.49 7.48 7.20 7.17 7.15 6.55
4.54
S
N
N
N
N
S
F
NH2
7.61 7.61 7.59 7.59 7.57 7.56 7.54 7.51 7.50 7.49 7.48 7.45 7.43 7.41
4.00 2.01 1.00
7.60 7.55 7.50 7.45 7.40
f1 (ppm)
0.95 4.00 2.01 1.00 2.00 2.00 2.00
8.0 7.5 7.0 6.5 6.0 5.5 5.0 4.5 4.0 3.5 3.0 2.5 2.0 1.5 1.0 0.5 0.0
f1 (ppm)

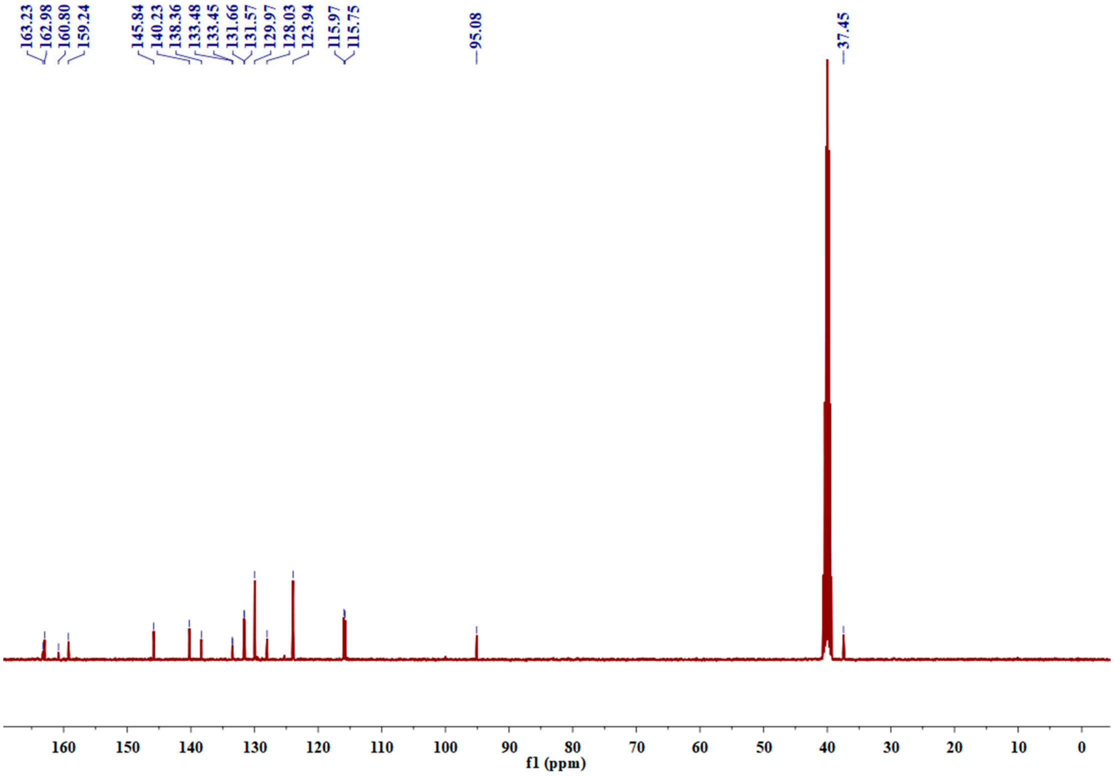
163.23 162.98 160.80 159.24 145.84 140.23 138.36 133.48 133.45 131.66 131.57 129.97 128.03 123.94 115.97 115.75
95.08
37.45
160 150 140 130 120 110 100 90 80 70 60 50 40 30 20 10 0
f1 (ppm)

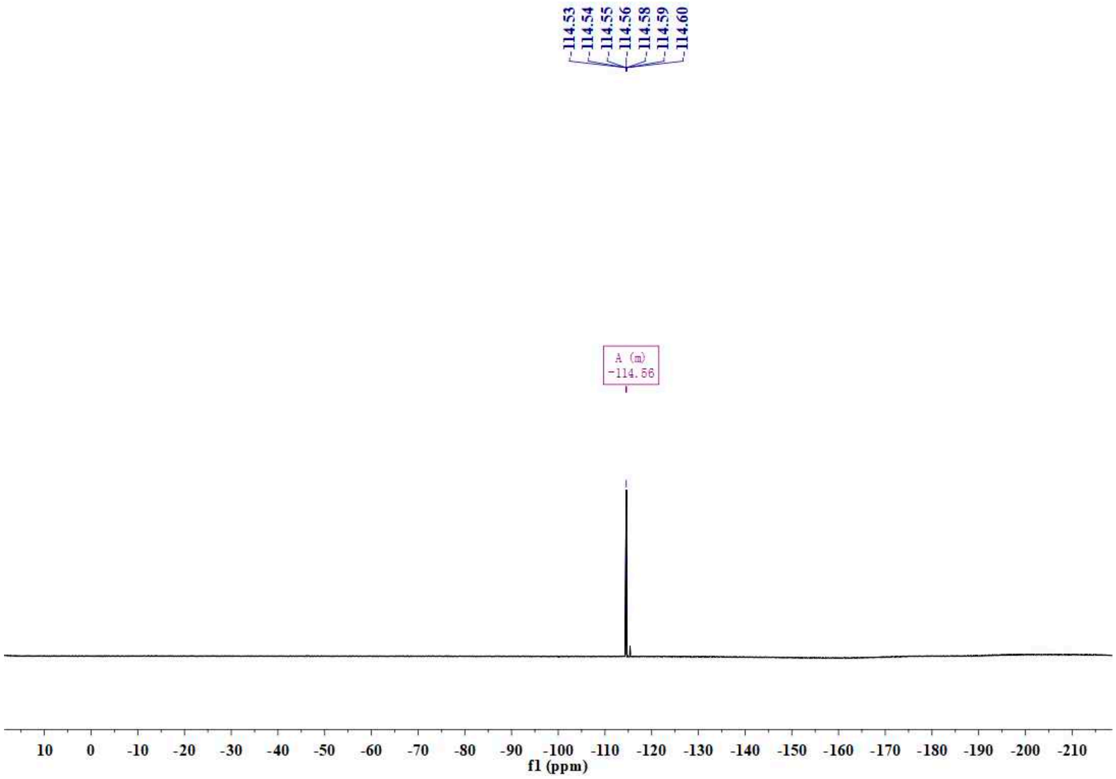

-114.53
-114.54
-114.55
-114.56
-114.58
-114.59
-114.60
A (m)
-114.56
10 0 -10 -20 -30 -40 -50 -60 -70 -80 -90 -100 -110 -120 -130 -140 -150 -160 -170 -180 -190 -200 -210
f1 (ppm)

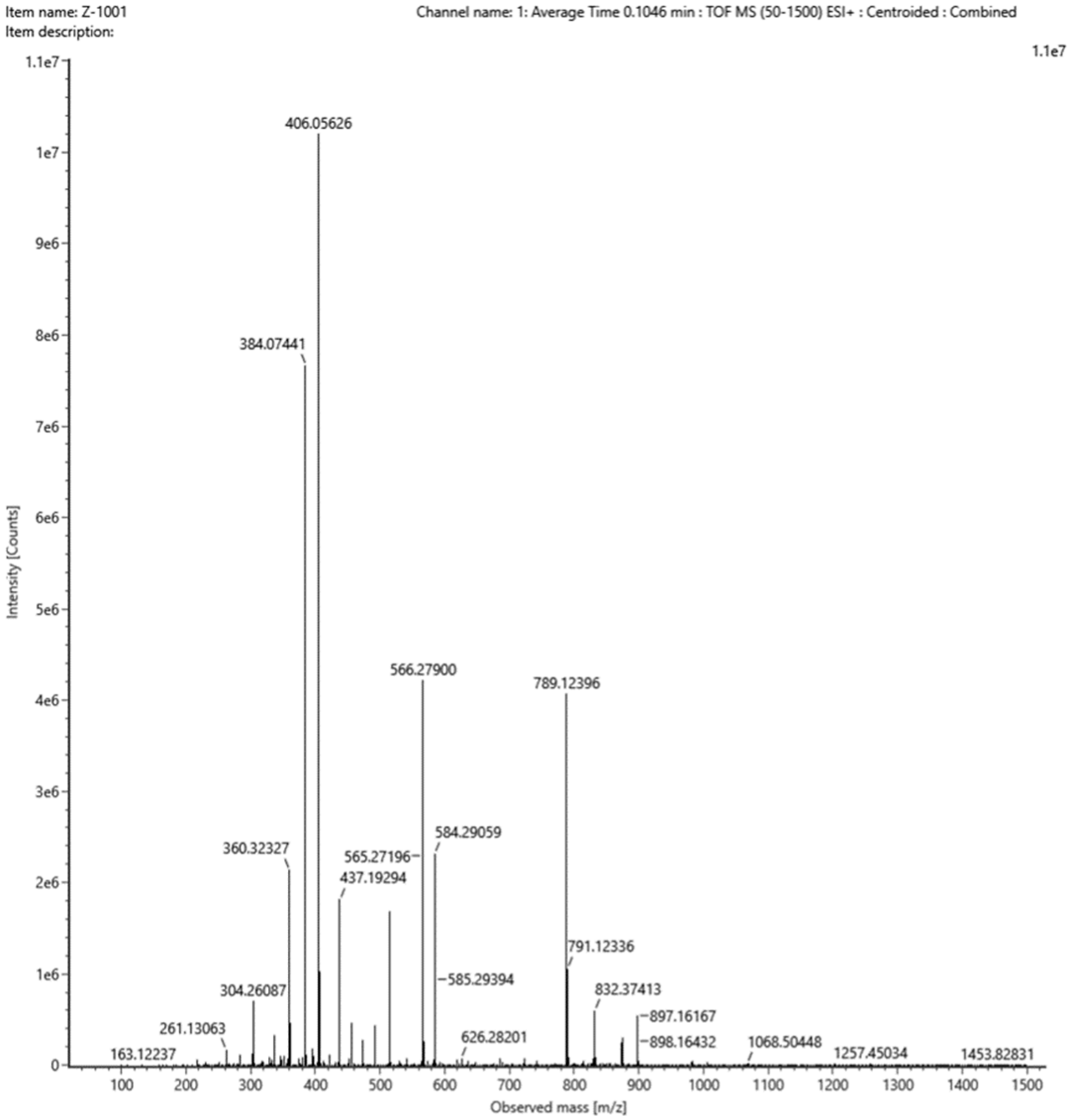

Figure S12. ^{1}H NMR, ^{13}C NMR, ^{19}F NMR and HRMS for title compound **E**$_7$.

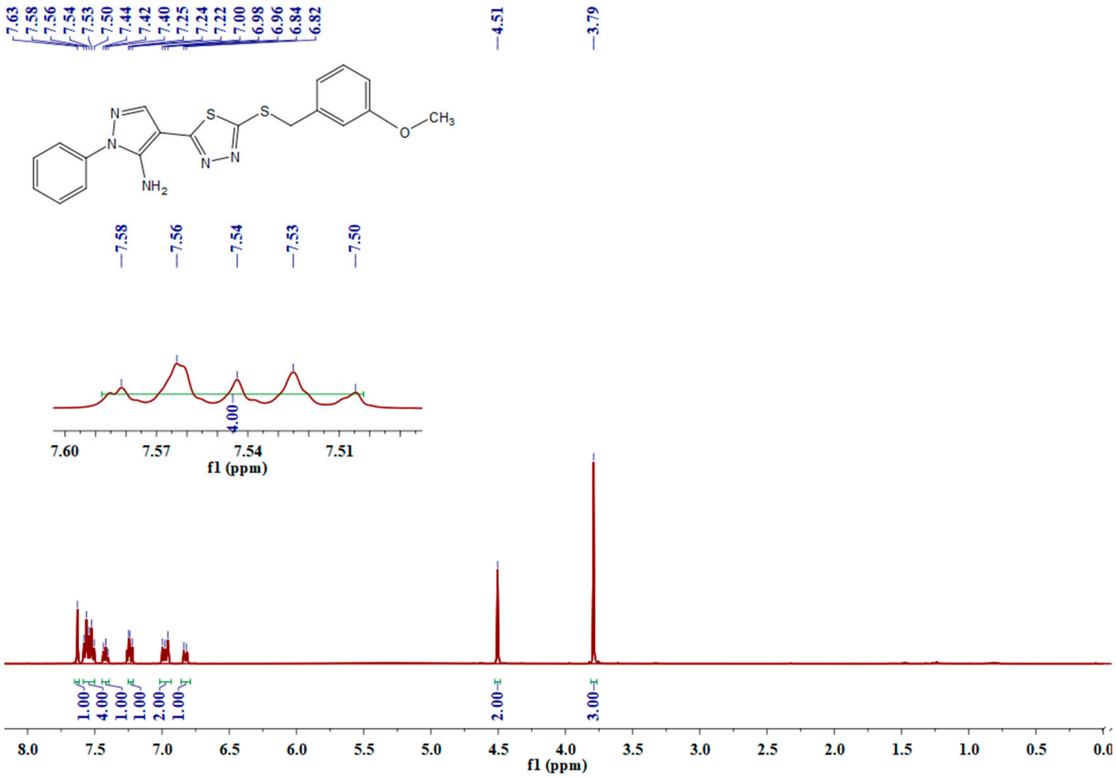
7.63 7.58 7.56 7.54 7.53 7.50 7.44 7.42 7.40 7.25 7.24 7.22 7.00 6.98 6.96 6.84 6.82
4.51
3.79
N
N
S
S
N
N
O
CH3
NH2
7.58
7.56
7.54
7.53
7.50
4.00
7.60 7.57 7.54 7.51
f1 (ppm)
1.00 4.00 1.00 1.00 2.00 1.00
2.00
3.00
8.0 7.5 7.0 6.5 6.0 5.5 5.0 4.5 4.0 3.5 3.0 2.5 2.0 1.5 1.0 0.5 0.0
f1 (ppm)

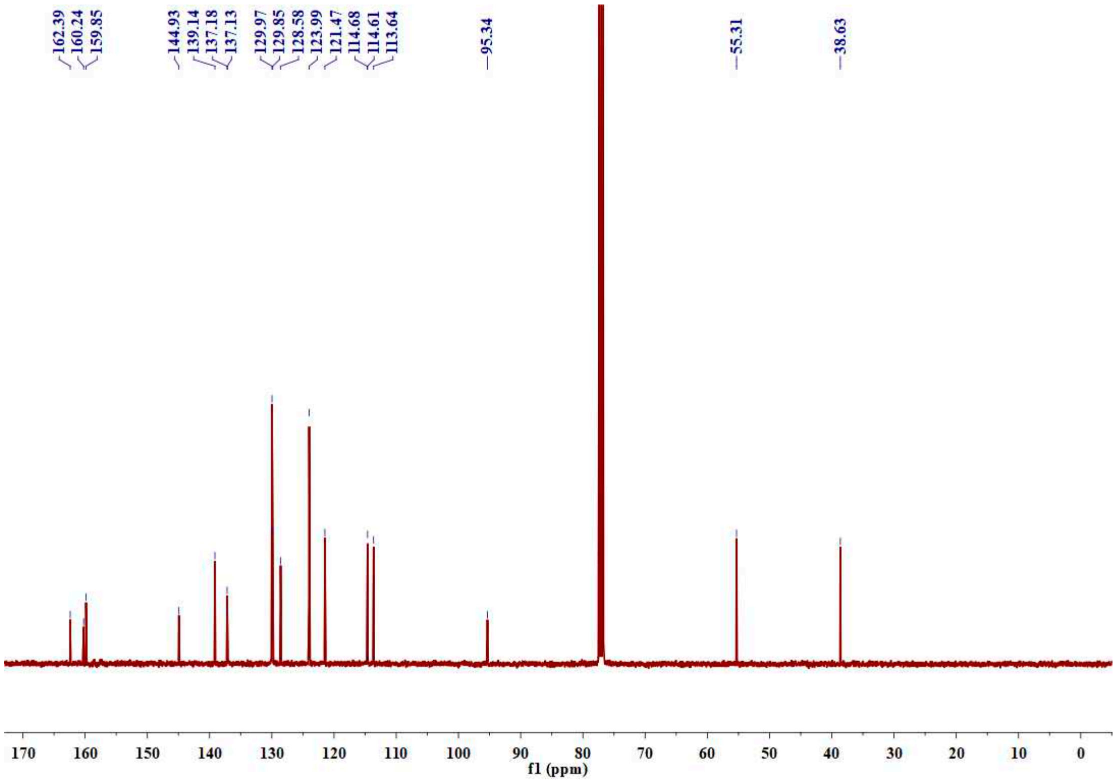
162.39 160.24 159.85
144.93 139.14 137.18 137.13 129.97 129.85 128.58 123.99 121.47 114.68 114.61 113.64
95.34
55.31
38.63
170 160 150 140 130 120 110 100 90 80 70 60 50 40 30 20 10 0
f1 (ppm)

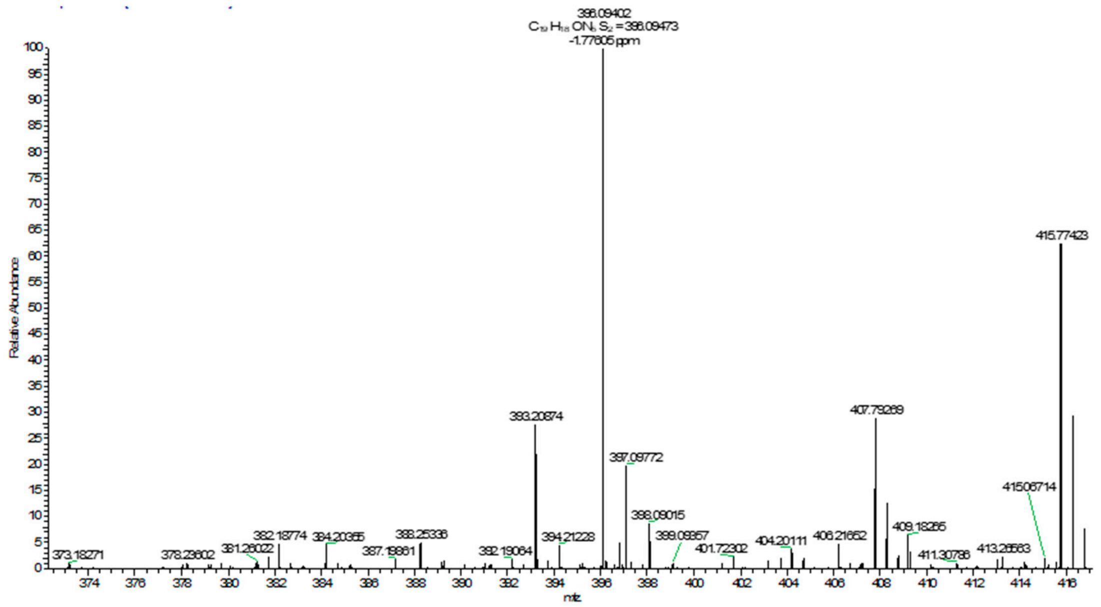

Figure S13. ^{1}H NMR, ^{13}C NMR and HRMS for title compound **E₈**.

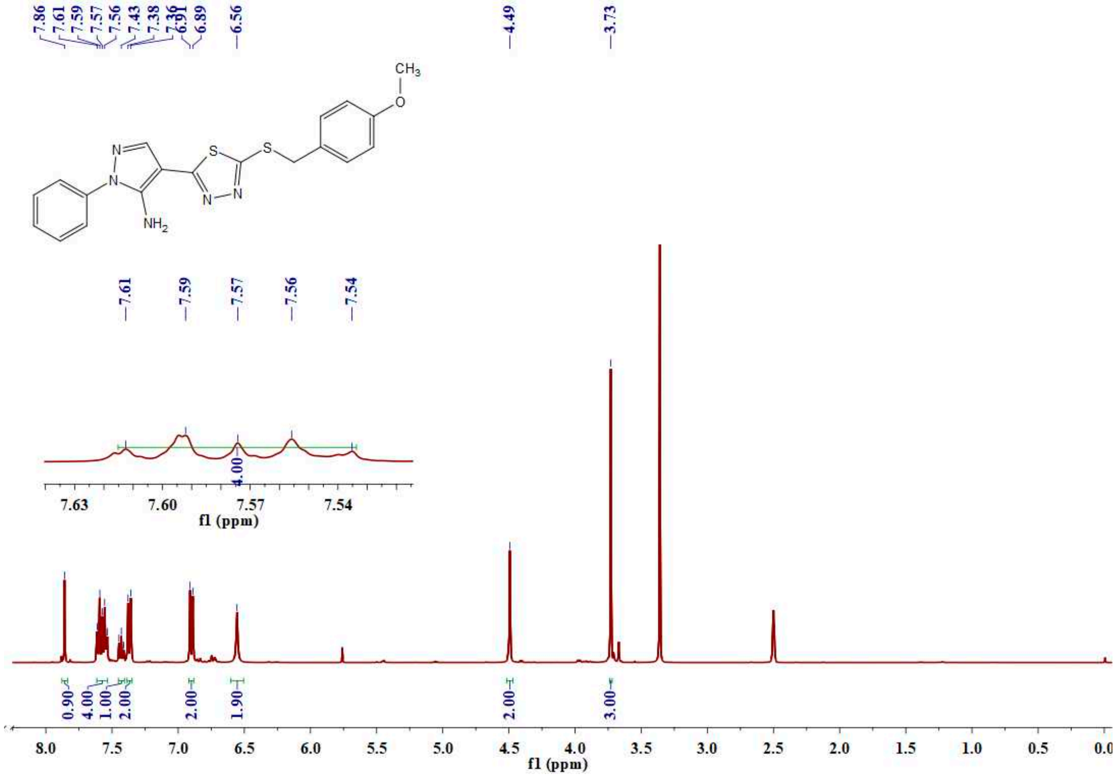

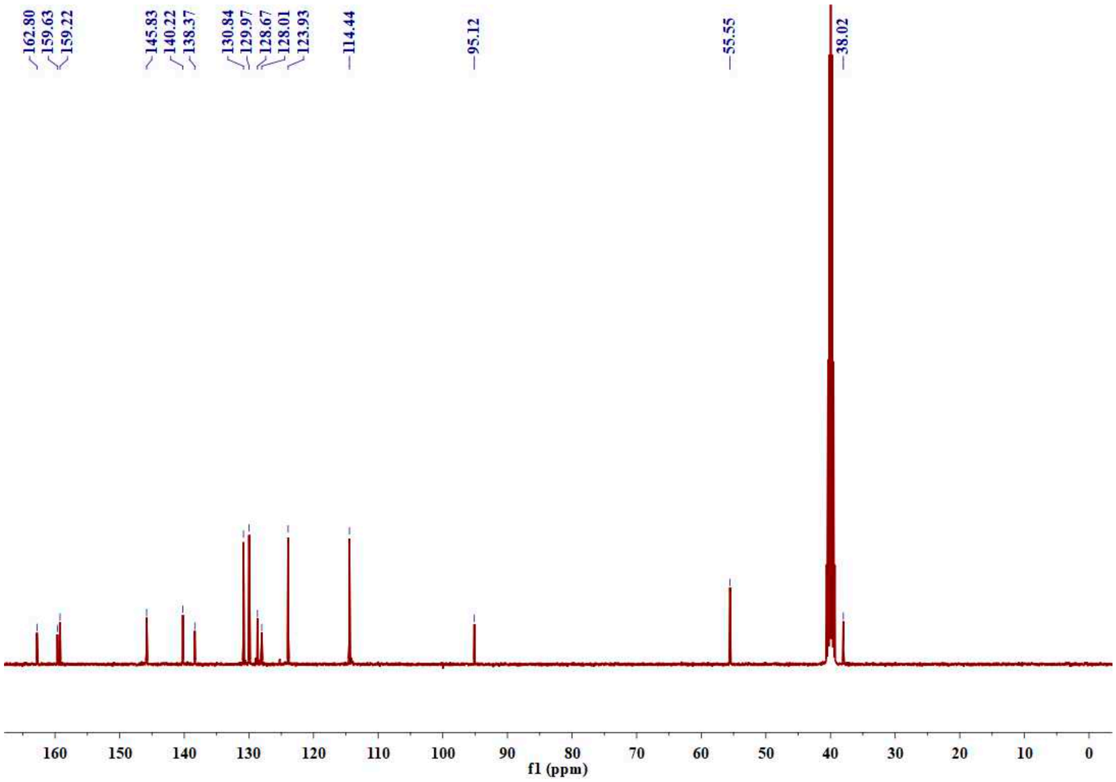

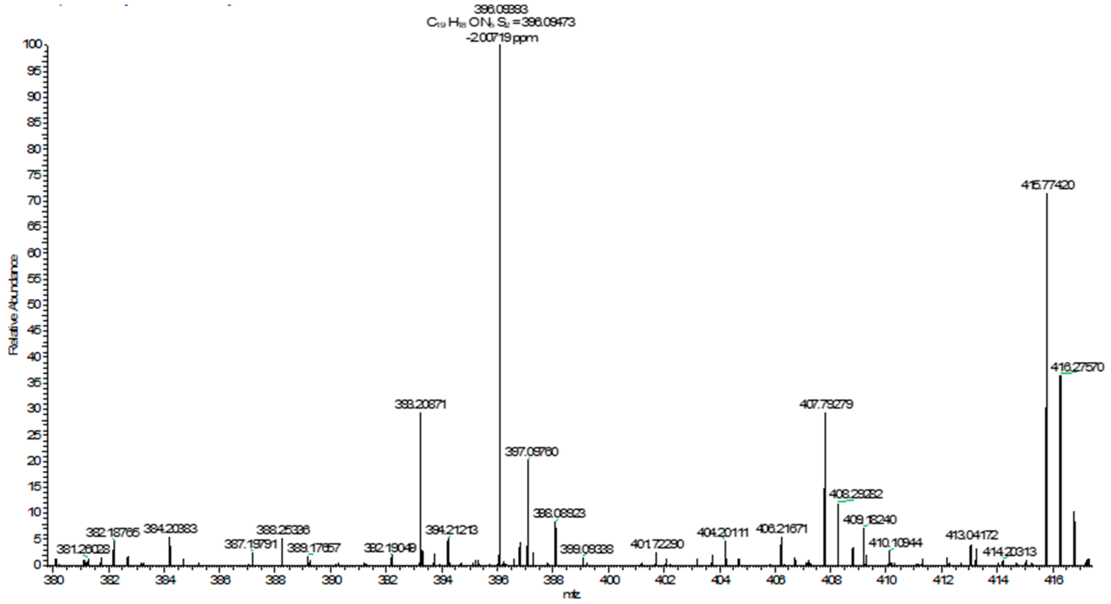

Figure S14. ^{1}H NMR, ^{13}C NMR and HRMS for title compound **E₉**.

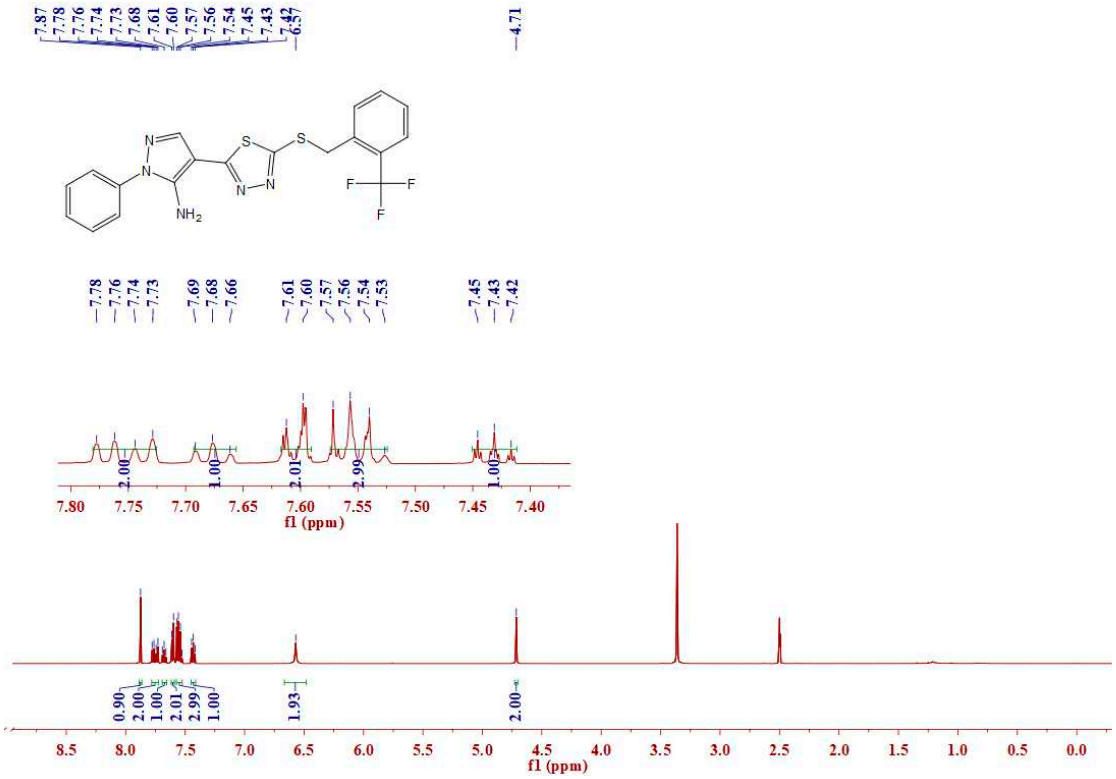
7.87 7.78 7.76 7.74 7.73 7.68 7.61 7.60 7.57 7.56 7.54 7.45 7.43 7.42 6.57
4.71
N
N
S
S
N
N
NH2
F
F
F
7.78 7.76 7.74 7.73 7.69 7.68 7.66 7.61 7.60 7.57 7.56 7.54 7.53 7.45 7.43 7.42
2.00 1.00 2.01 2.99 1.00
7.80 7.75 7.70 7.65 7.60 7.55 7.50 7.45 7.40
f1 (ppm)
0.90 2.00 1.00 2.01 2.99 1.00 1.93 2.00
8.5 8.0 7.5 7.0 6.5 6.0 5.5 5.0 4.5 4.0 3.5 3.0 2.5 2.0 1.5 1.0 0.5 0.0
f1 (ppm)

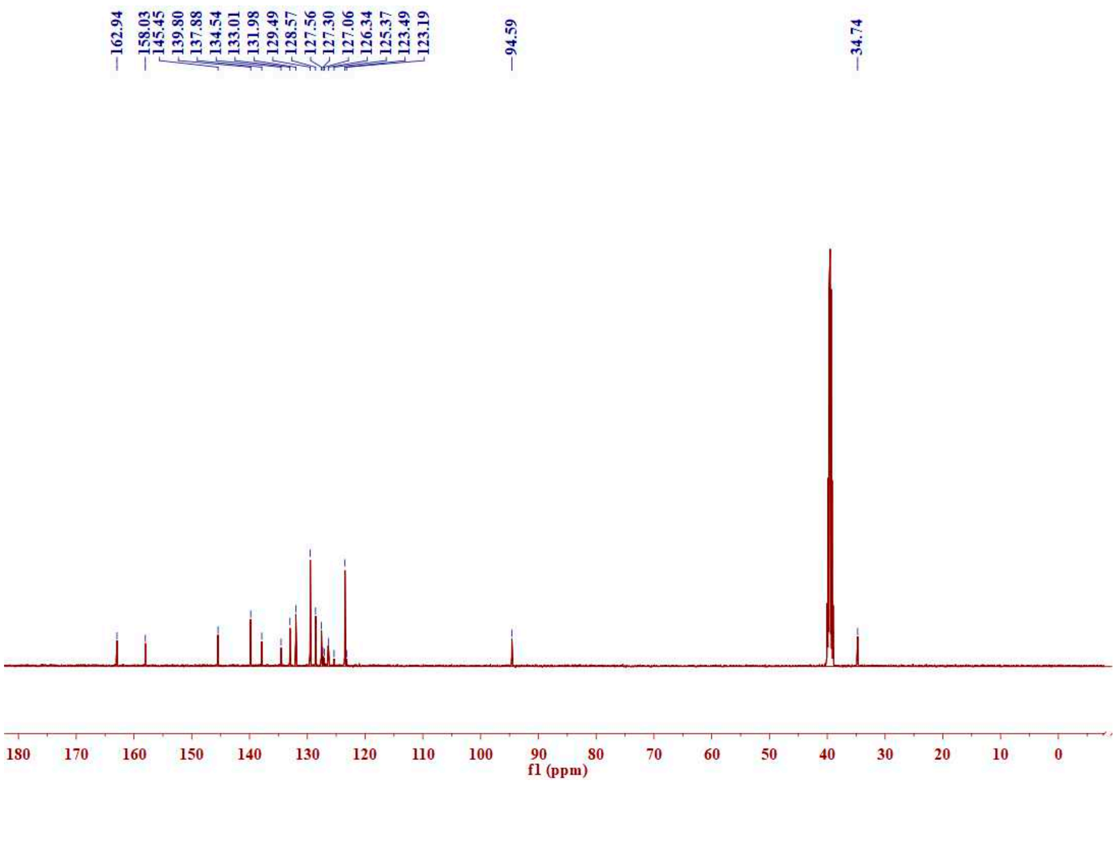
162.94 158.03 145.45 139.80 137.88 134.54 133.01 131.98 129.49 128.57 127.56 127.30 127.06 126.34 125.37 123.49 123.19
94.59
34.74
180 170 160 150 140 130 120 110 100 90 80 70 60 50 40 30 20 10 0
f1 (ppm)

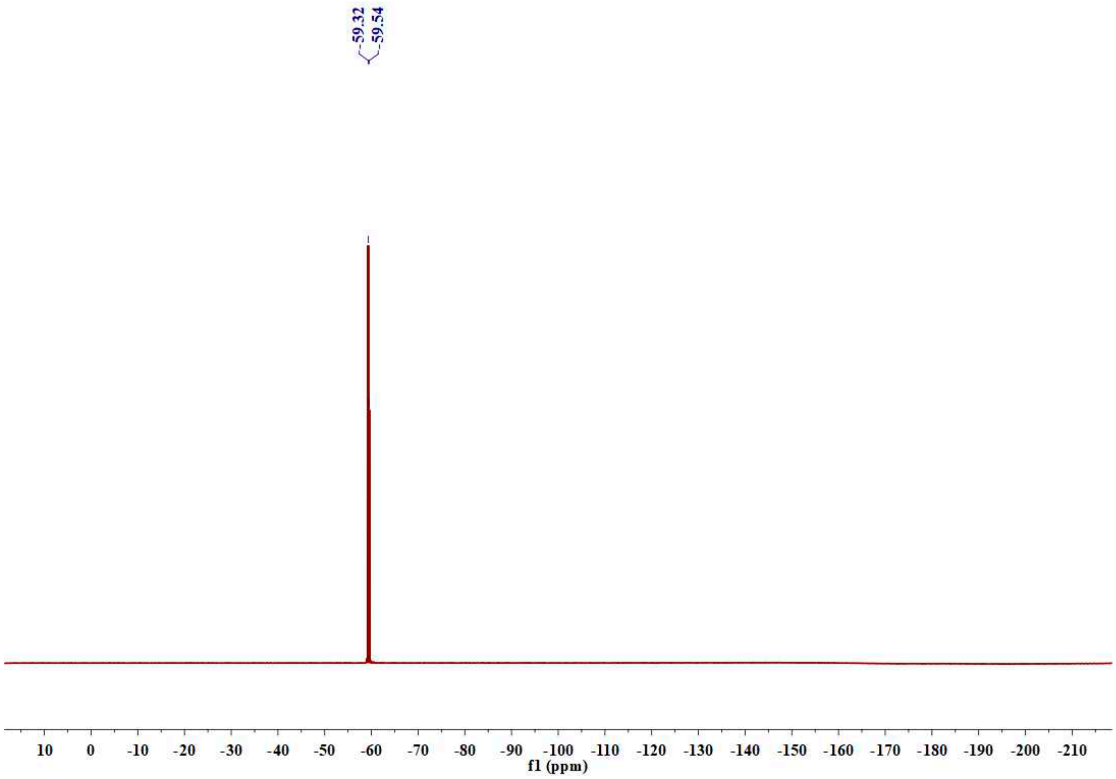

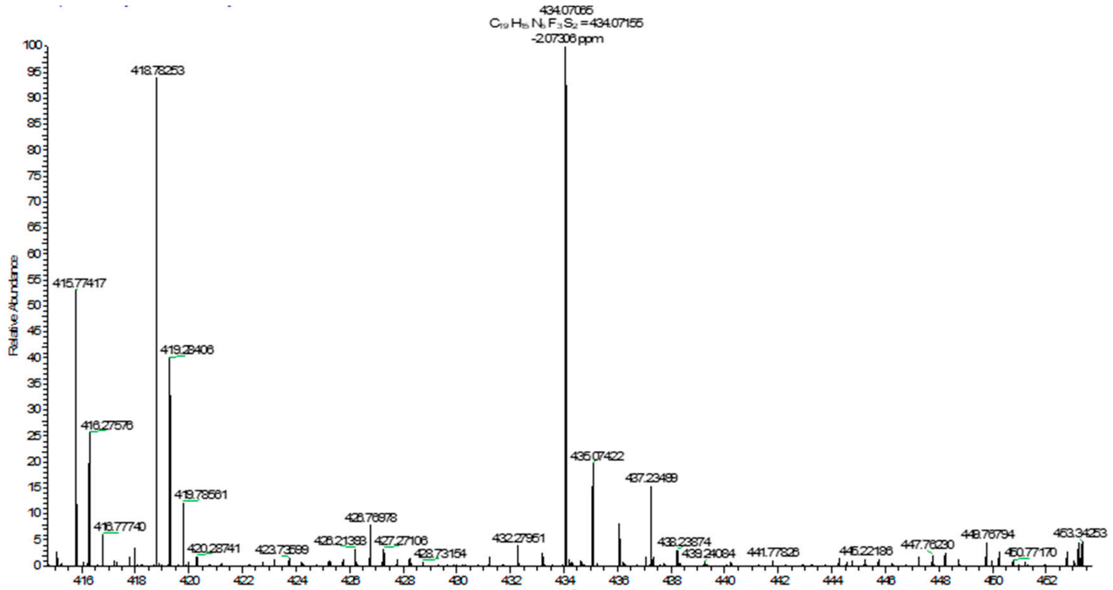

Figure S15. ^{1}H NMR, ^{13}C NMR, ^{19}F NMR and HRMS for title compound **E_{10}**.

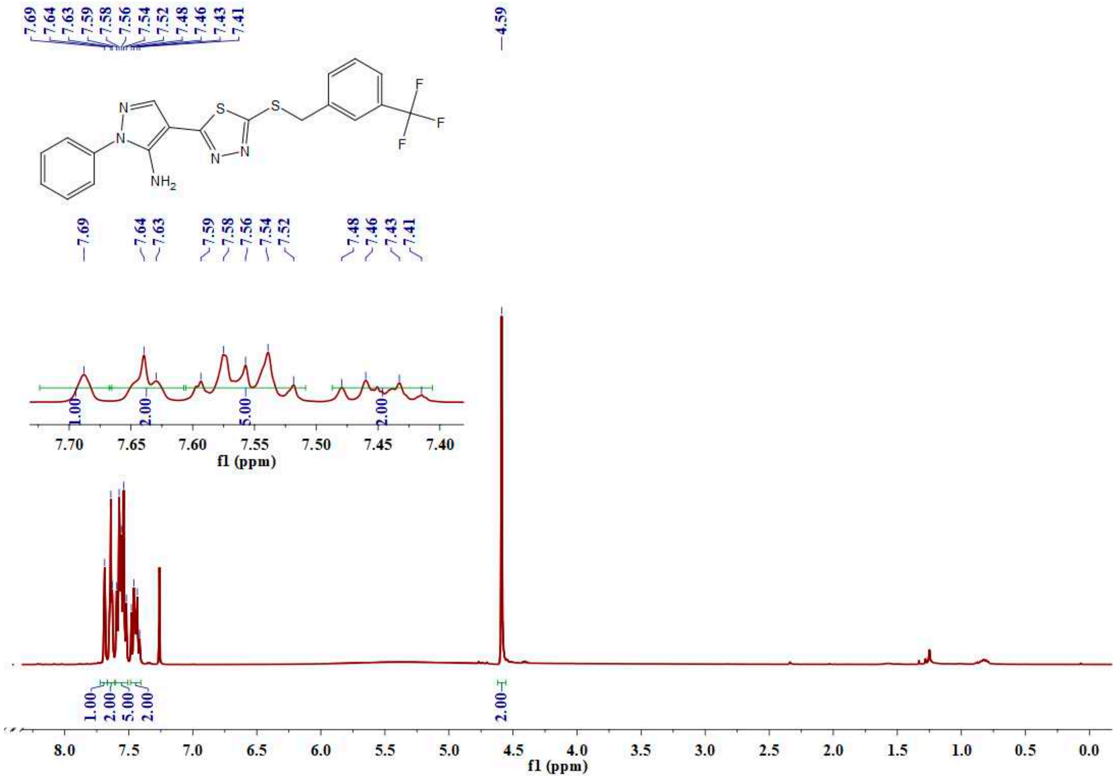
7.69
7.64
7.63
7.59
7.58
7.56
7.54
7.52
7.48
7.46
7.43
7.41
4.59
N
N
S
N
N
S
F
F
F
NH2
1.00
2.00
5.00
2.00
7.70 7.65 7.60 7.55 7.50 7.45 7.40
f1 (ppm)
8.0 7.5 7.0 6.5 6.0 5.5 5.0 4.5 4.0 3.5 3.0 2.5 2.0 1.5 1.0 0.5 0.0
f1 (ppm)

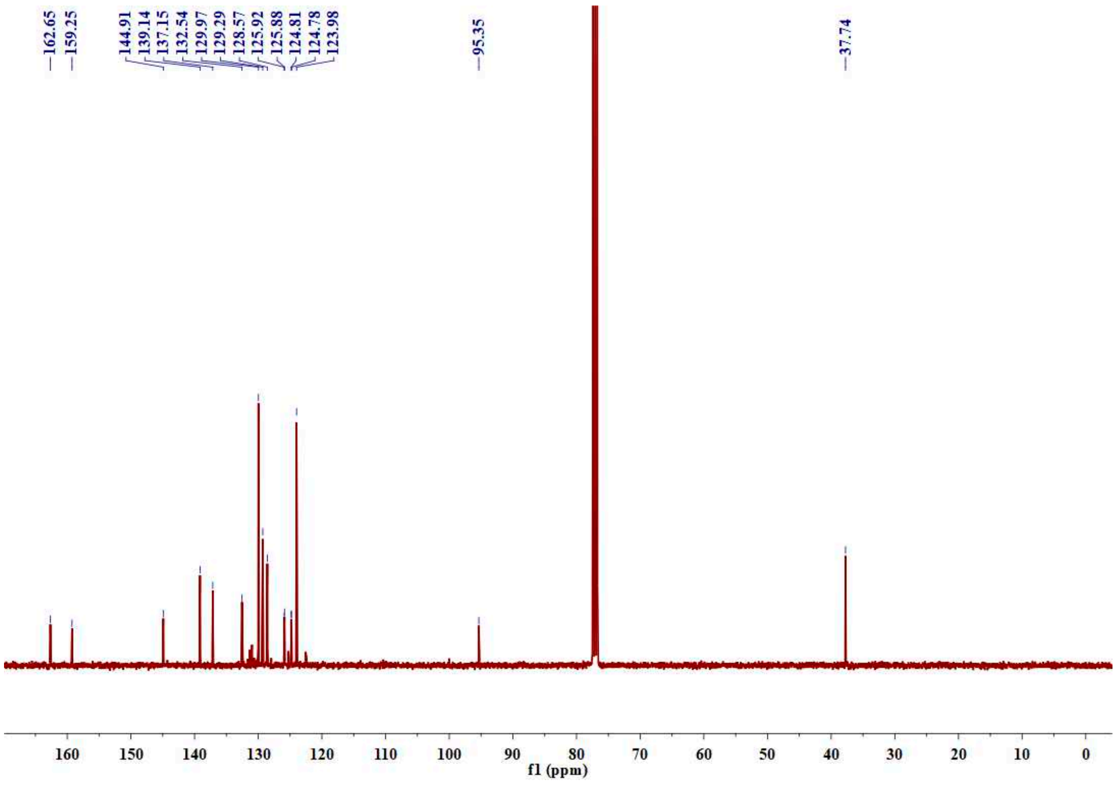
162.65
159.25
144.91
139.14
137.15
132.54
129.97
129.29
128.57
125.92
125.88
124.81
124.78
123.98
95.35
37.74
160 150 140 130 120 110 100 90 80 70 60 50 40 30 20 10 0
f1 (ppm)

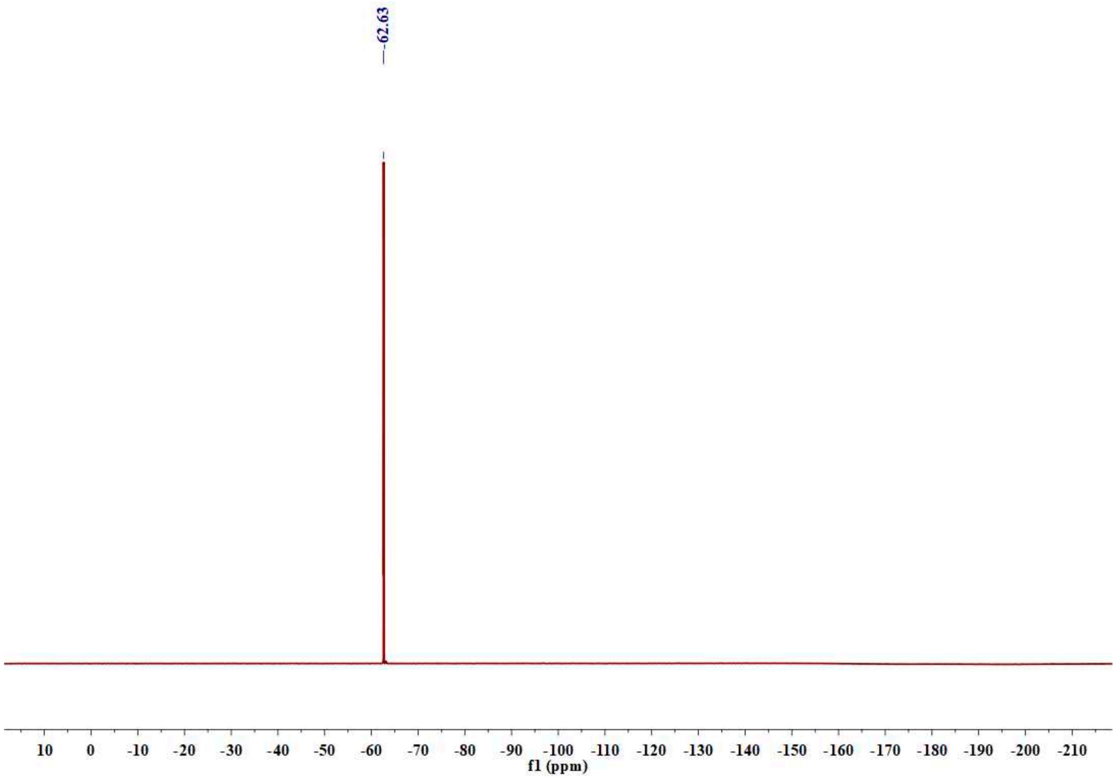

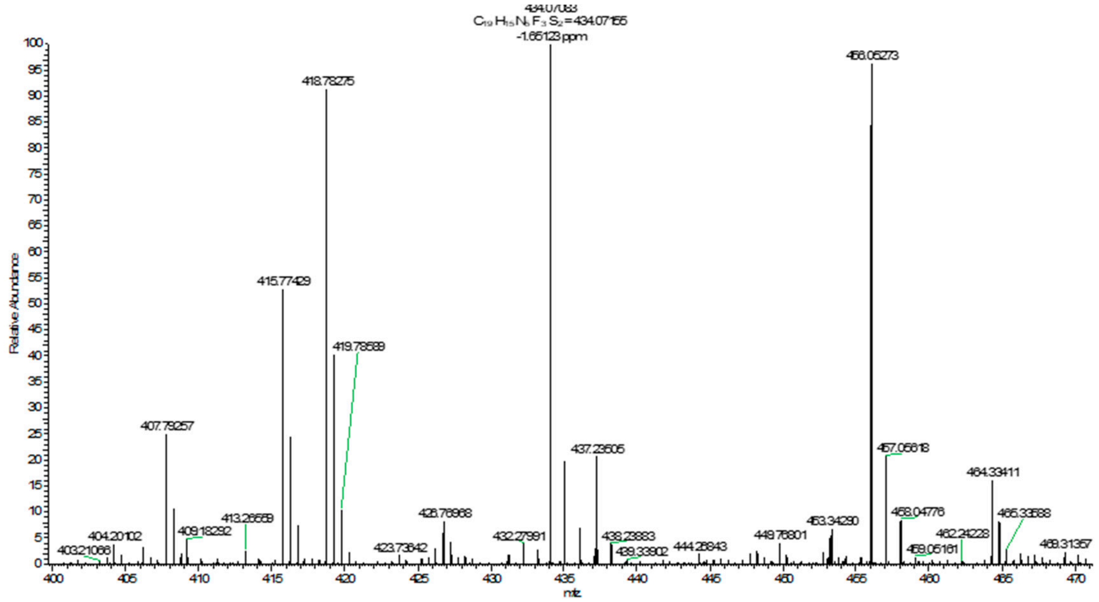

Figure S16. ^{1}H NMR, ^{13}C NMR, ^{19}F NMR and HRMS for title compound **E11**.

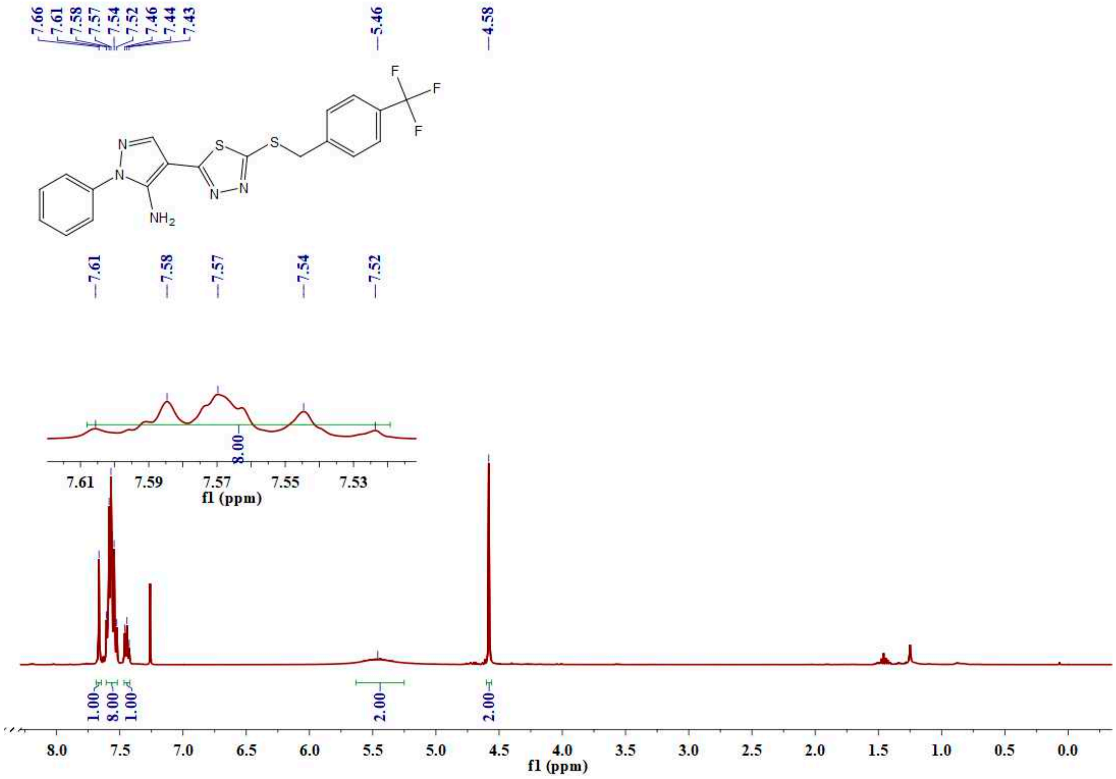
7.66 7.61 7.58 7.57 7.54 7.52 7.46 7.44 7.43
5.46
4.58
F
F
F
S
N
N
N
N
S
NH2
7.61 7.58 7.57 7.54 7.52
8.00
7.61 7.59 7.57 7.55 7.53
f1 (ppm)
1.00 8.00 1.00
2.00
2.00
8.0 7.5 7.0 6.5 6.0 5.5 5.0 4.5 4.0 3.5 3.0 2.5 2.0 1.5 1.0 0.5 0.0
f1 (ppm)

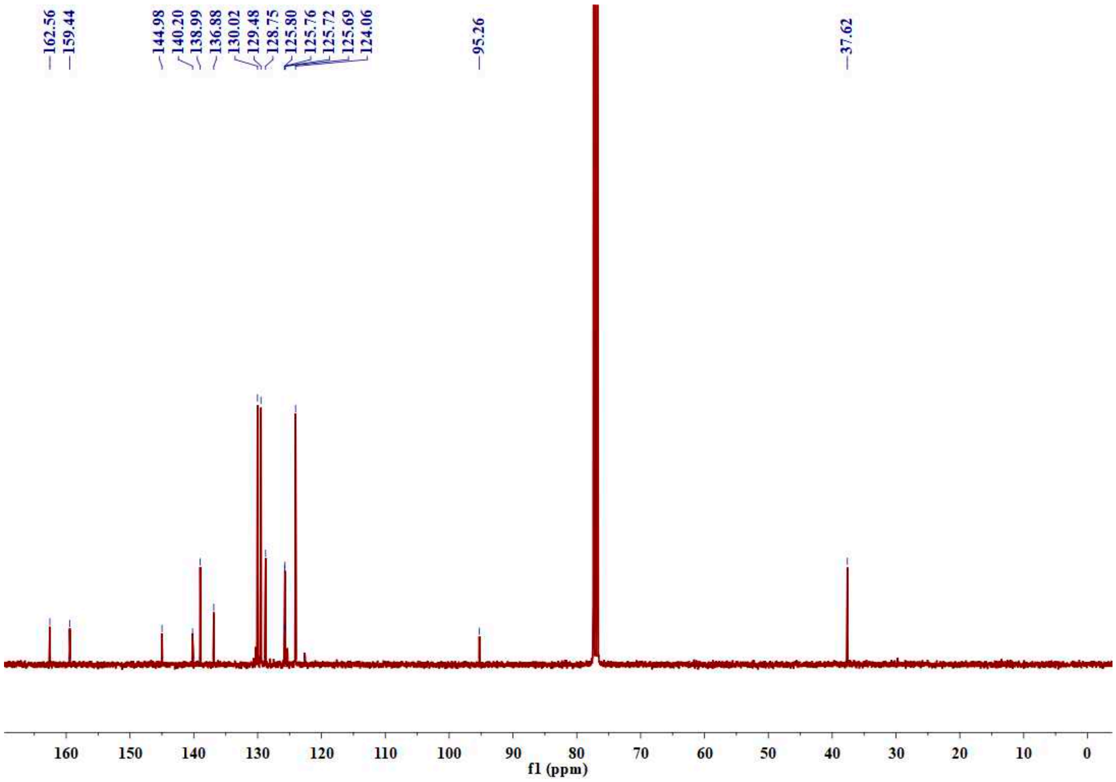
162.56 159.44
144.98 140.20 138.99 136.88 130.02 129.48 128.75 125.80 125.76 125.72 125.69 124.06
95.26
37.62
160 150 140 130 120 110 100 90 80 70 60 50 40 30 20 10 0
f1 (ppm)

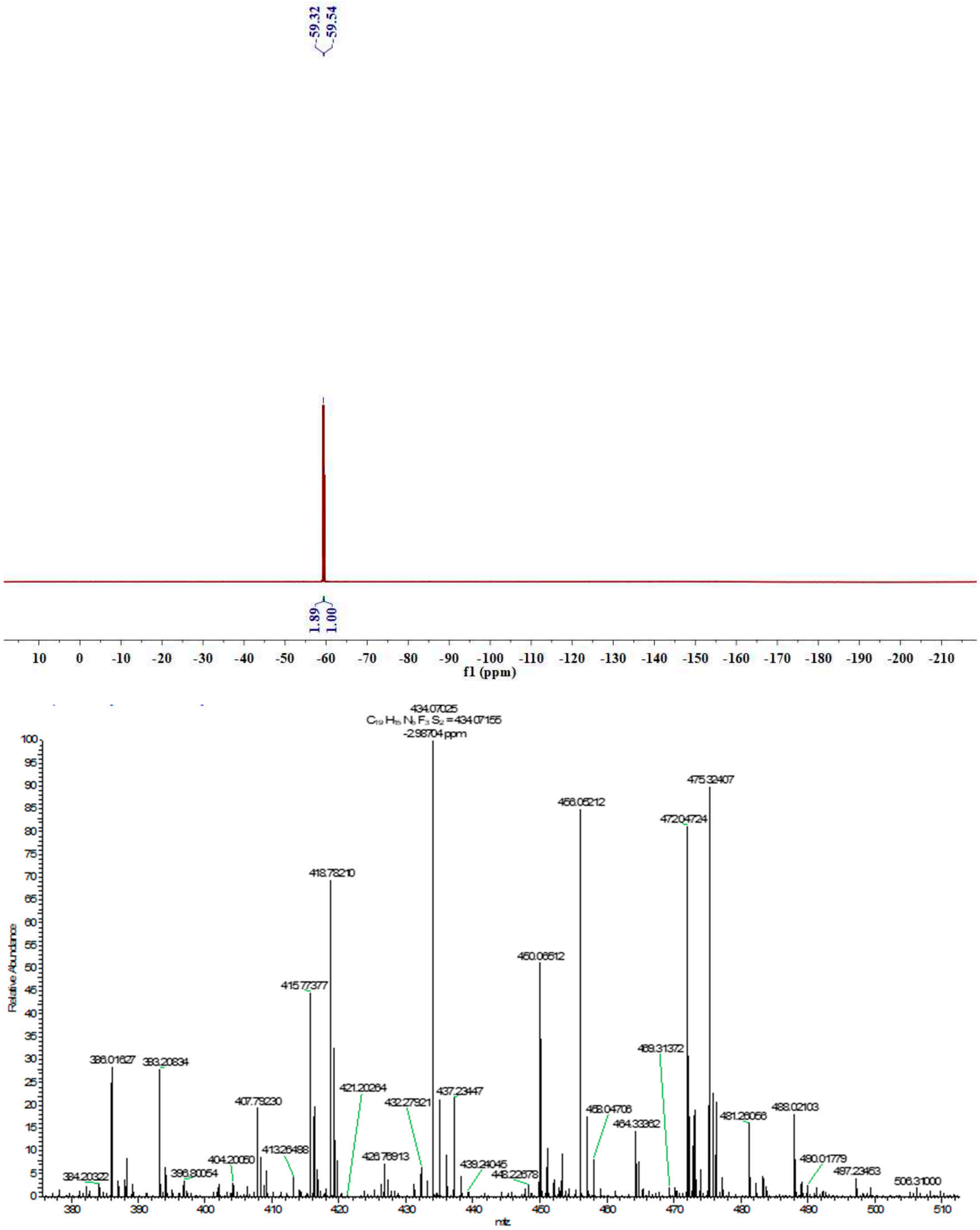

Figure S17. ^{1}H NMR, ^{13}C NMR, ^{19}F NMR and HRMS for title compound **E_{12}**.

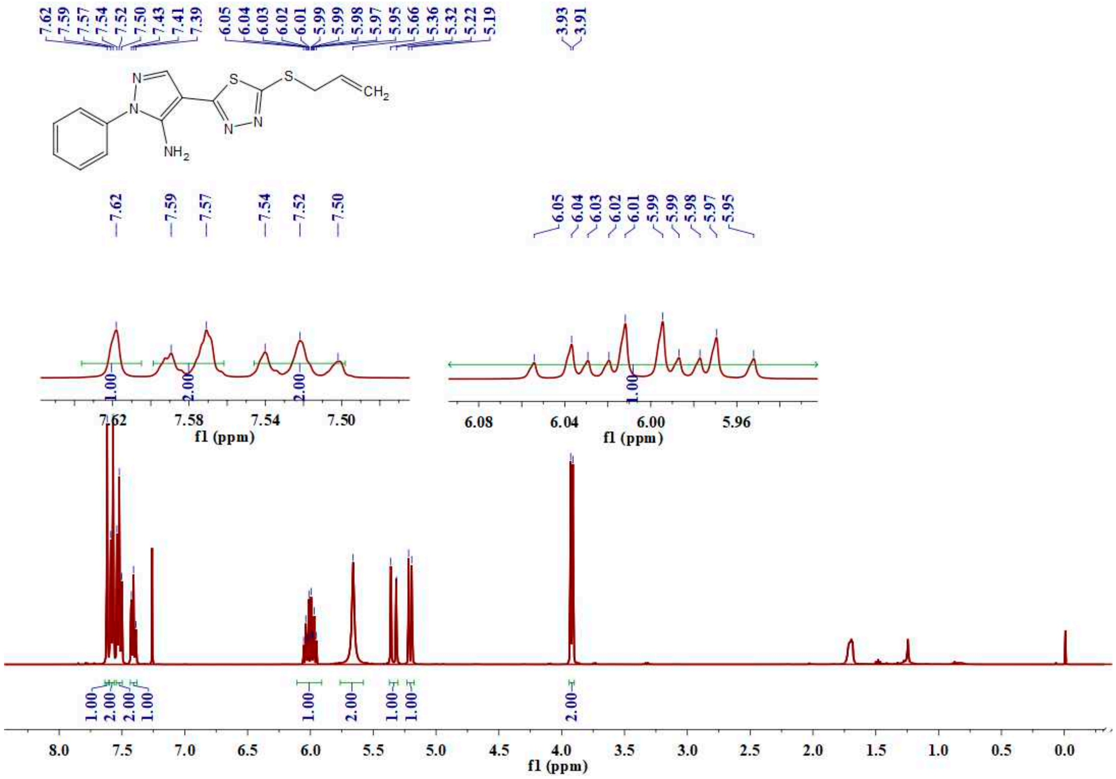
7.62 7.59 7.57 7.54 7.52 7.50 7.43 7.41 7.39 6.05 6.04 6.03 6.02 6.01 5.99 5.99 5.98 5.97 5.95 5.66 5.36 5.32 5.22 5.19
3.93 3.91
N
N
S
N
N
CH2
NH2
1.00
2.00
2.00
7.62 7.58 7.54 7.50
f1 (ppm)
1.00
6.08 6.04 6.00 5.96
f1 (ppm)
1.00 2.00 2.00 1.00
1.00
2.00
1.00 1.00
2.00
8.0 7.5 7.0 6.5 6.0 5.5 5.0 4.5 4.0 3.5 3.0 2.5 2.0 1.5 1.0 0.5 0.0
f1 (ppm)

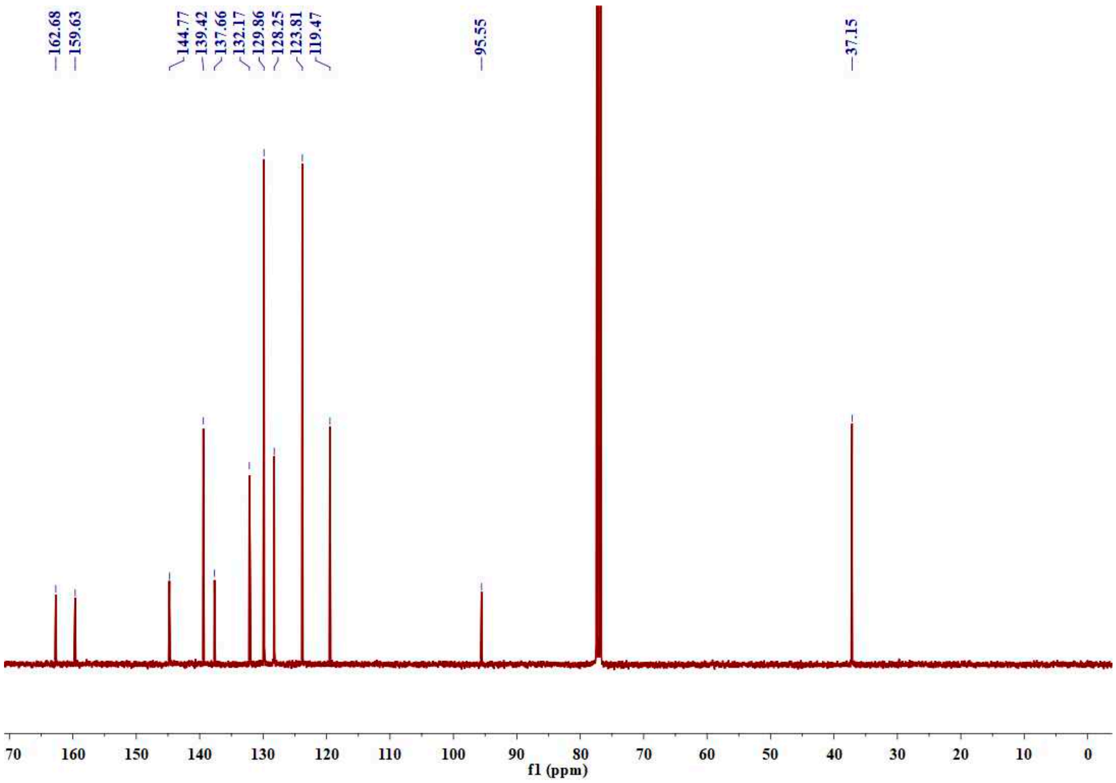
162.68 159.63
144.77 139.42 137.66 132.17 129.86 128.25 123.81 119.47
95.55
37.15
170 160 150 140 130 120 110 100 90 80 70 60 50 40 30 20 10 0
f1 (ppm)

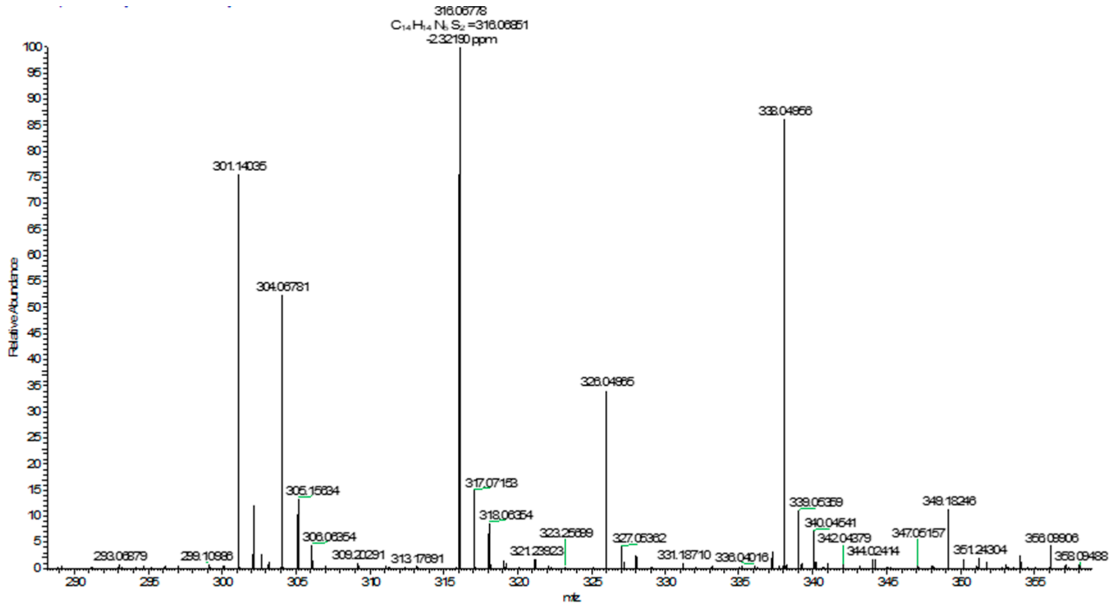

Figure S18. ^{1}H NMR, ^{13}C NMR, ^{19}F NMR and HRMS for title compound **E13**.

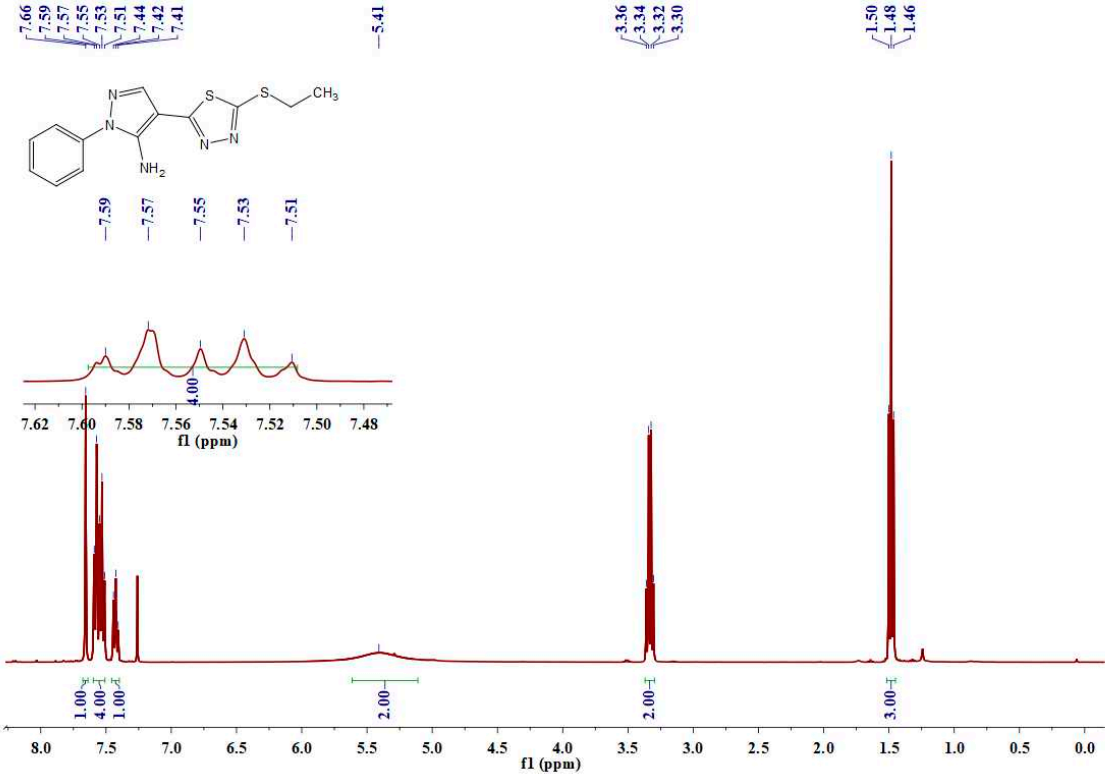

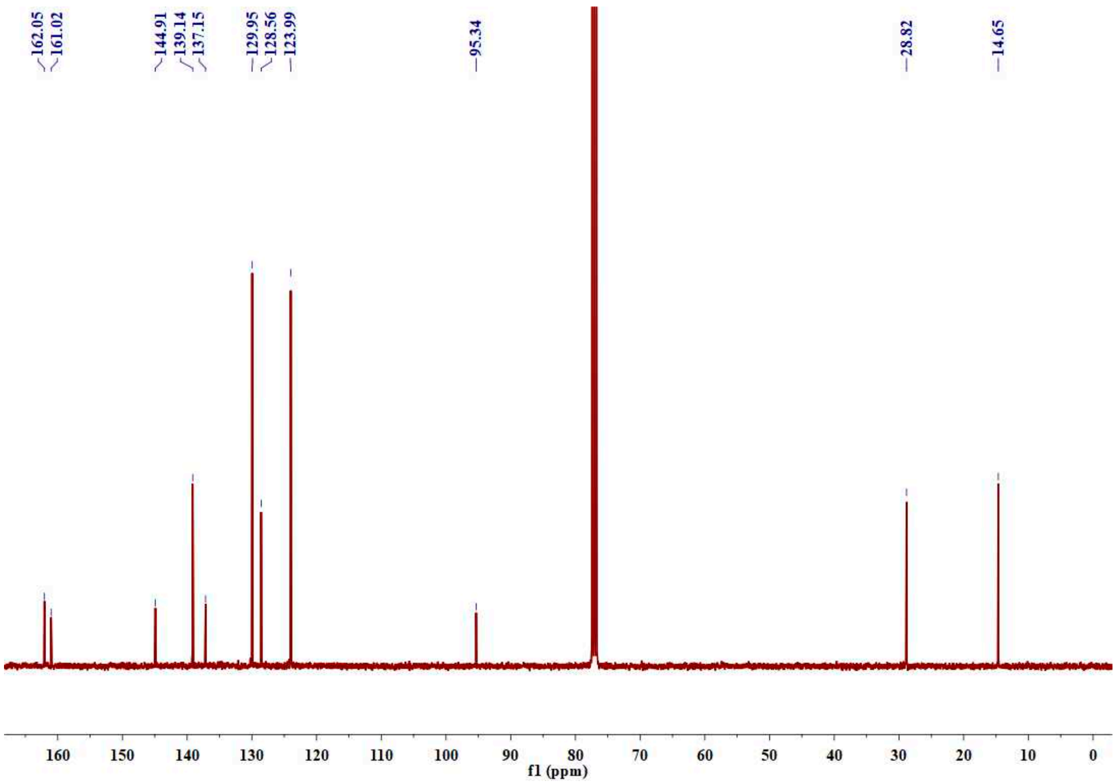

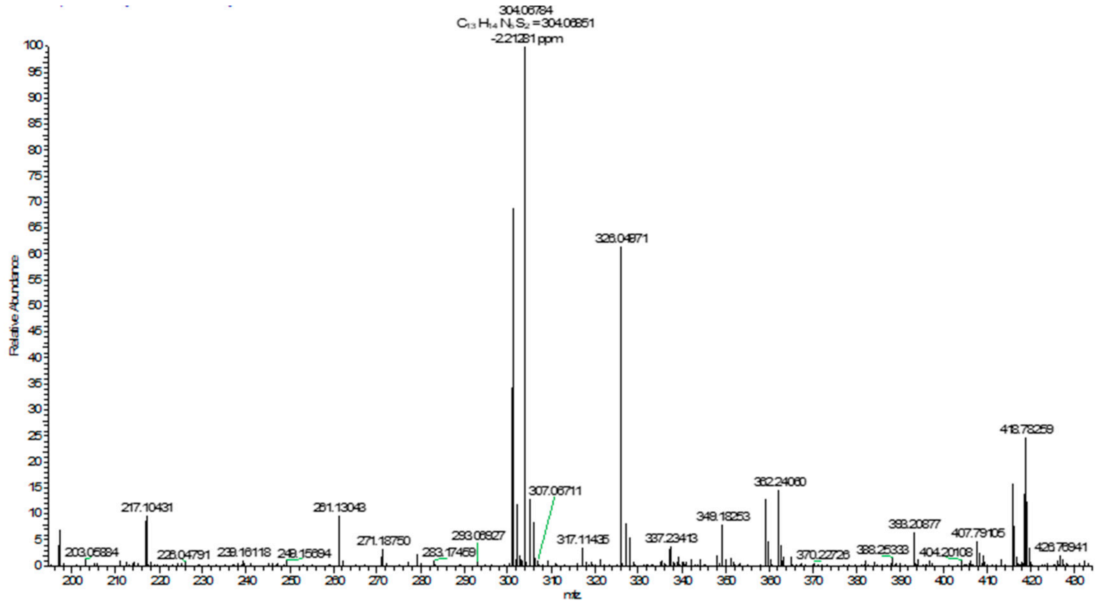

Figure S19. ^{1}H NMR, ^{13}C NMR, ^{19}F NMR and HRMS for title compound **E_{14}**.

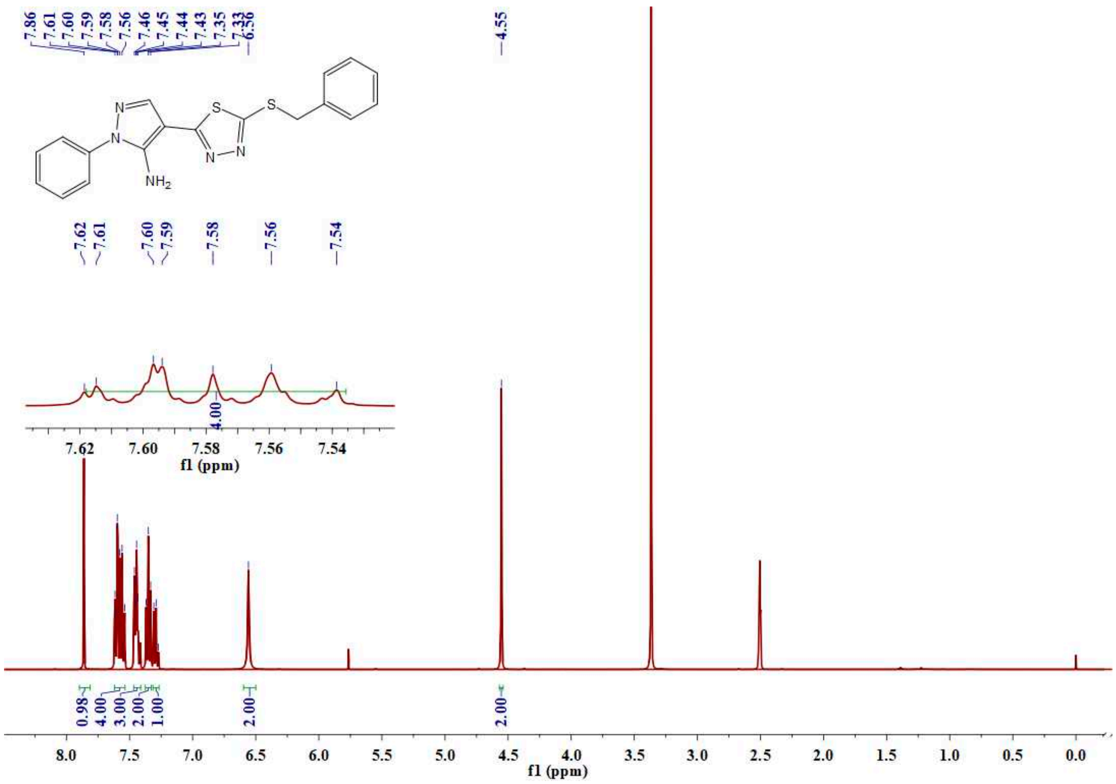
7.86 7.61 7.60 7.59 7.58 7.56 7.46 7.45 7.44 7.43 7.35 7.33 6.56
4.55
7.62 7.61 7.60 7.59 7.58 7.56 7.54
4.00
7.62 7.60 7.58 7.56 7.54
f1 (ppm)
0.98 4.00 3.00 2.00 1.00 2.00 2.00
8.0 7.5 7.0 6.5 6.0 5.5 5.0 4.5 4.0 3.5 3.0 2.5 2.0 1.5 1.0 0.5 0.0
f1 (ppm)
N
N
S
S
N
N
NH2

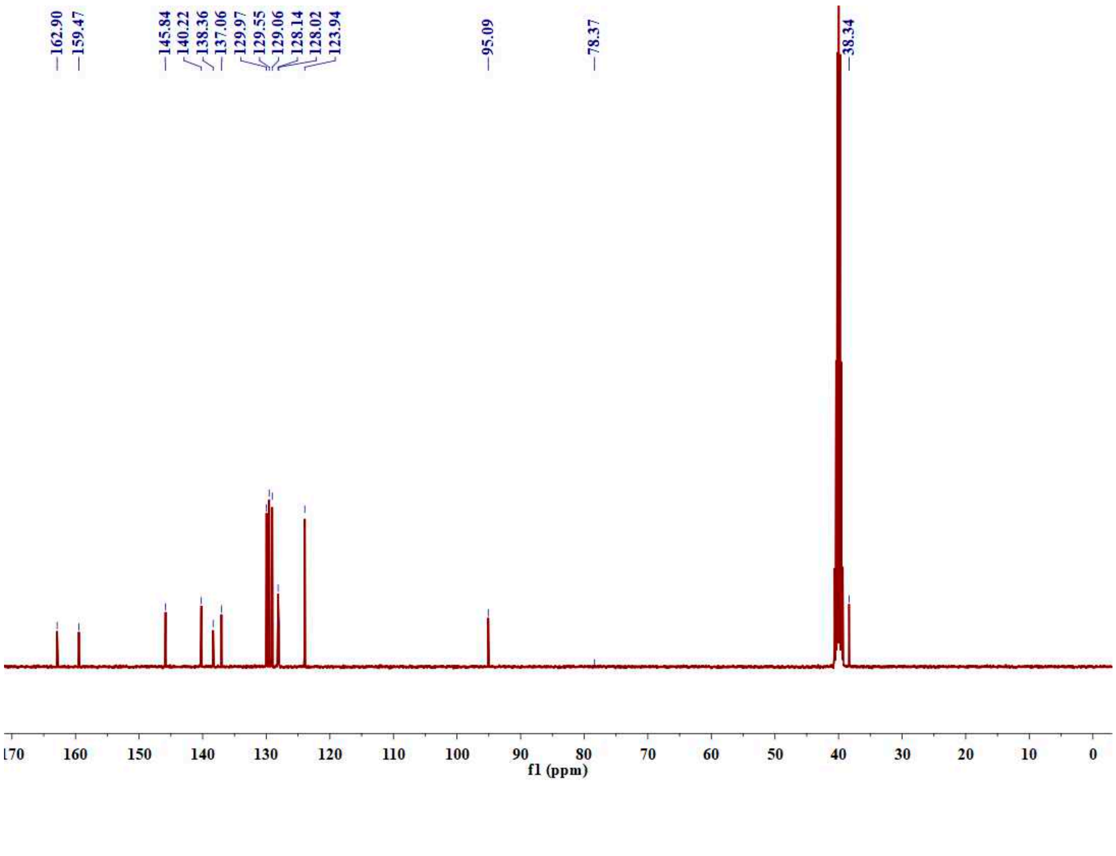
162.90 159.47 145.84 140.22 138.36 137.06 129.97 129.55 129.06 128.14 128.02 123.94
95.09
78.37
38.34
170 160 150 140 130 120 110 100 90 80 70 60 50 40 30 20 10 0
f1 (ppm)

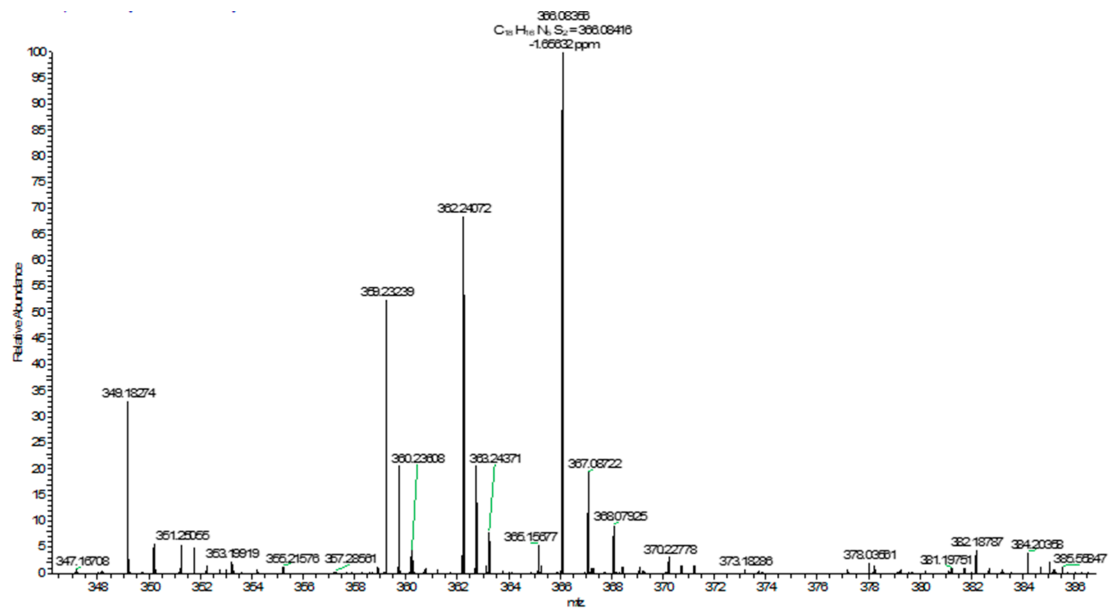

Figure S20. ^{1}H NMR, ^{13}C NMR and HRMS for title compound **E$_{15}$**.

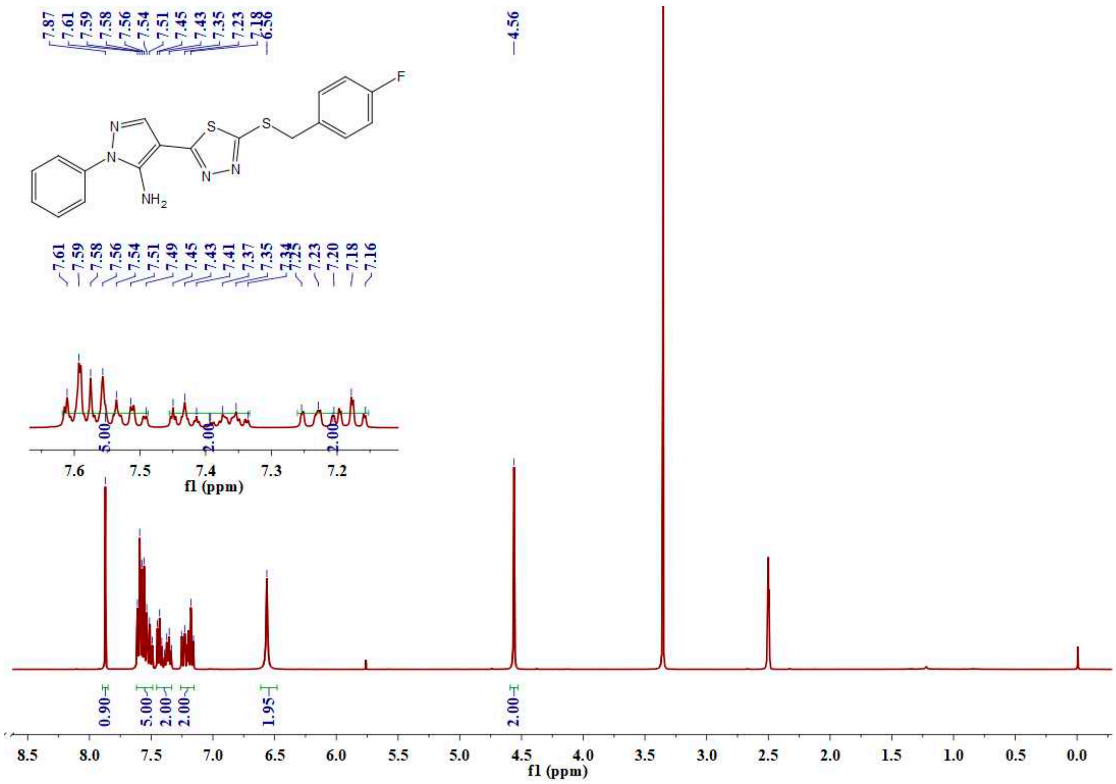

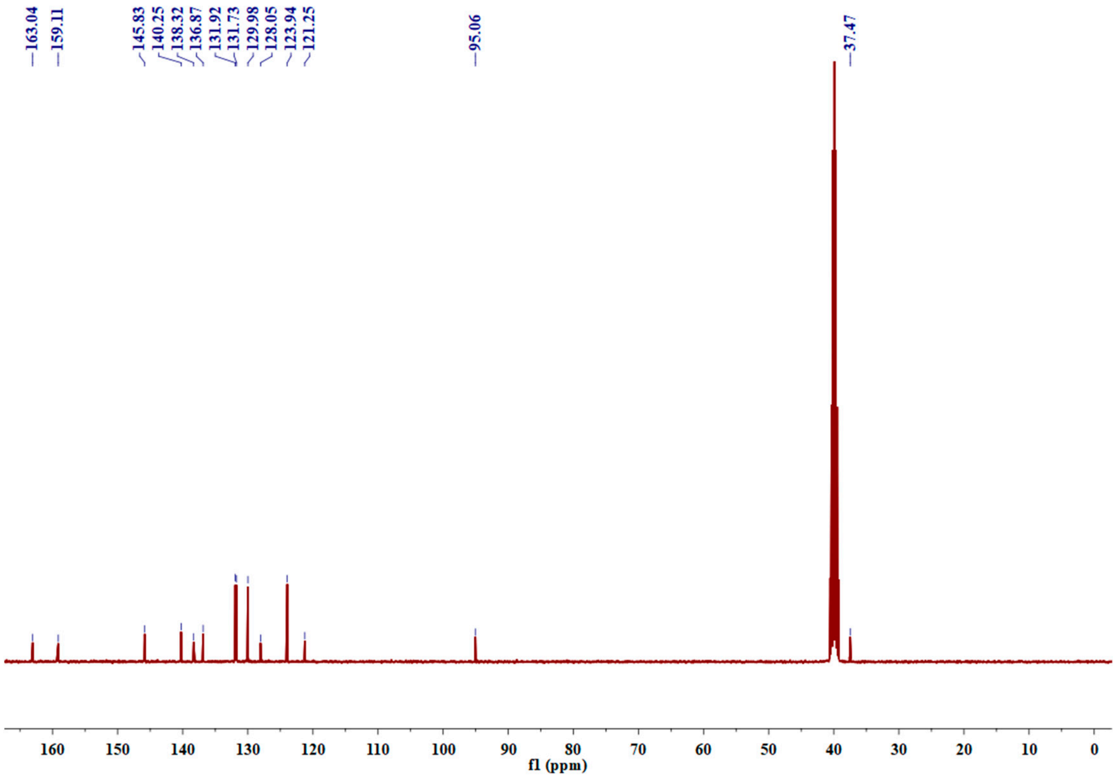
163.04
159.11
145.83
140.25
138.32
136.87
131.92
131.73
129.98
128.05
123.94
121.25
95.06
37.47
160 150 140 130 120 110 100 90 80 70 60 50 40 30 20 10 0
f1 (ppm)

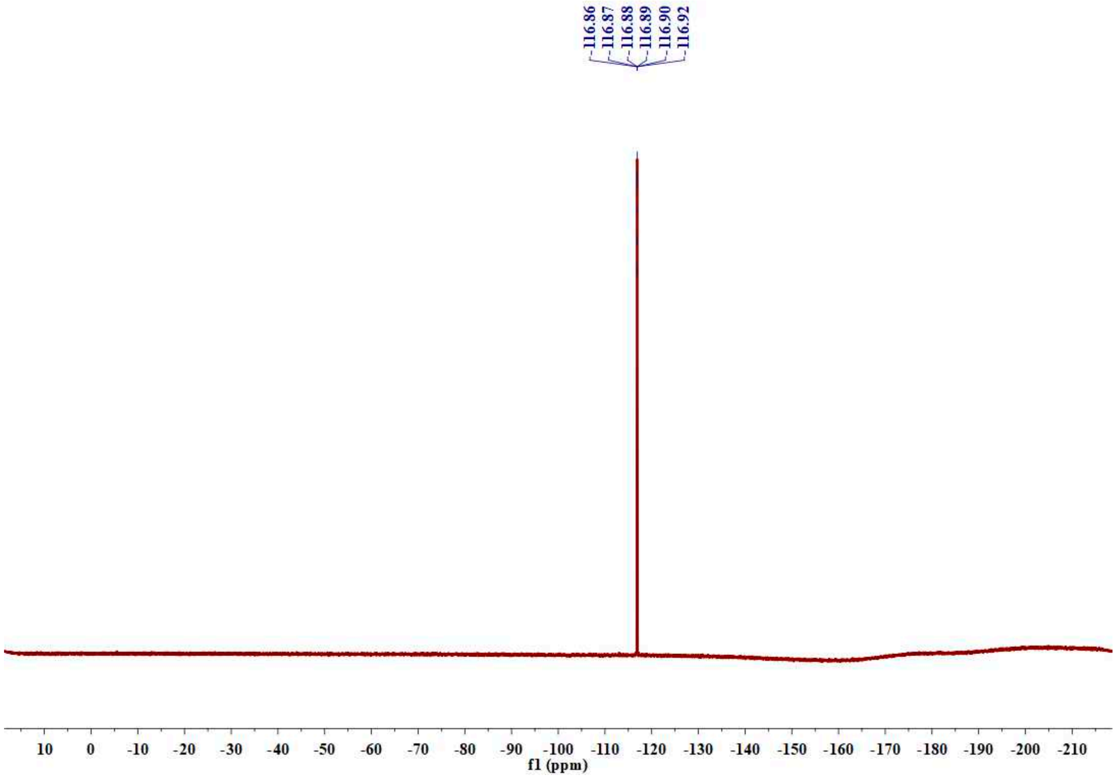
116.86
116.87
116.88
116.89
116.90
116.92
10 0 -10 -20 -30 -40 -50 -60 -70 -80 -90 -100 -110 -120 -130 -140 -150 -160 -170 -180 -190 -200 -210
f1 (ppm)

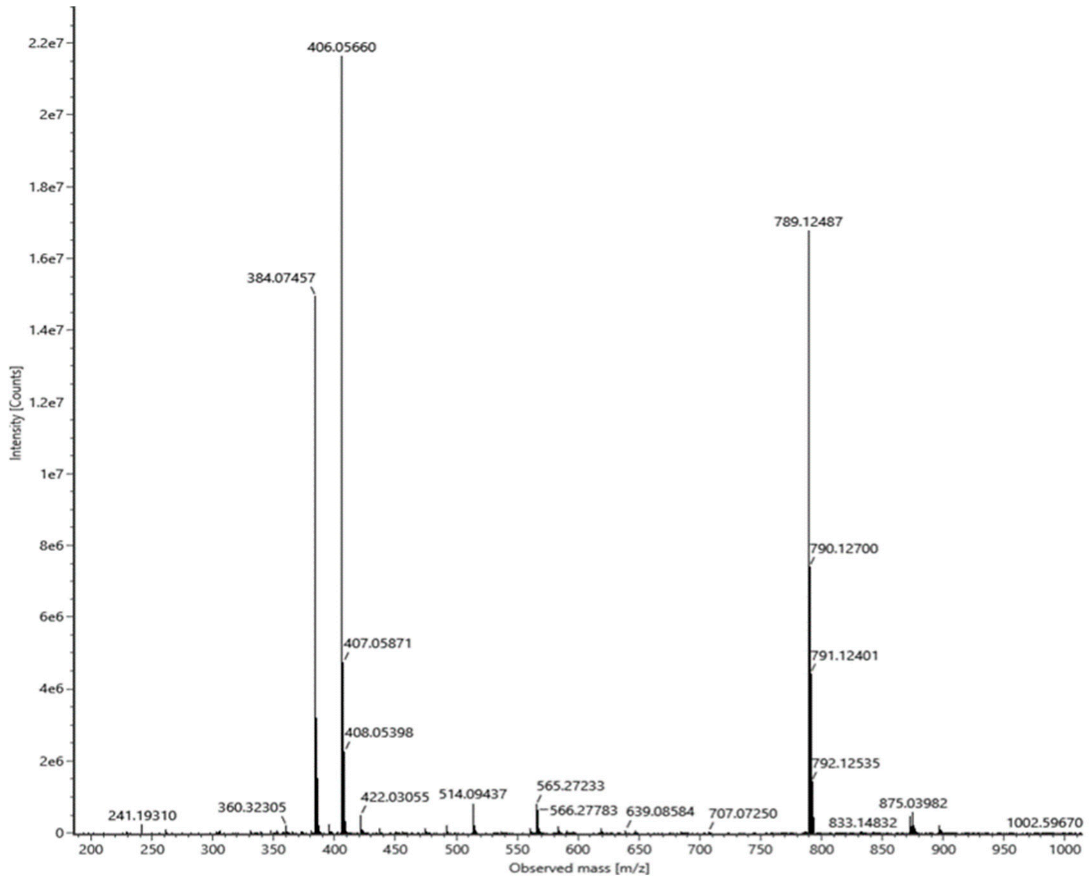

Figure S21. ^{1}H NMR, ^{13}C NMR and HRMS for title compound $\mathbf{E_{16}}$.

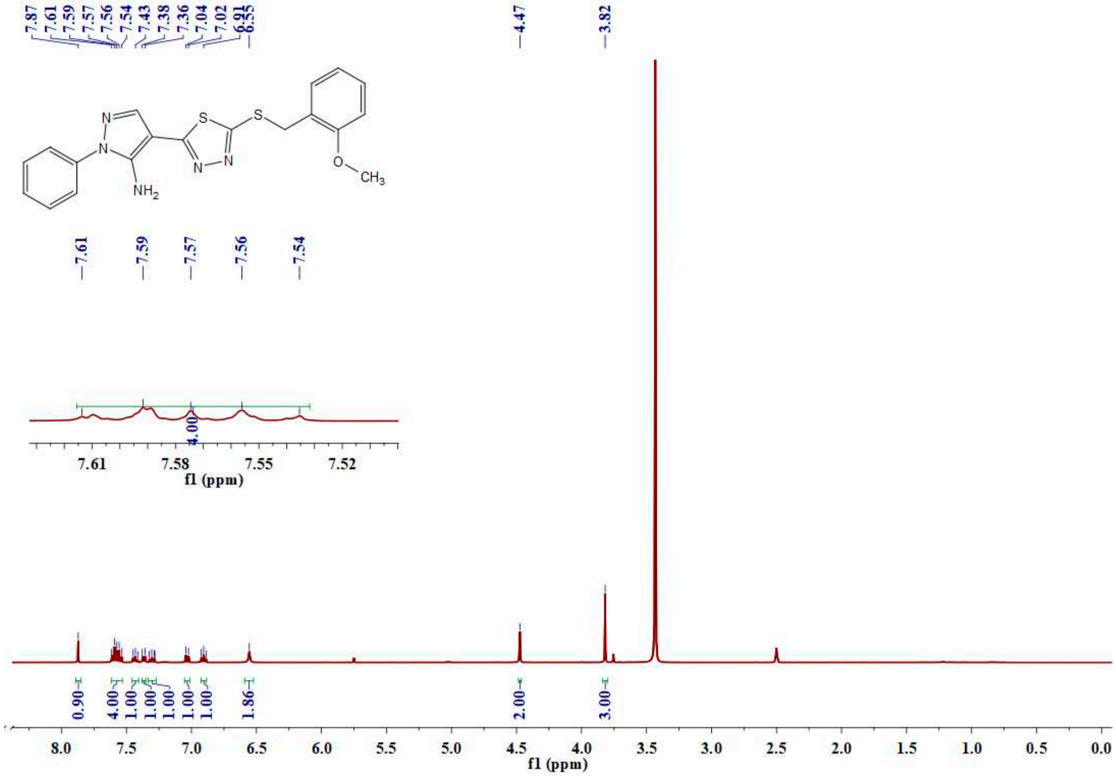

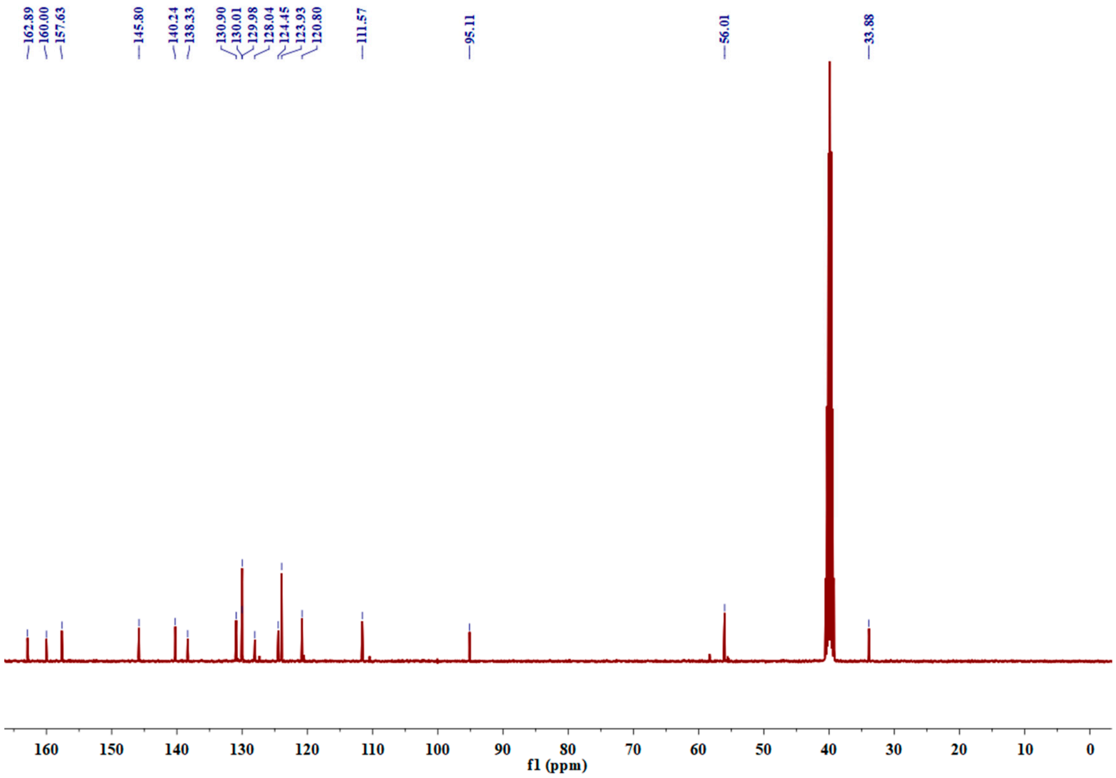

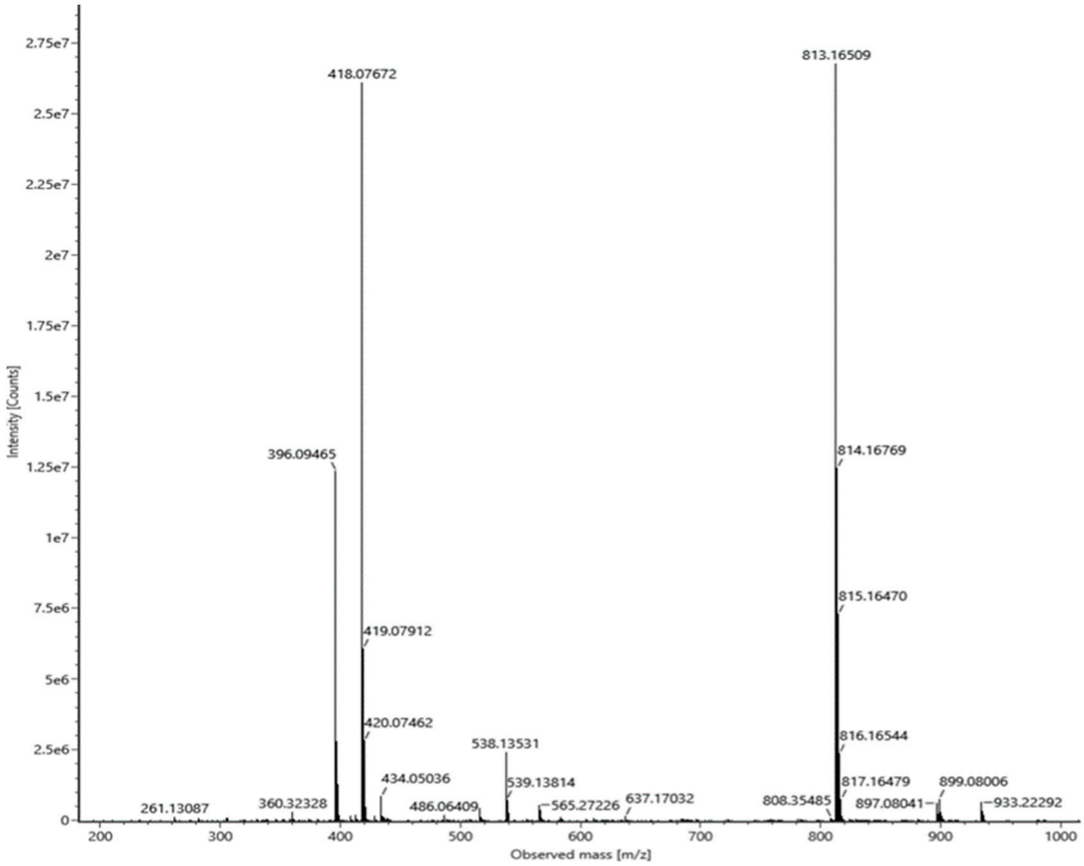

Figure S22. ^{1}H NMR, ^{13}C NMR and HRMS for title compound **E_{17}**.

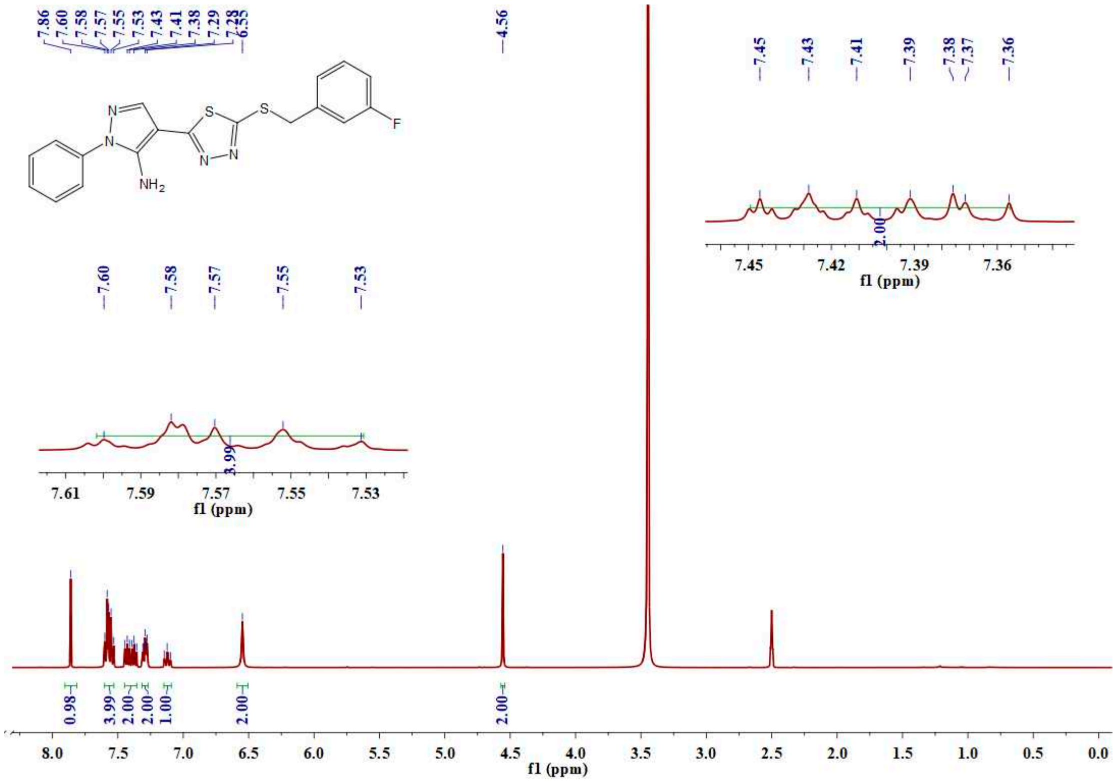
7.86
7.60
7.58
7.57
7.55
7.53
7.43
7.41
7.38
7.29
7.28
6.55
4.56
N
N
S
N
N
S
F
NH2
7.45
7.43
7.41
7.39
7.38
7.37
7.36
2.00
7.45
7.42
7.39
7.36
f1 (ppm)
7.60
7.58
7.57
7.55
7.53
3.99
7.61
7.59
7.57
7.55
7.53
f1 (ppm)
0.98
3.99
2.00
2.00
1.00
2.00
2.00
8.0
7.5
7.0
6.5
6.0
5.5
5.0
4.5
4.0
3.5
3.0
2.5
2.0
1.5
1.0
0.5
0.0
f1 (ppm)

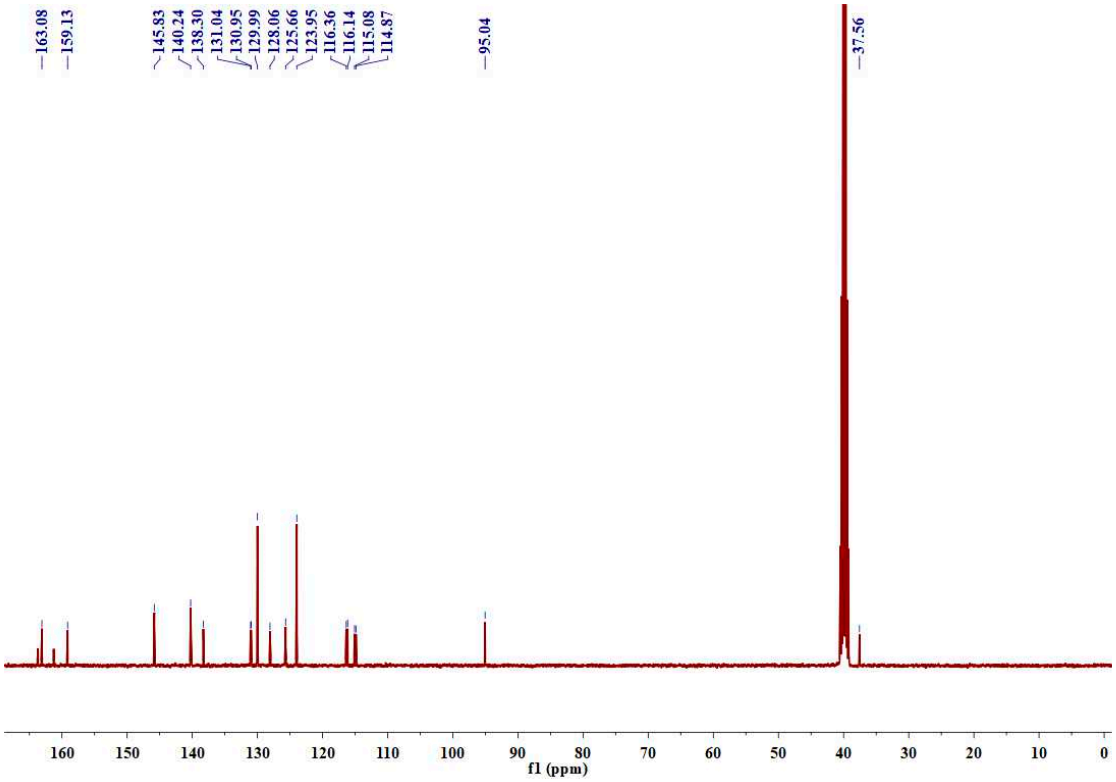
163.08
159.13
145.83
140.24
138.30
131.04
130.95
129.99
128.06
125.66
123.95
116.36
116.14
115.08
114.87
95.04
37.56
160
150
140
130
120
110
100
90
80
70
60
50
40
30
20
10
0
f1 (ppm)

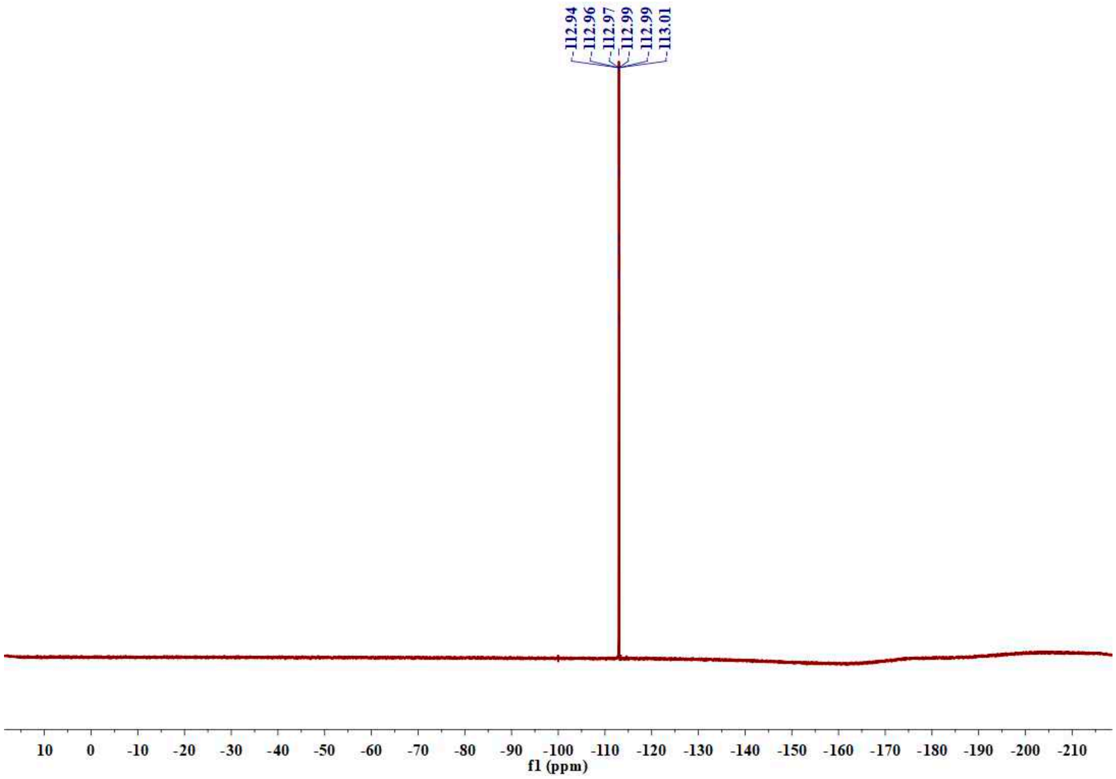

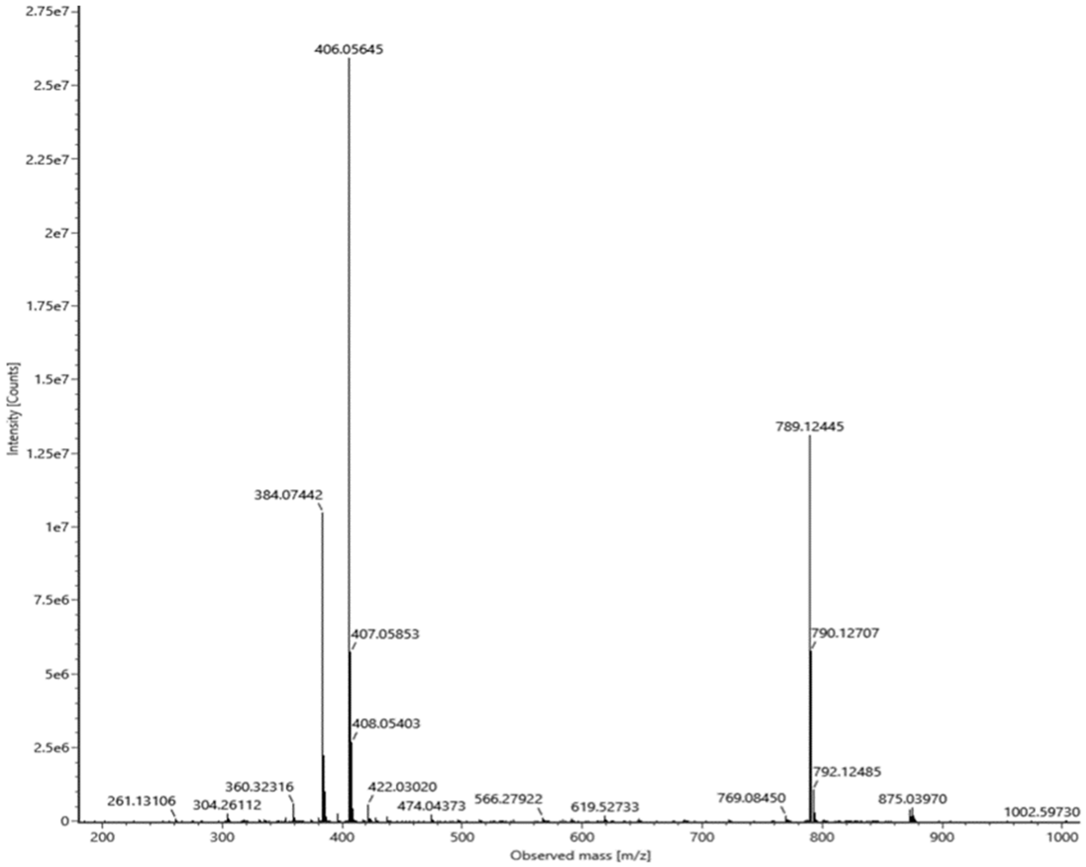

Figure S23. ^{1}H NMR, ^{13}C NMR, ^{19}F NMR and HRMS for title compound **E_{18}**.

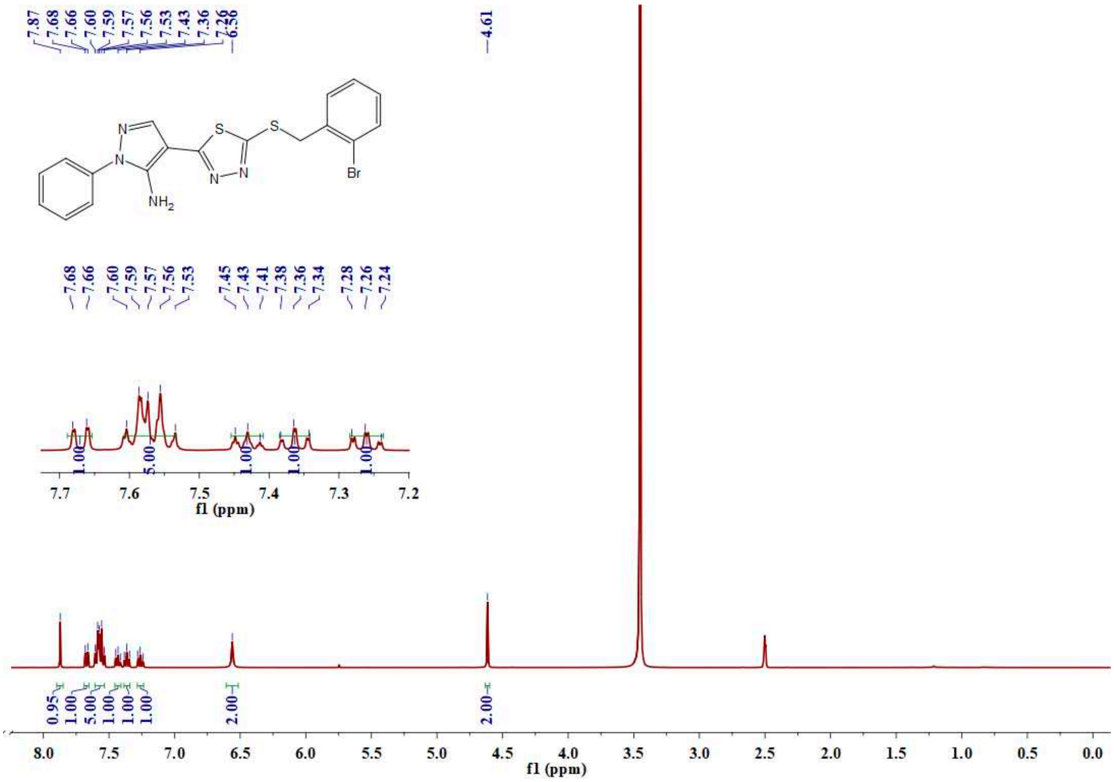
7.87 7.68 7.66 7.60 7.59 7.57 7.56 7.53 7.43 7.36 7.26 6.56
4.61
N N S S N N NH2 Br
7.68 7.66 7.60 7.59 7.57 7.56 7.53 7.45 7.43 7.41 7.38 7.36 7.34 7.28 7.26 7.24
1.00 5.00 1.00 1.00
f1 (ppm)
0.95 1.00 5.00 1.00 1.00 1.00 2.00 2.00
f1 (ppm)

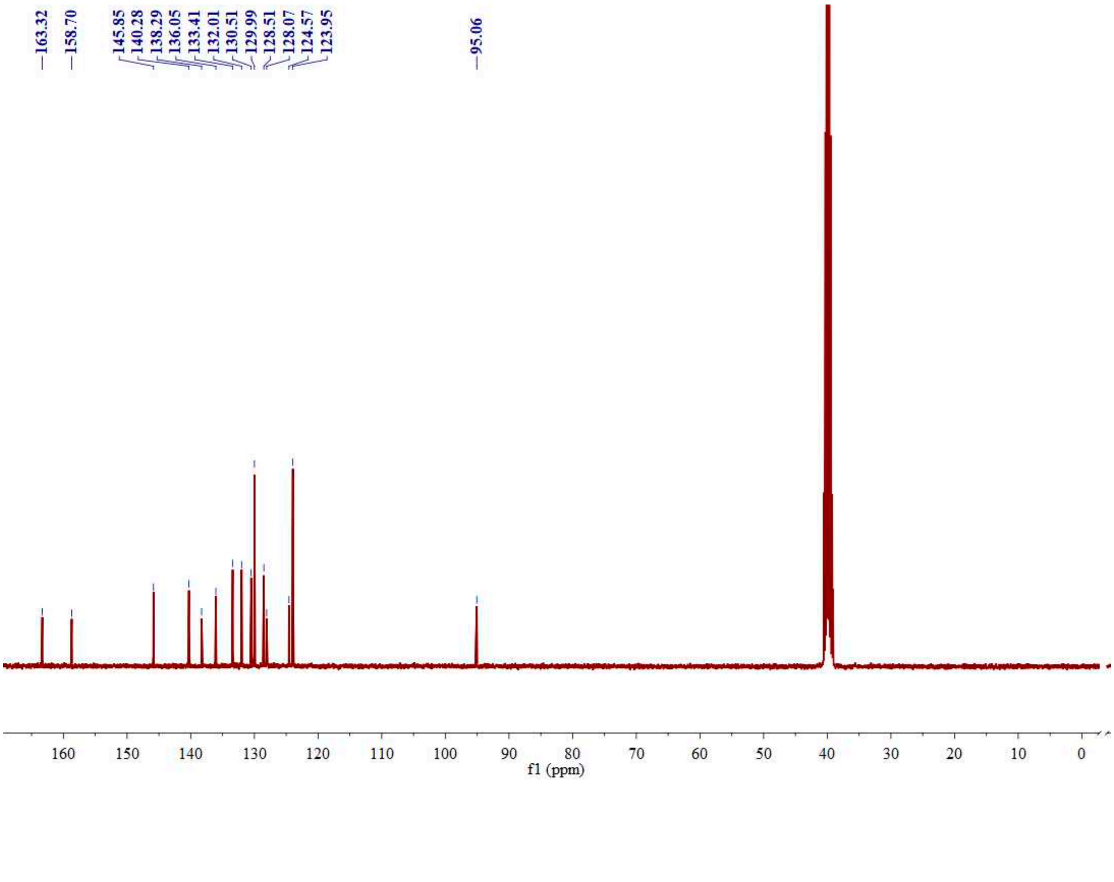
163.32 158.70 145.85 140.28 138.29 136.05 133.41 132.01 130.51 129.99 128.51 128.07 124.57 123.95 95.06
f1 (ppm)

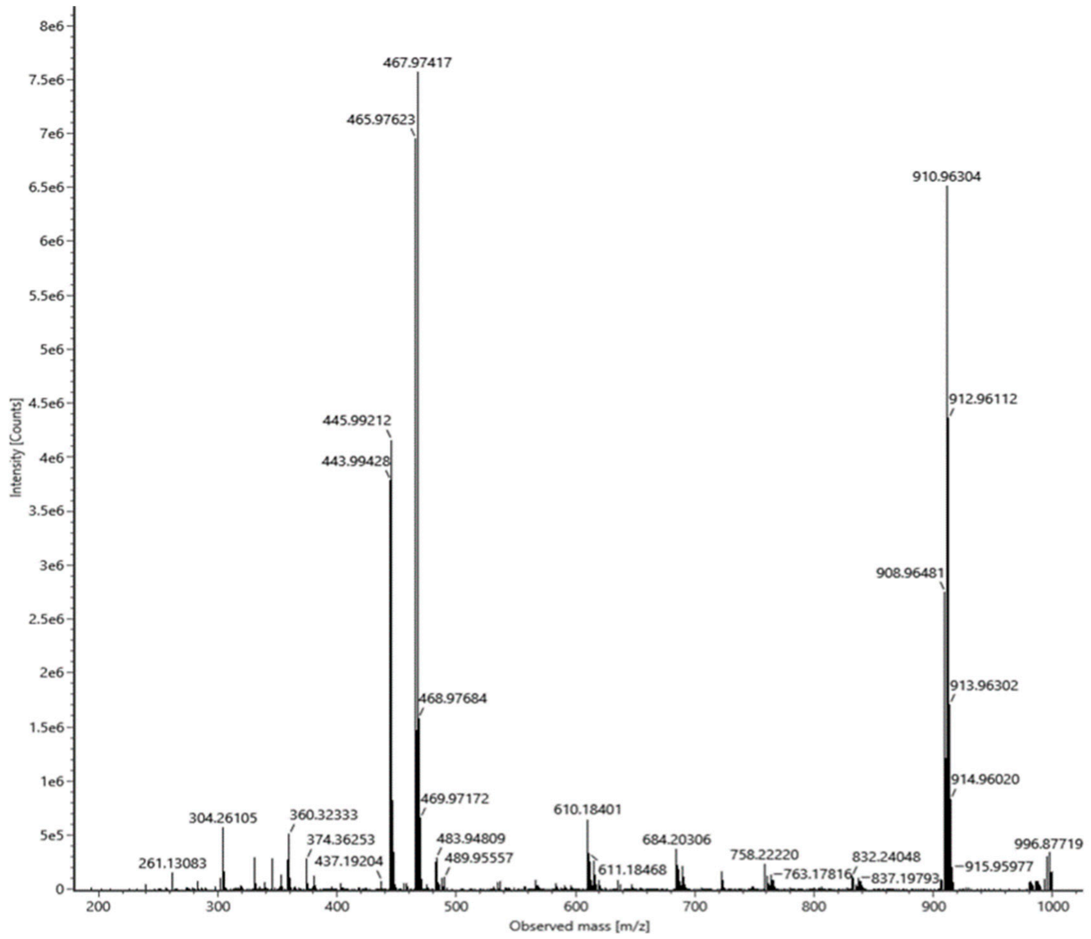

Figure S24. ^{1}H NMR, ^{13}C NMR and HRMS for title compound **E₁₉**.

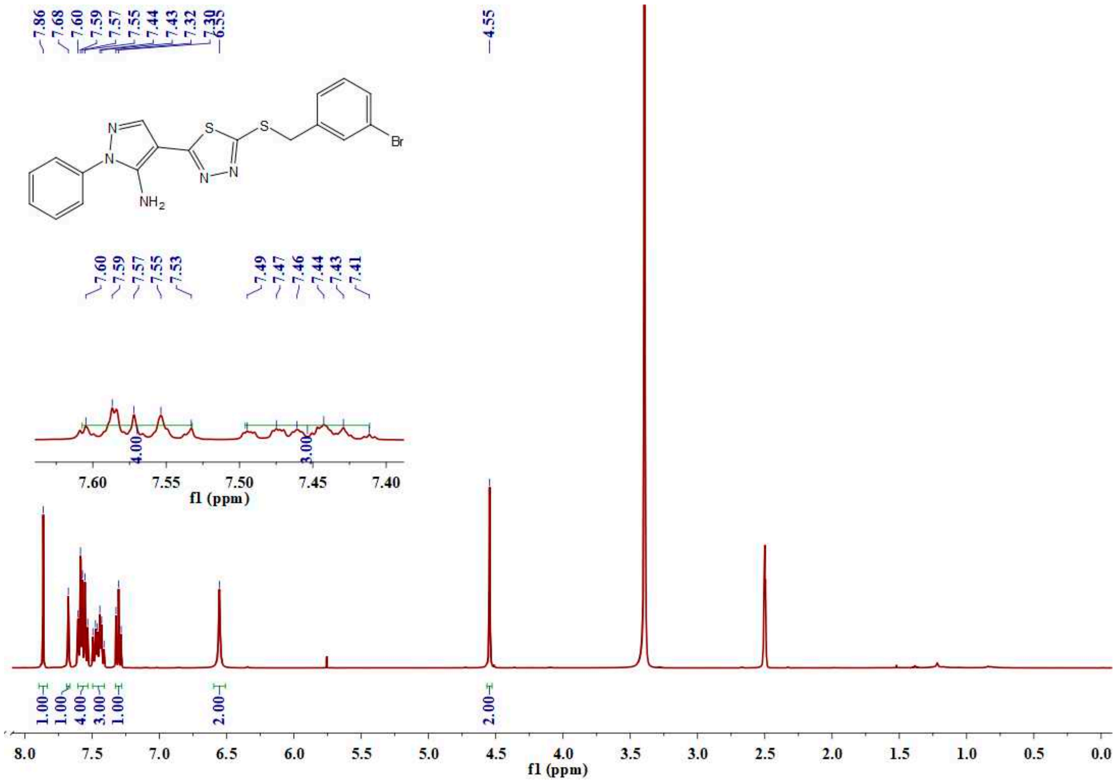

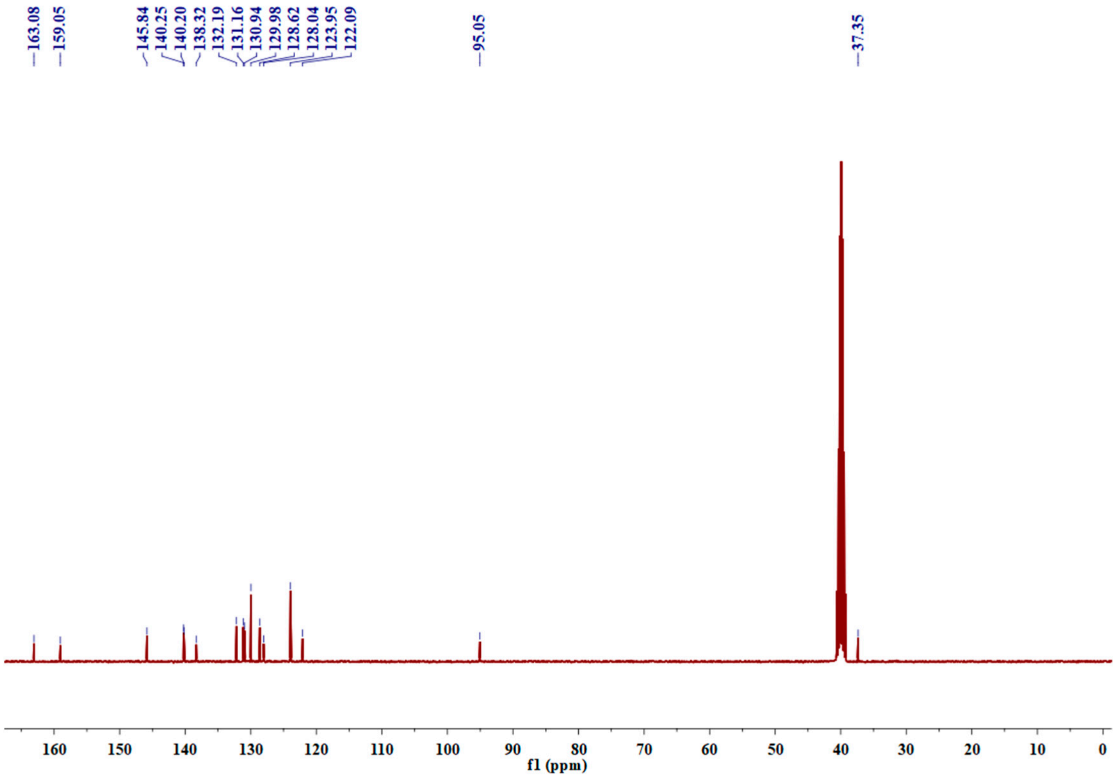

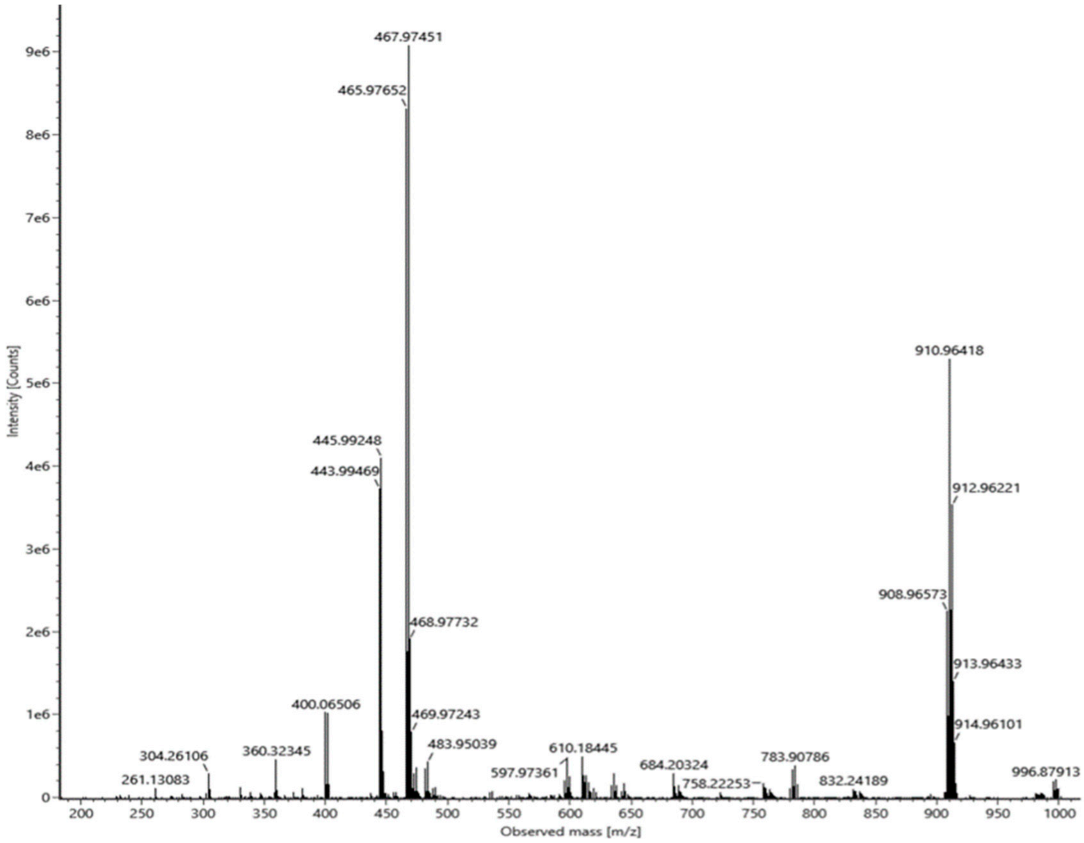

Figure S25. ^{1}H NMR, ^{13}C NMR and HRMS for title compound **E_{20}**.

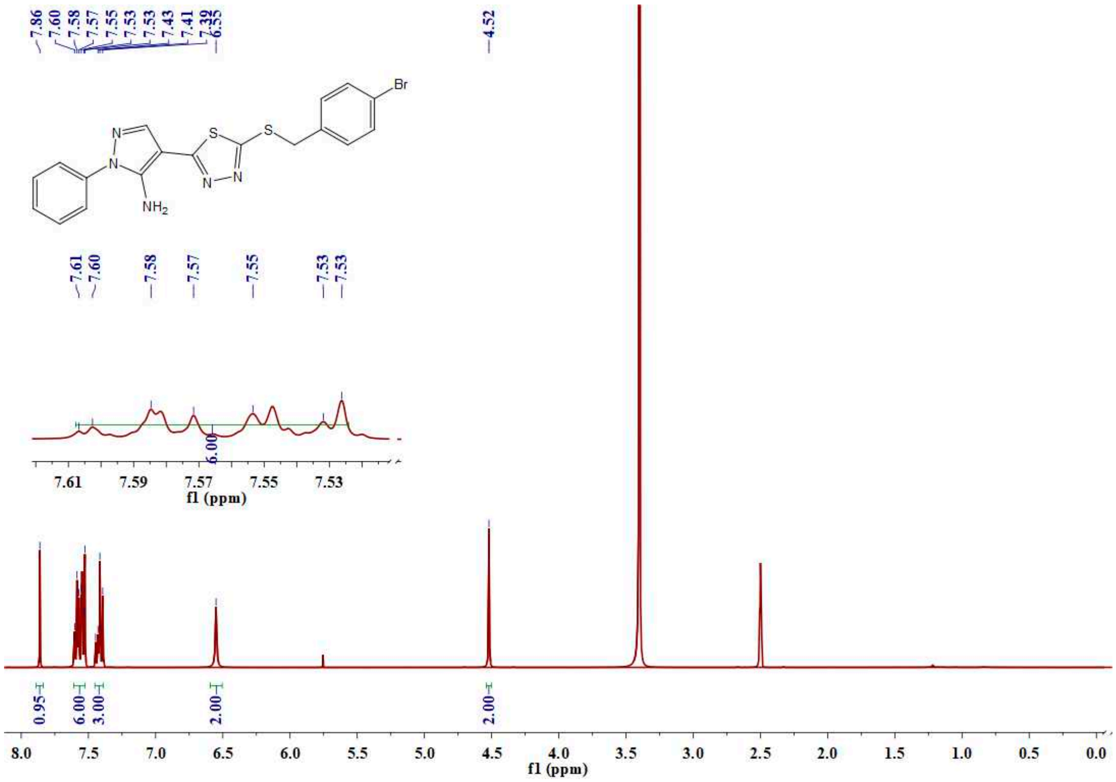
7.86
7.60
7.58
7.57
7.55
7.53
7.53
7.43
7.41
7.39
6.55
4.52
Br
S
N
N
N
N
S
NH2
7.61
7.60
7.58
7.57
7.55
7.53
7.53
6.00
7.61 7.59 7.57 7.55 7.53
f1 (ppm)
0.95
6.00
3.00
2.00
2.00
8.0 7.5 7.0 6.5 6.0 5.5 5.0 4.5 4.0 3.5 3.0 2.5 2.0 1.5 1.0 0.5 0.0
f1 (ppm)

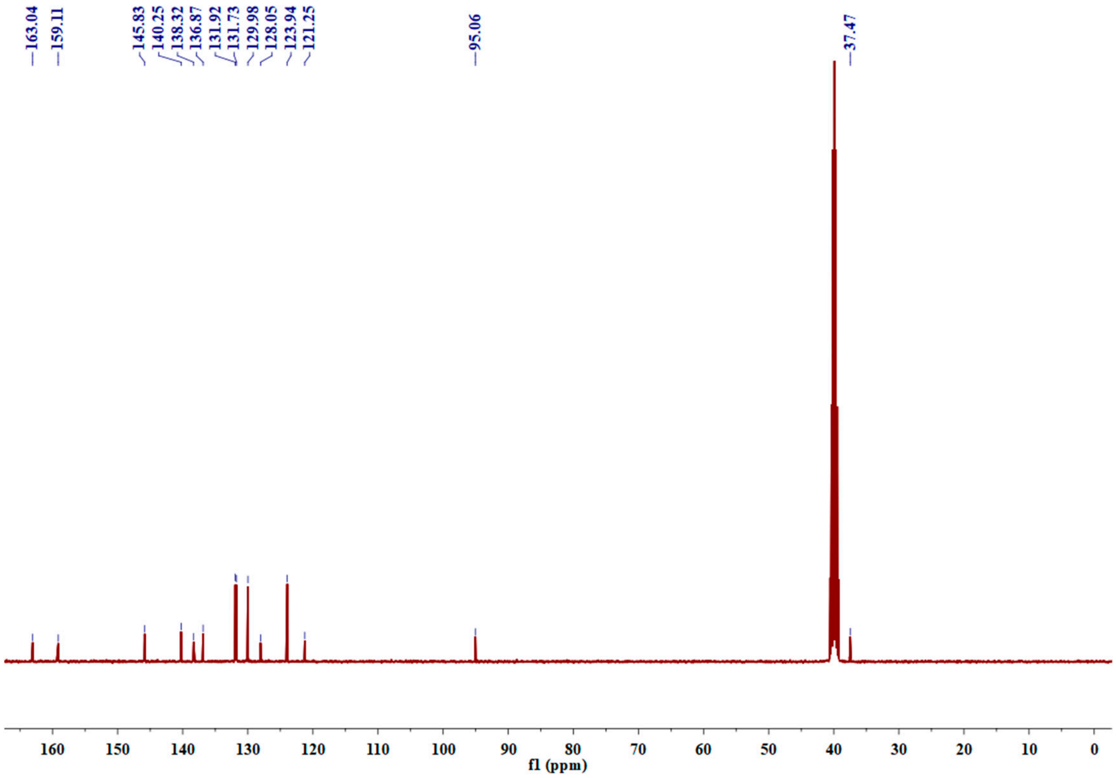
163.04
159.11
145.83
140.25
138.32
136.87
131.92
131.73
129.98
128.05
123.94
121.25
95.06
37.47
160 150 140 130 120 110 100 90 80 70 60 50 40 30 20 10 0
f1 (ppm)

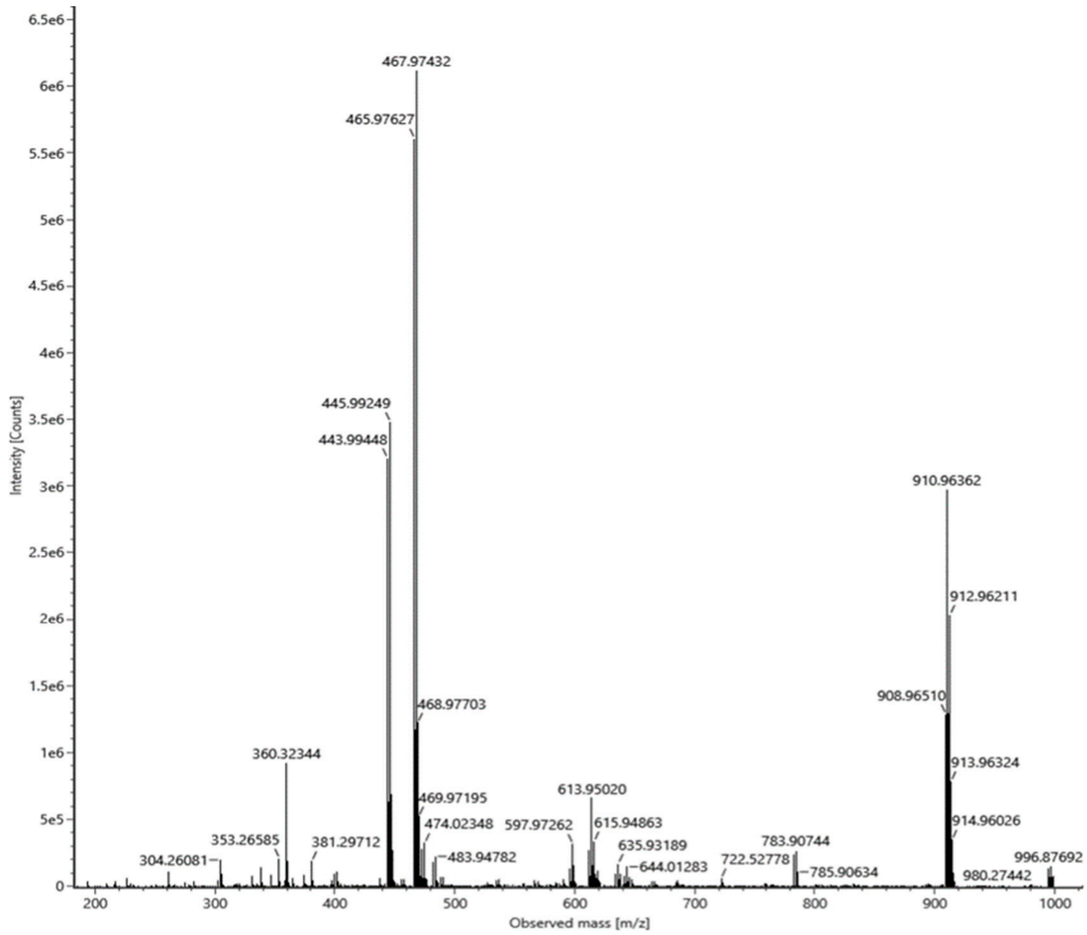

Figure S26. ^{1}H NMR, ^{13}C NMR and HRMS for title compound **E_{21}**.

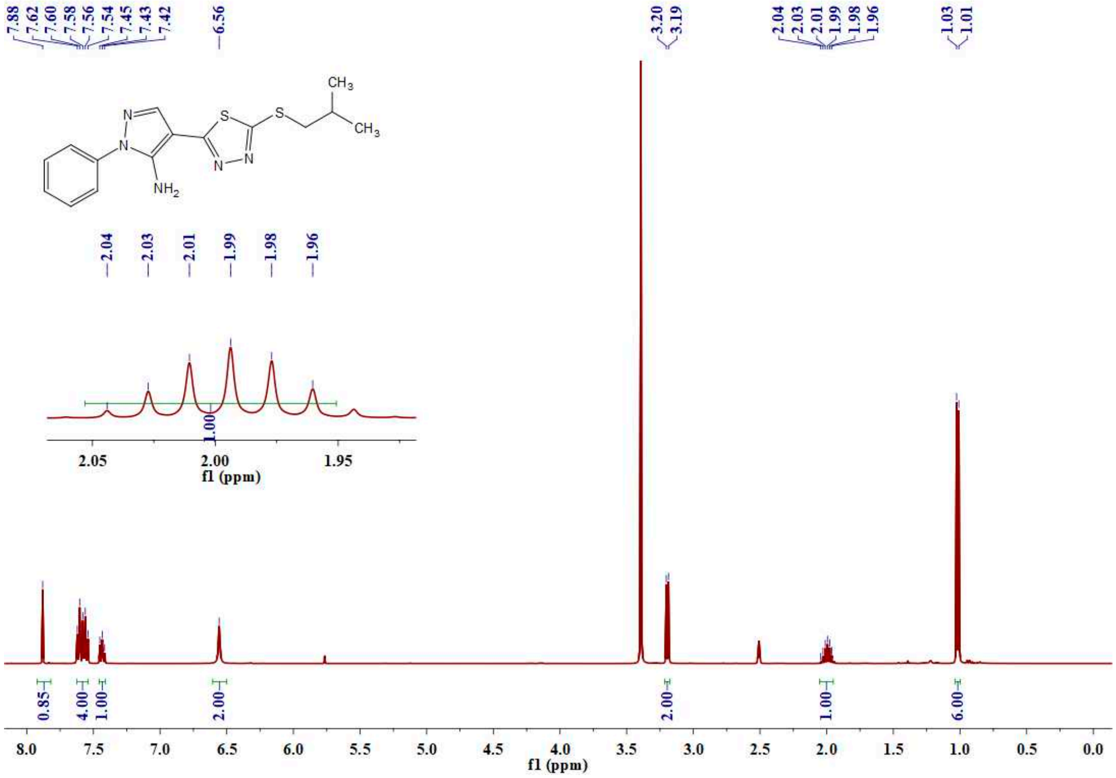

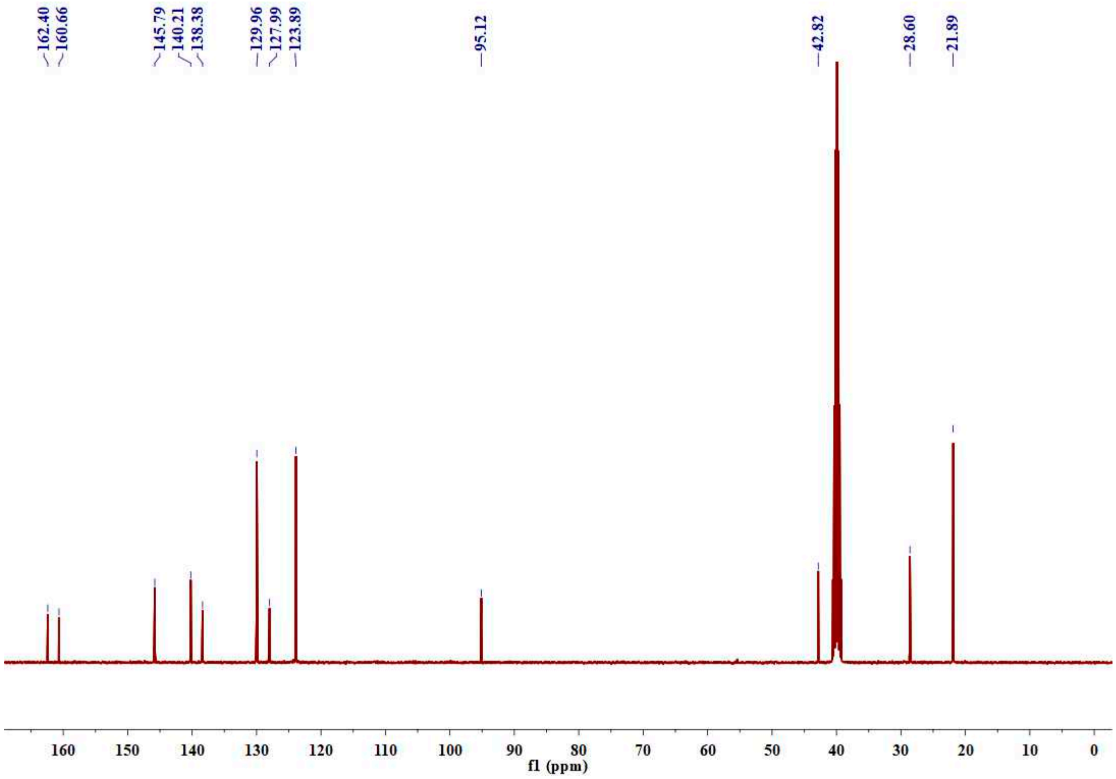

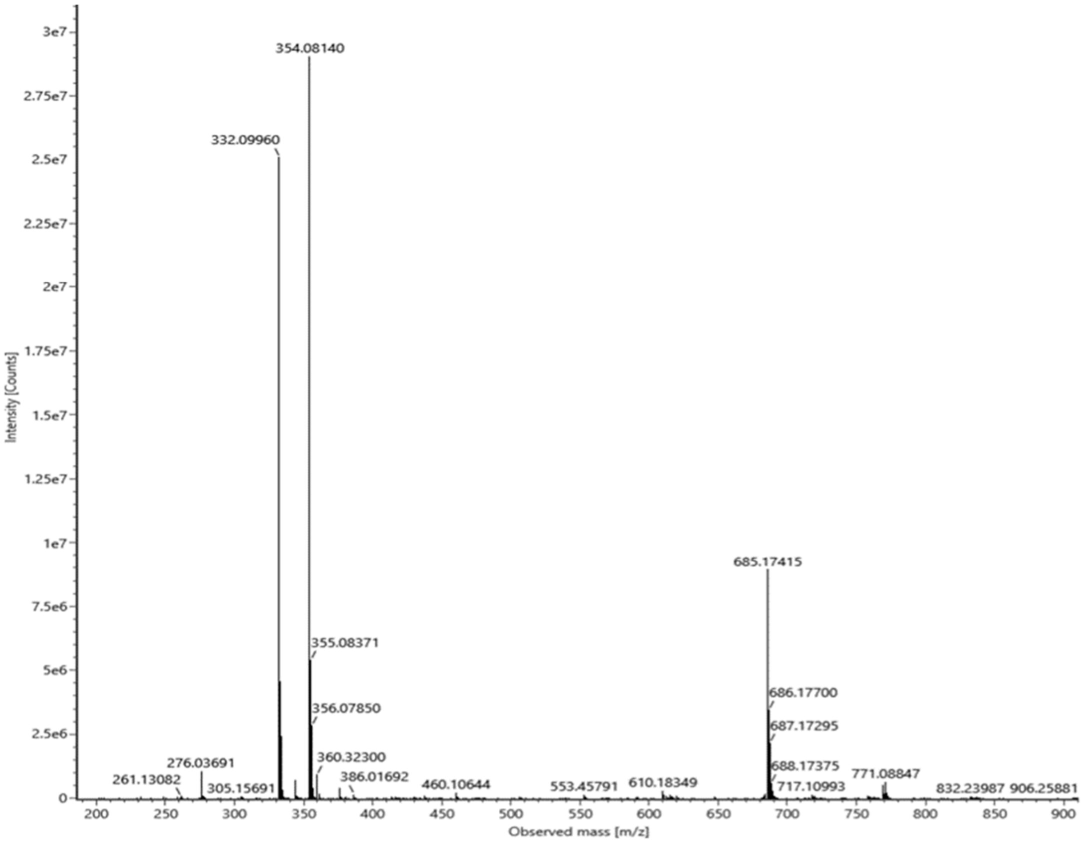

Figure S27. ^{1}H NMR, ^{13}C NMR and HRMS for title compound $\mathbf{E_{22}}$.

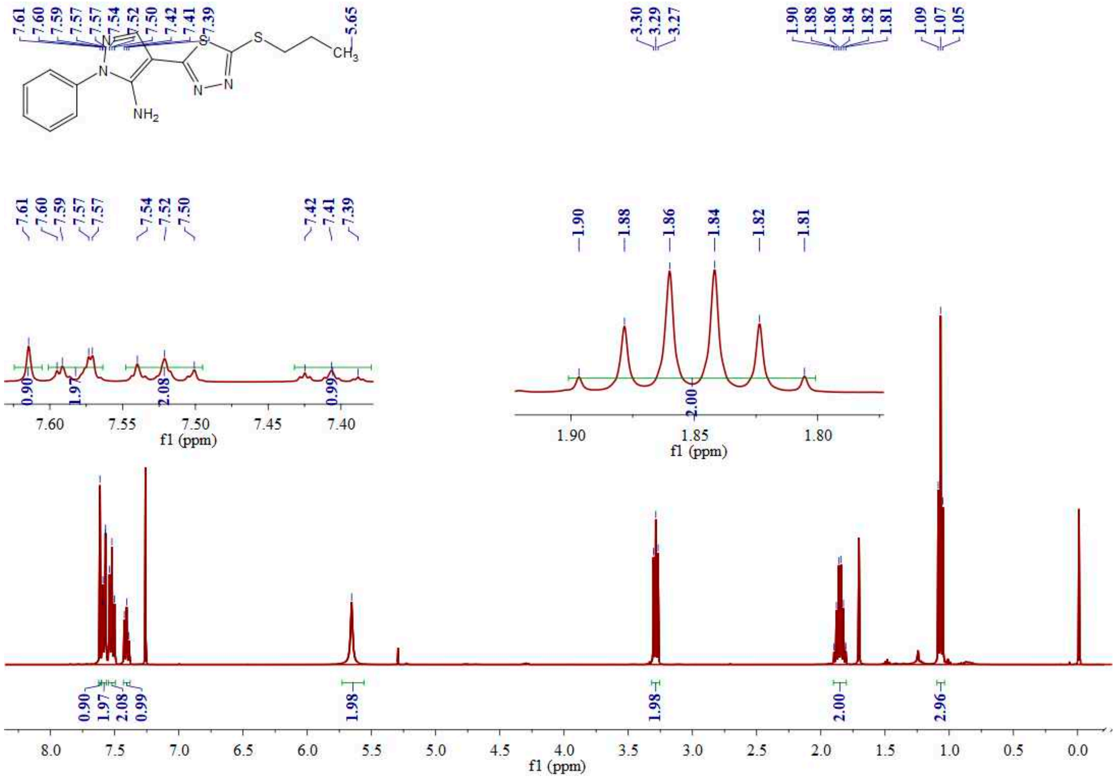
7.61 7.60 7.59 7.57 7.57 7.54 7.52 7.50 7.42 7.41 7.39 5.65
3.30 3.29 3.27
1.90 1.88 1.86 1.84 1.82 1.81
1.09 1.07 1.05
N
N
S
S
N
N
CH3
NH2
0.90 1.97 2.08 0.99
7.60 7.55 7.50 7.45 7.40
f1 (ppm)
2.00
1.90 1.85 1.80
f1 (ppm)
0.90 1.97 2.08 0.99 1.98 1.98 2.00 2.96
8.0 7.5 7.0 6.5 6.0 5.5 5.0 4.5 4.0 3.5 3.0 2.5 2.0 1.5 1.0 0.5 0.0
f1 (ppm)

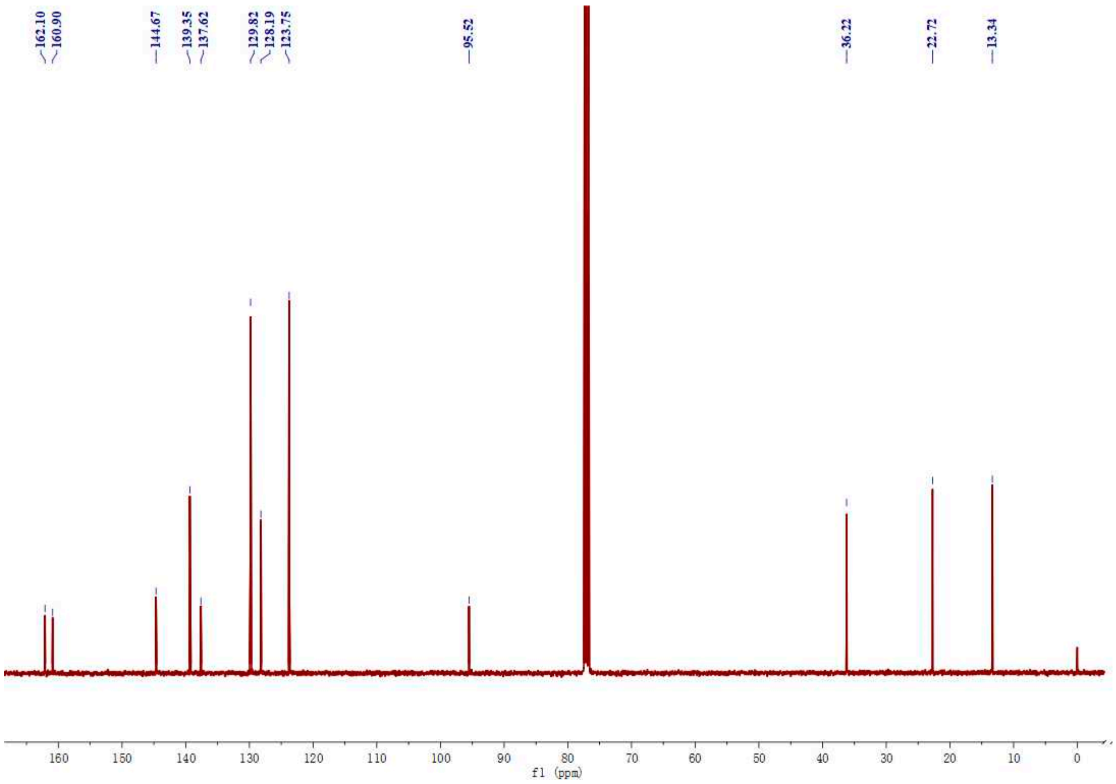
162.10 160.90 144.67 139.35 137.62 129.82 128.19 123.75 95.52 36.22 22.72 13.34
160 150 140 130 120 110 100 90 80 70 60 50 40 30 20 10 0
f1 (ppm)

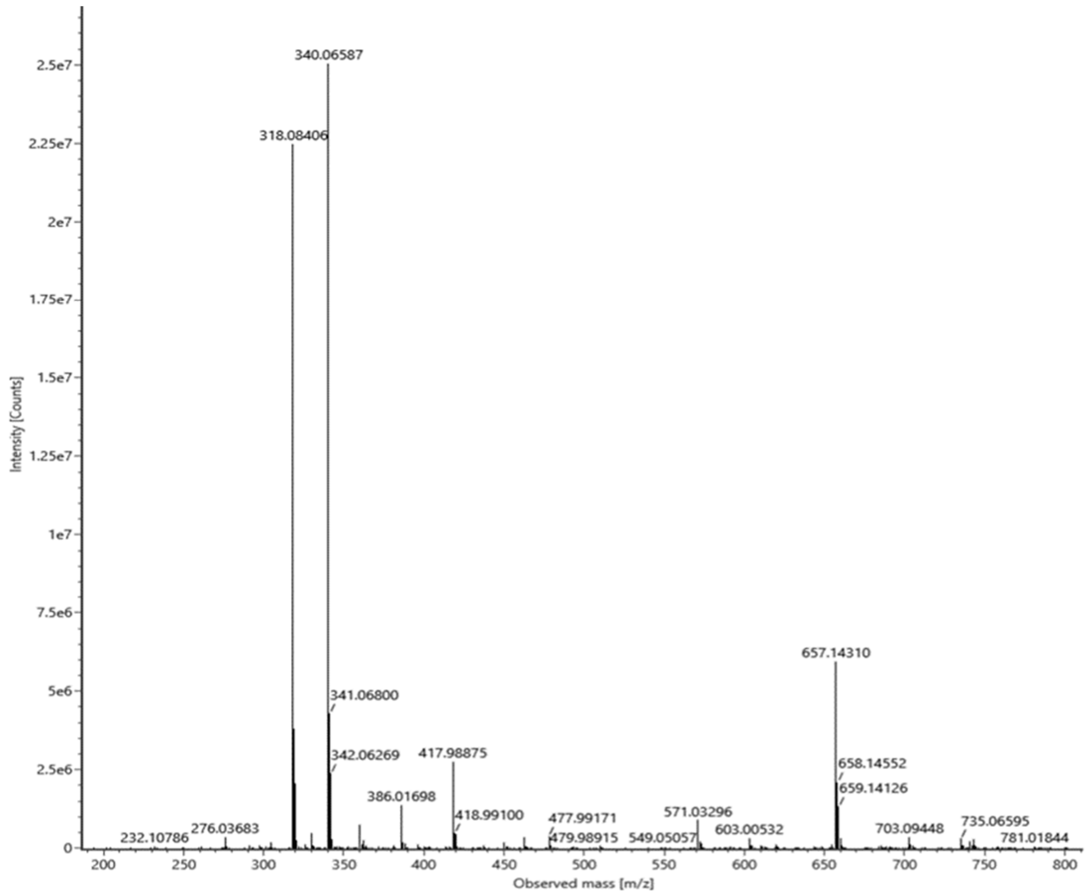

Figure S28. ^{1}H NMR, ^{13}C NMR and HRMS for title compound **E$_{23}$**.

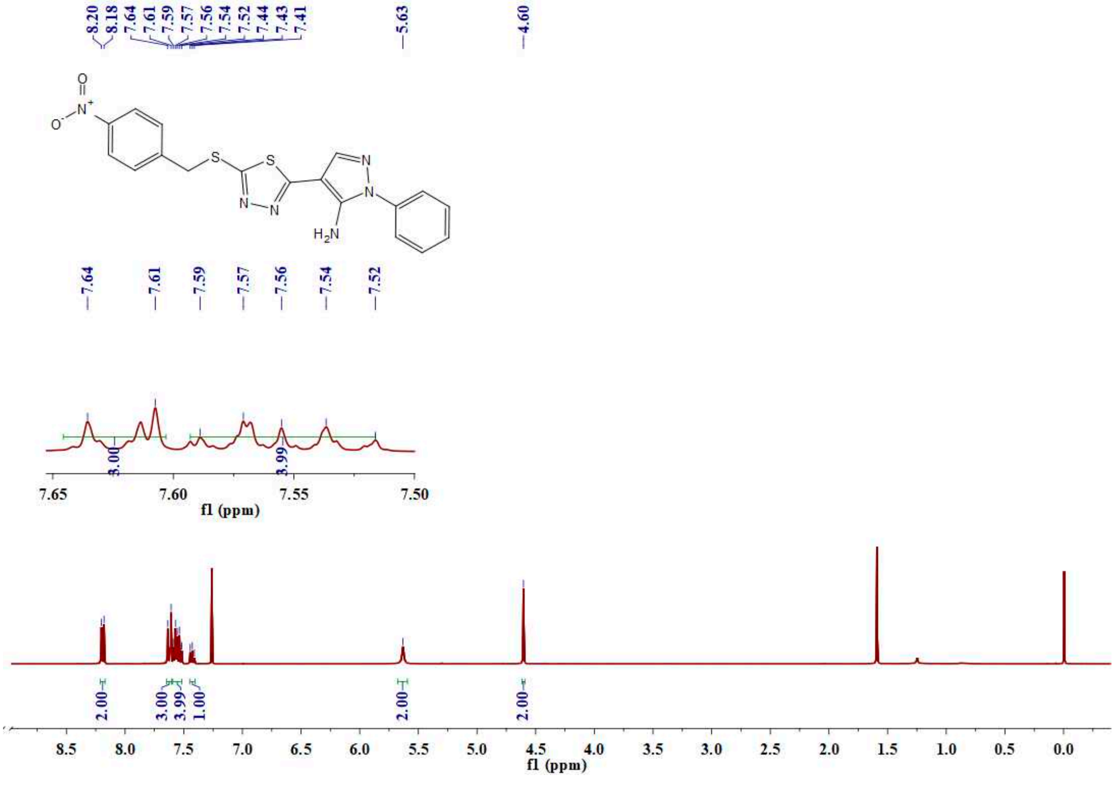

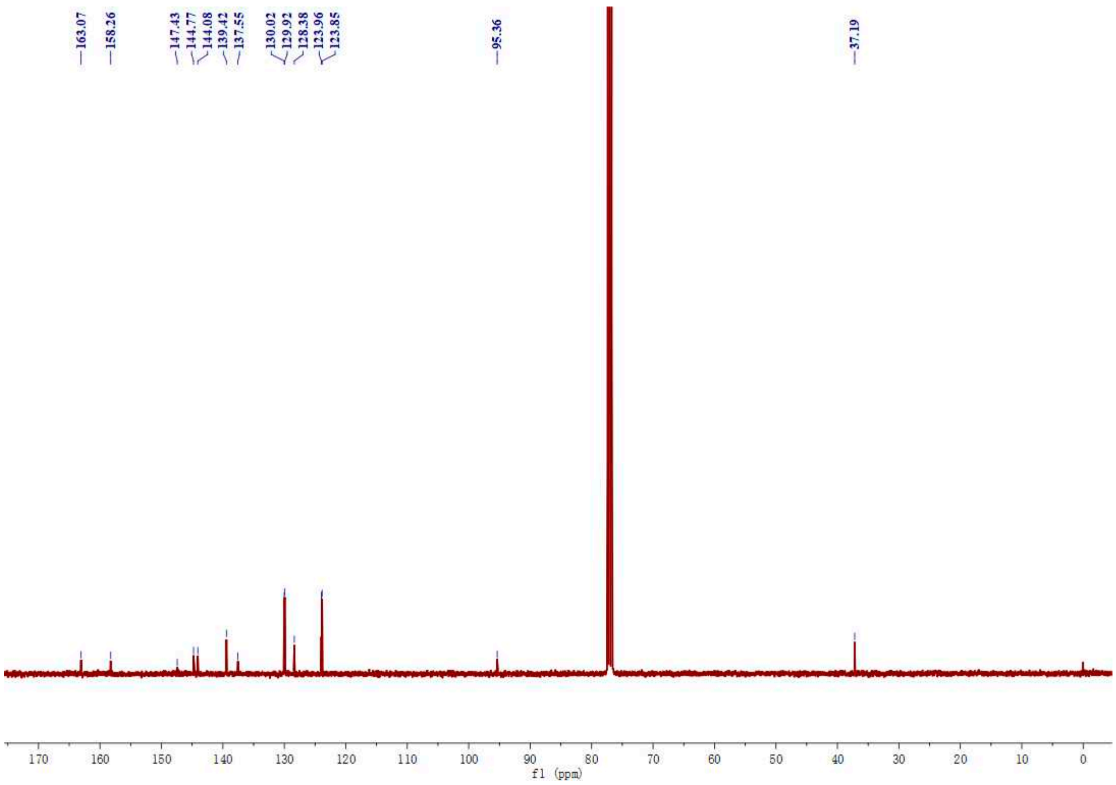

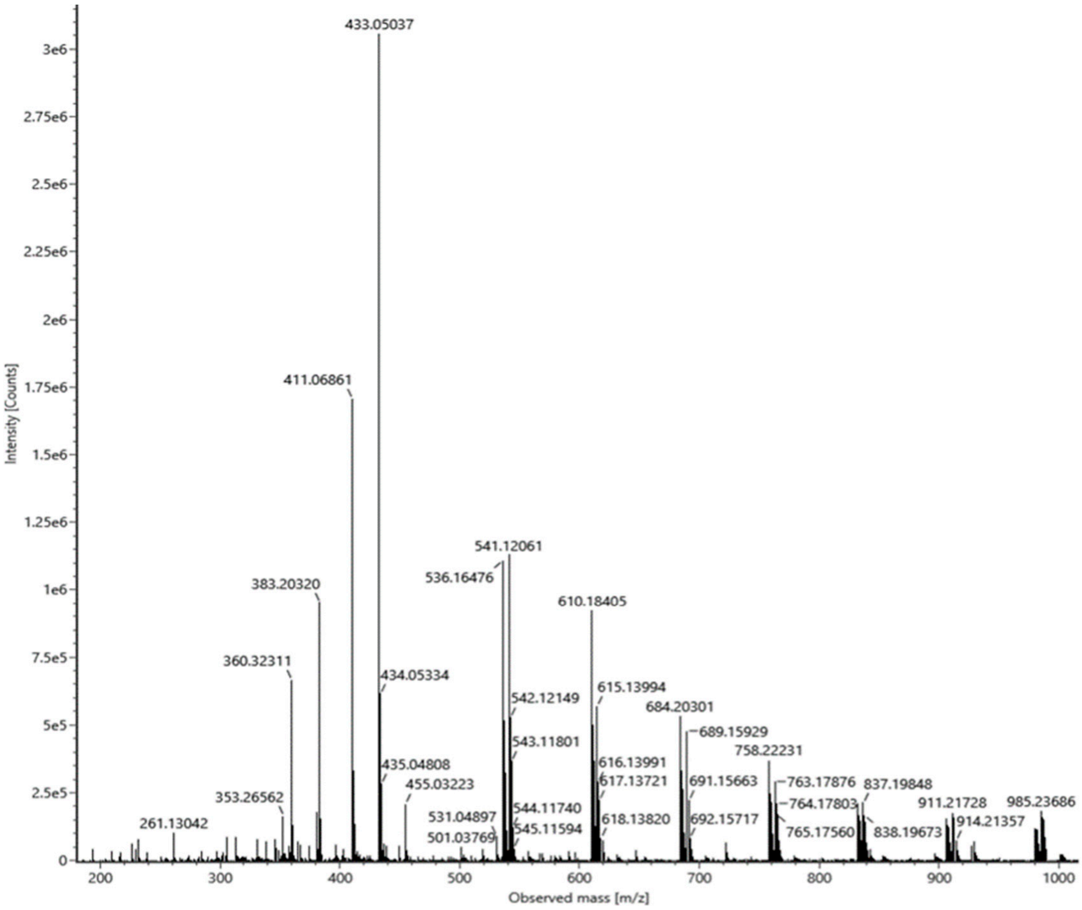

Figure S29. ^{1}H NMR, ^{13}C NMR and HRMS for title compound **E$_{24}$**.

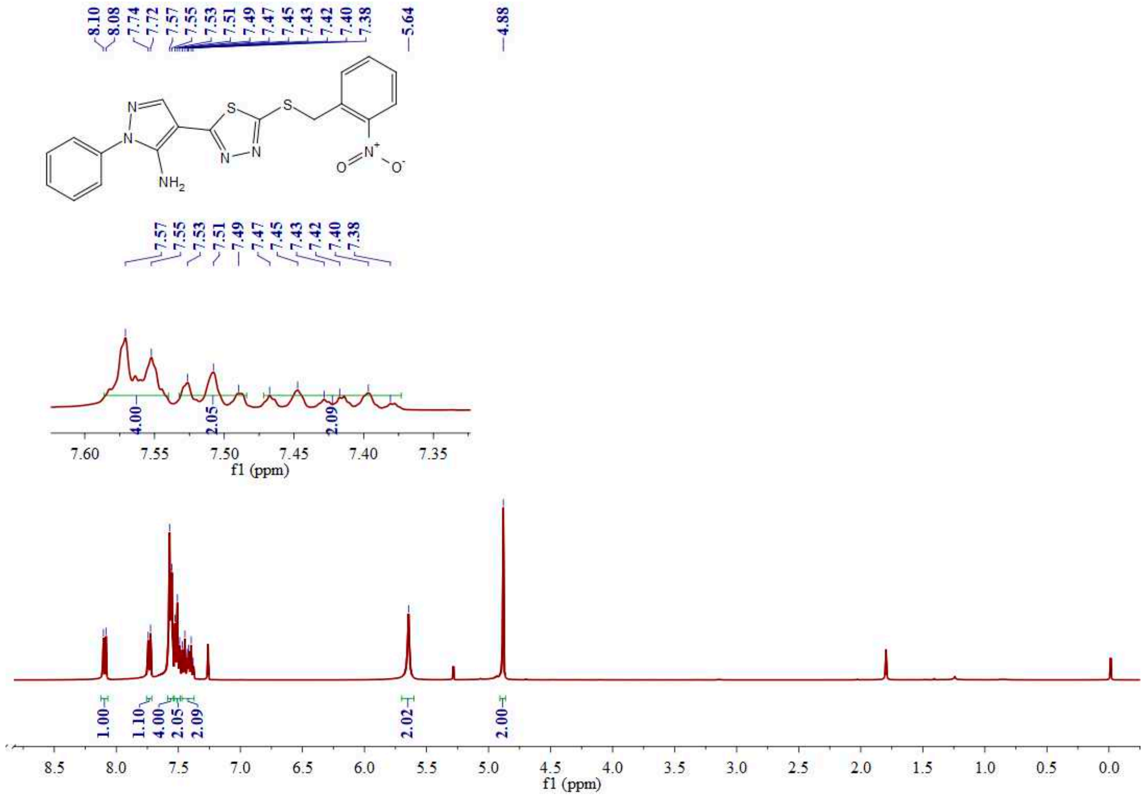
8.10
8.08
7.74
7.72
7.57
7.55
7.53
7.51
7.49
7.47
7.45
7.43
7.42
7.40
7.38
5.64
4.88
N
N
S
N
N
S
NH2
O
N+
O-
4.00
2.05
2.09
7.60
7.55
7.50
7.45
7.40
7.35
f1 (ppm)
1.00
1.10
4.00
2.05
2.09
2.02
2.00
8.5
8.0
7.5
7.0
6.5
6.0
5.5
5.0
4.5
4.0
3.5
3.0
2.5
2.0
1.5
1.0
0.5
0.0
f1 (ppm)

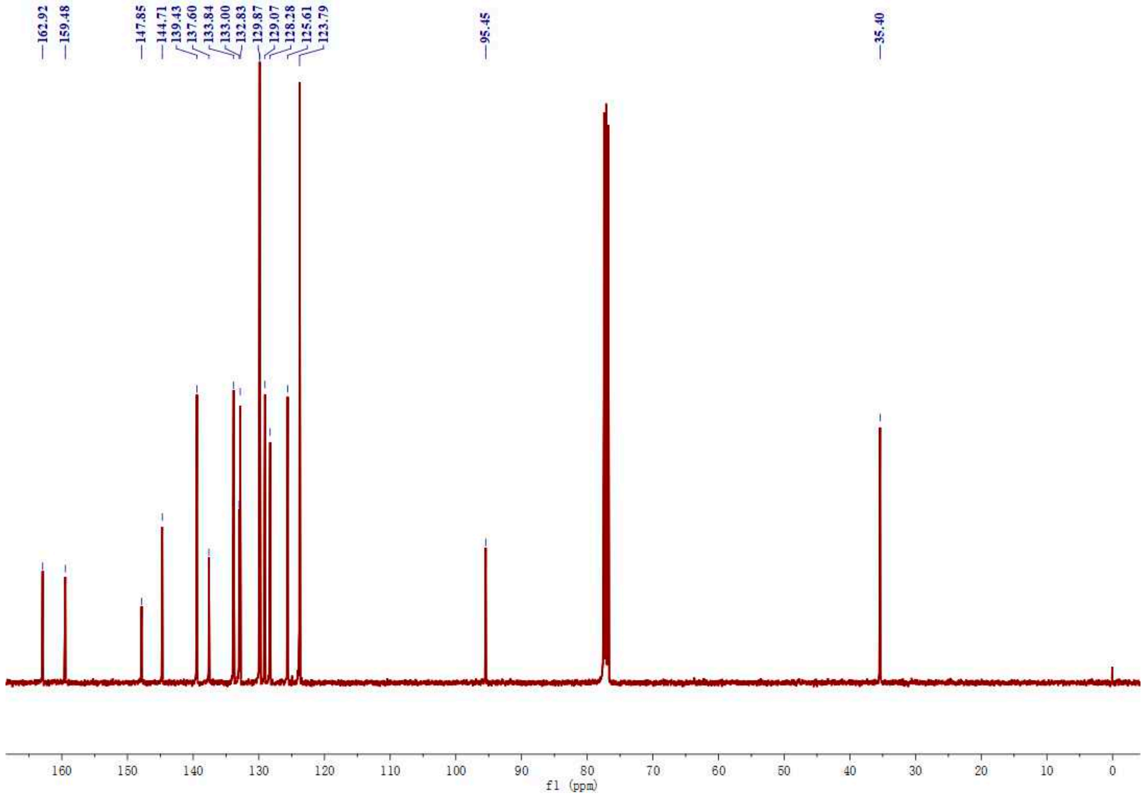
162.92
159.48
147.85
144.71
139.43
137.60
133.84
133.00
132.83
129.87
129.07
128.28
125.61
123.79
95.45
35.40
160
150
140
130
120
110
100
90
80
70
60
50
40
30
20
10
0
f1 (ppm)

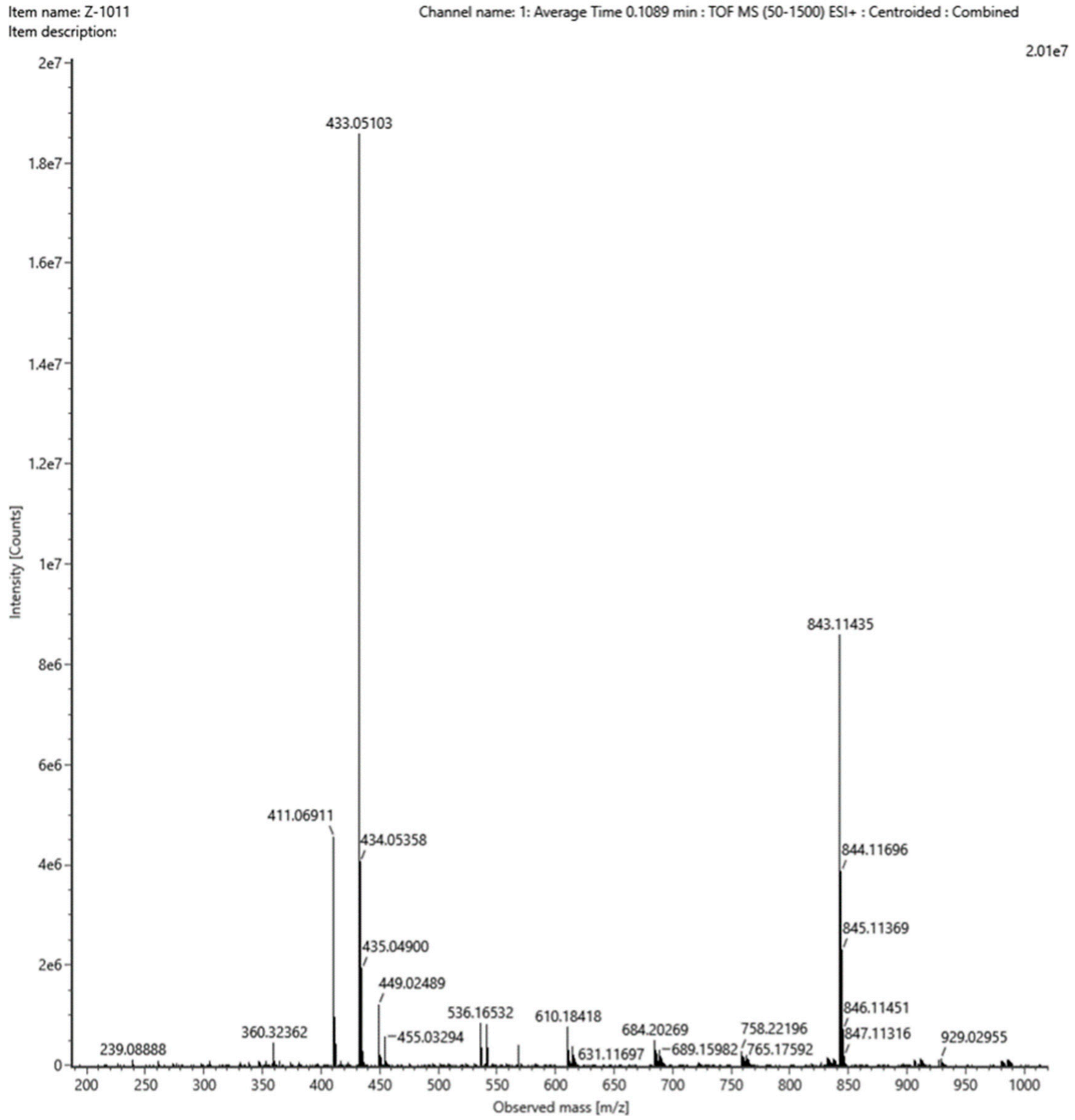

Figure S30. ^{1}H NMR, ^{13}C NMR and HRMS for title compound $\mathbf{E_{25}}$.

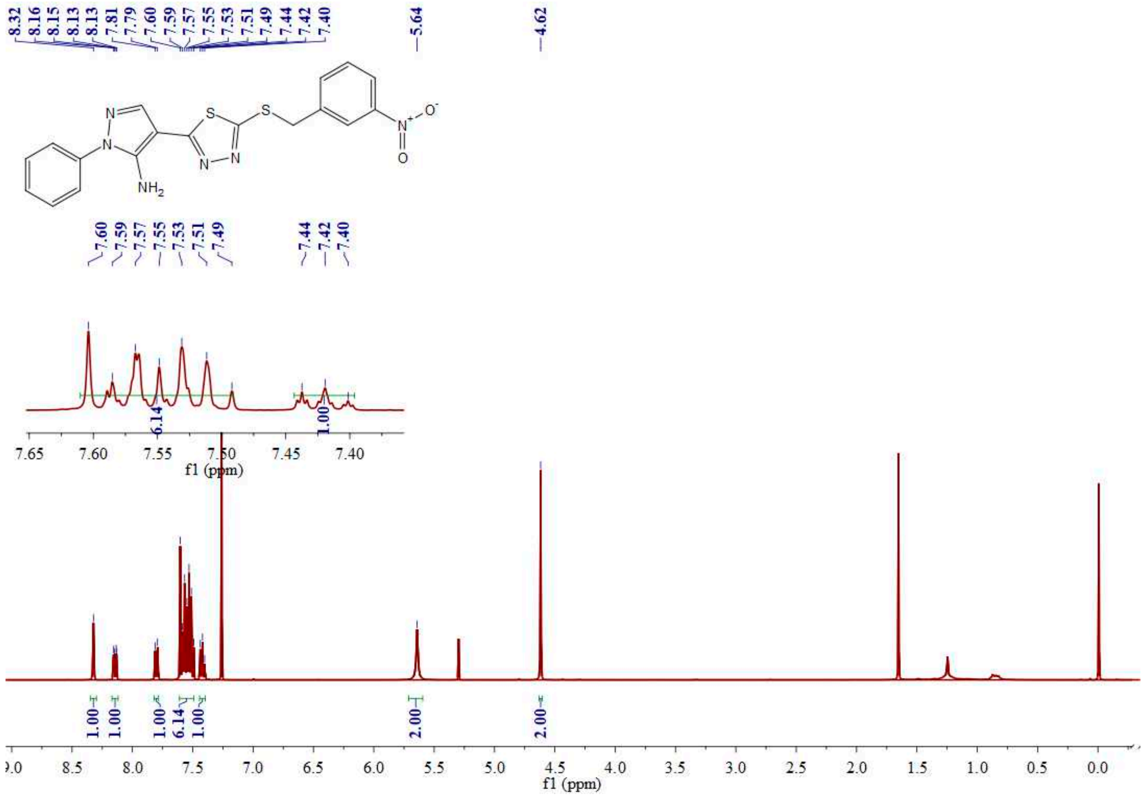
8.32
8.16
8.15
8.13
8.13
7.81
7.79
7.60
7.59
7.57
7.55
7.53
7.51
7.49
7.44
7.42
7.40
5.64
4.62
S
S
N
N
N
N
NH2
N+
O-
O
1.00
1.00
1.00
6.14
1.00
2.00
2.00
f1 (ppm)

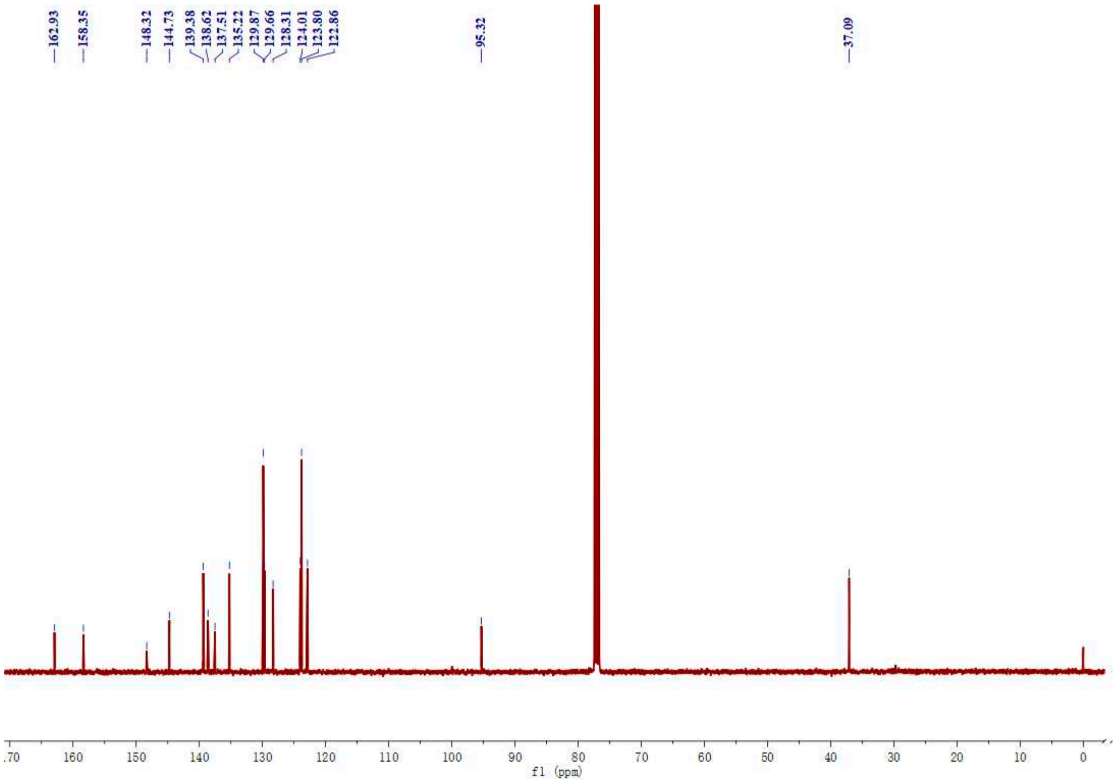
162.93
158.35
148.32
144.73
139.38
138.62
137.51
135.22
129.87
129.66
128.31
124.01
123.80
122.86
95.32
37.09
f1 (ppm)

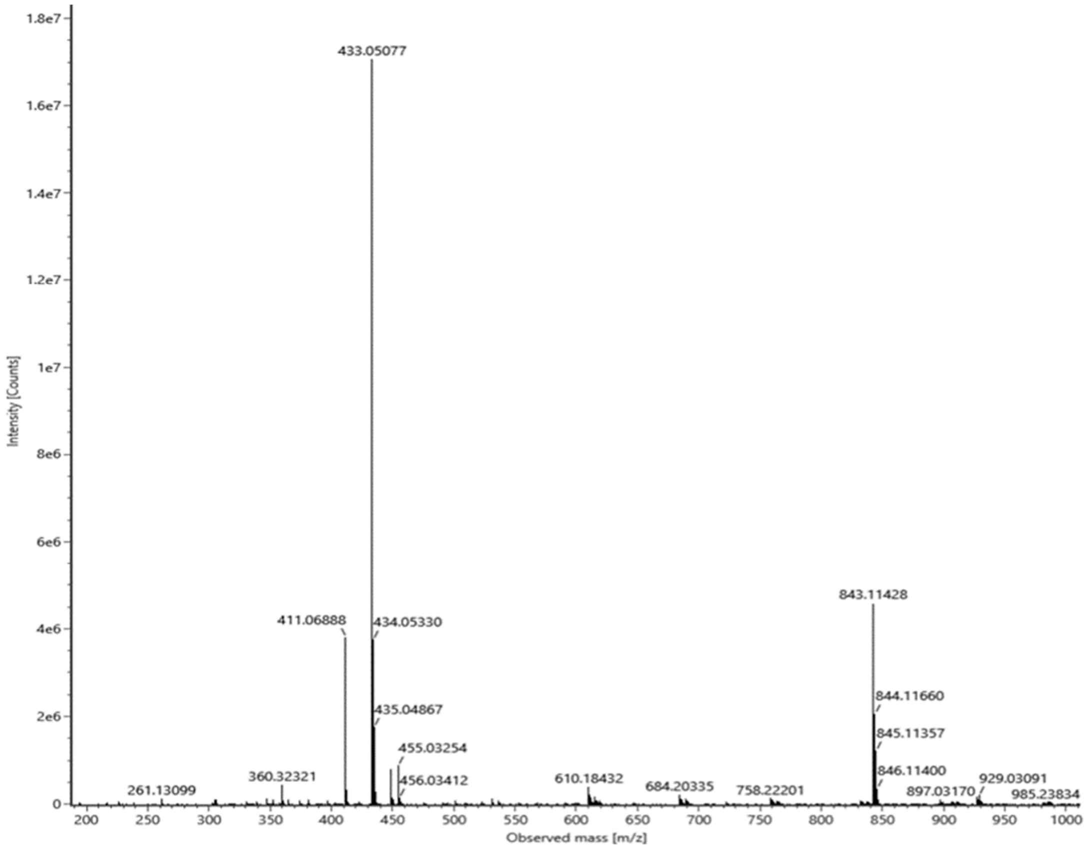

Figure S31. ^{1}H NMR, ^{13}C NMR and HRMS for title compound **E_{26}**.

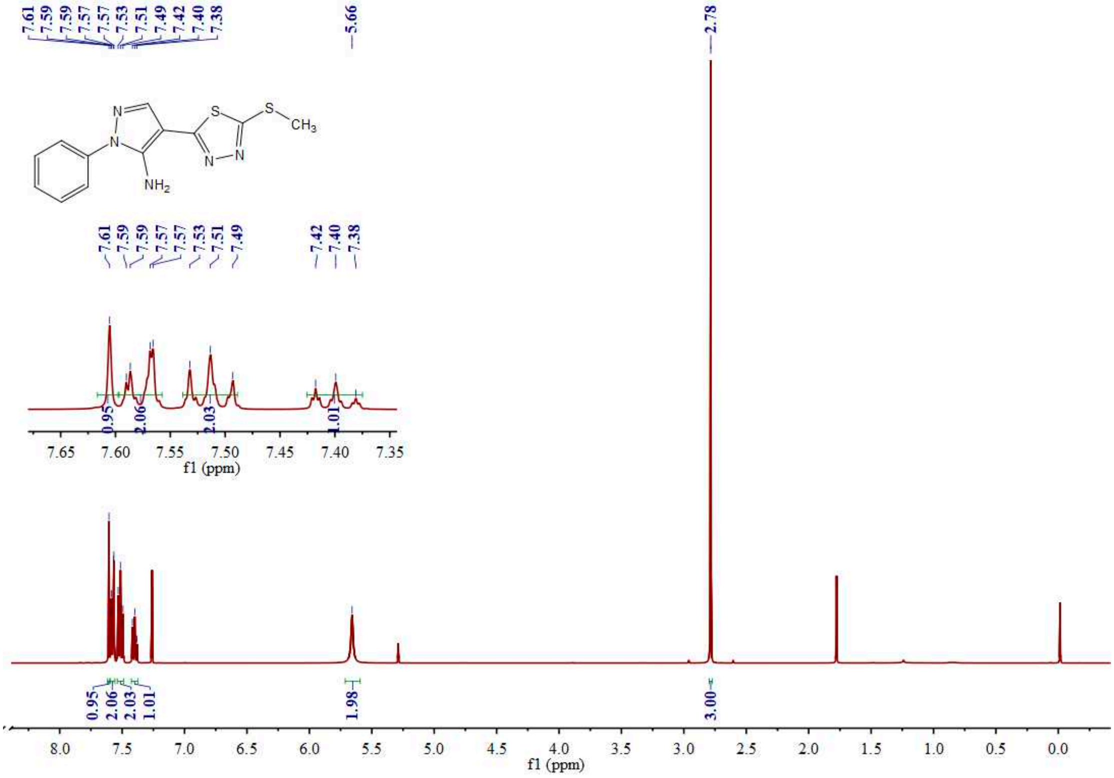

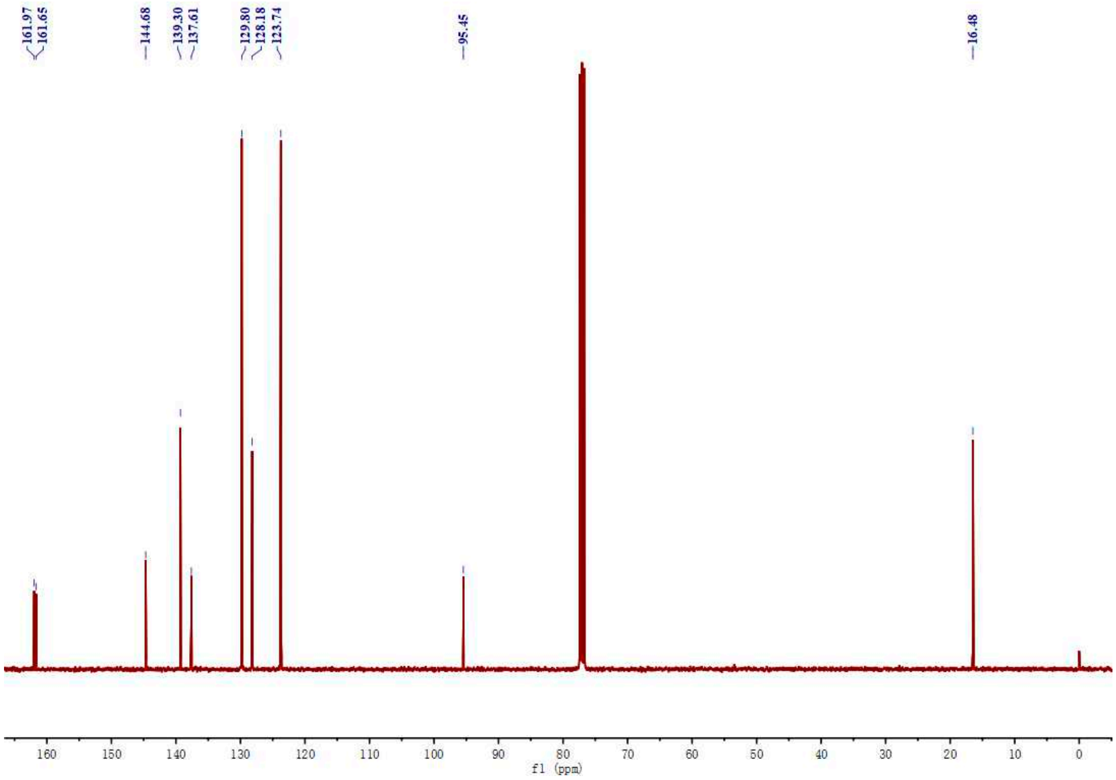

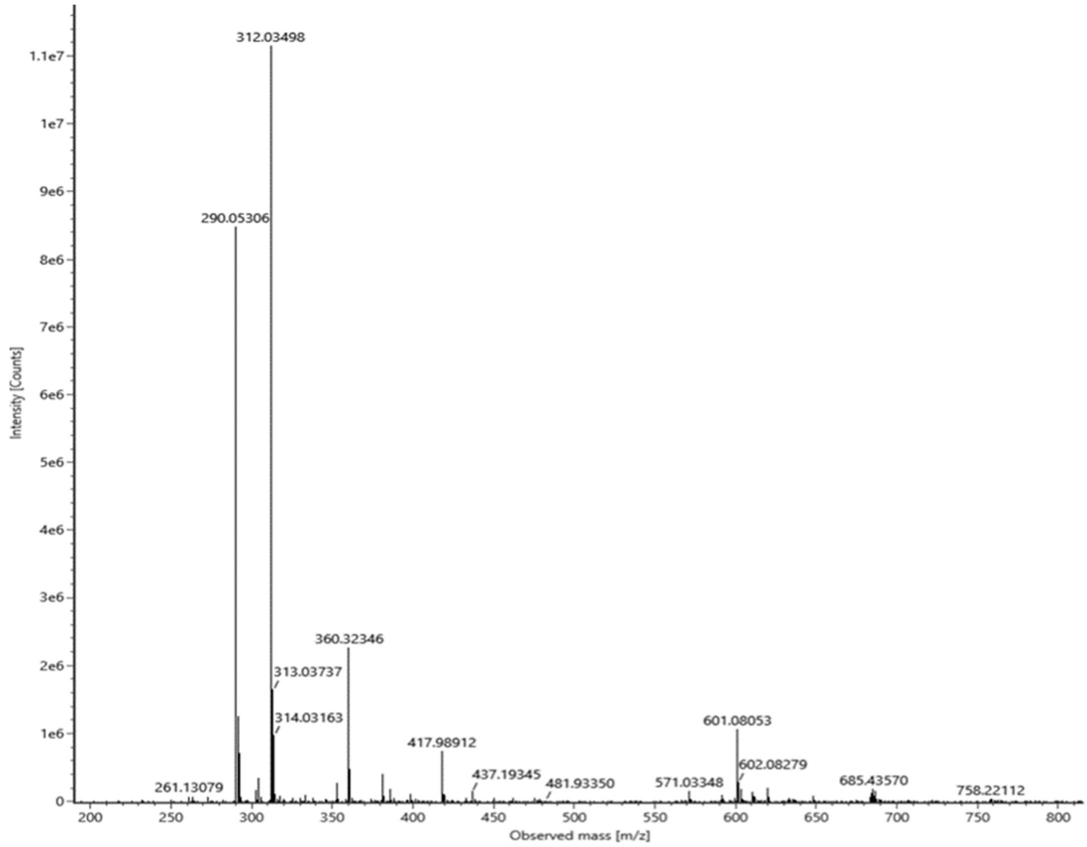

Figure S32. ^{1}H NMR, ^{13}C NMR and HRMS for title compound $\mathbf{E_{27}}$.

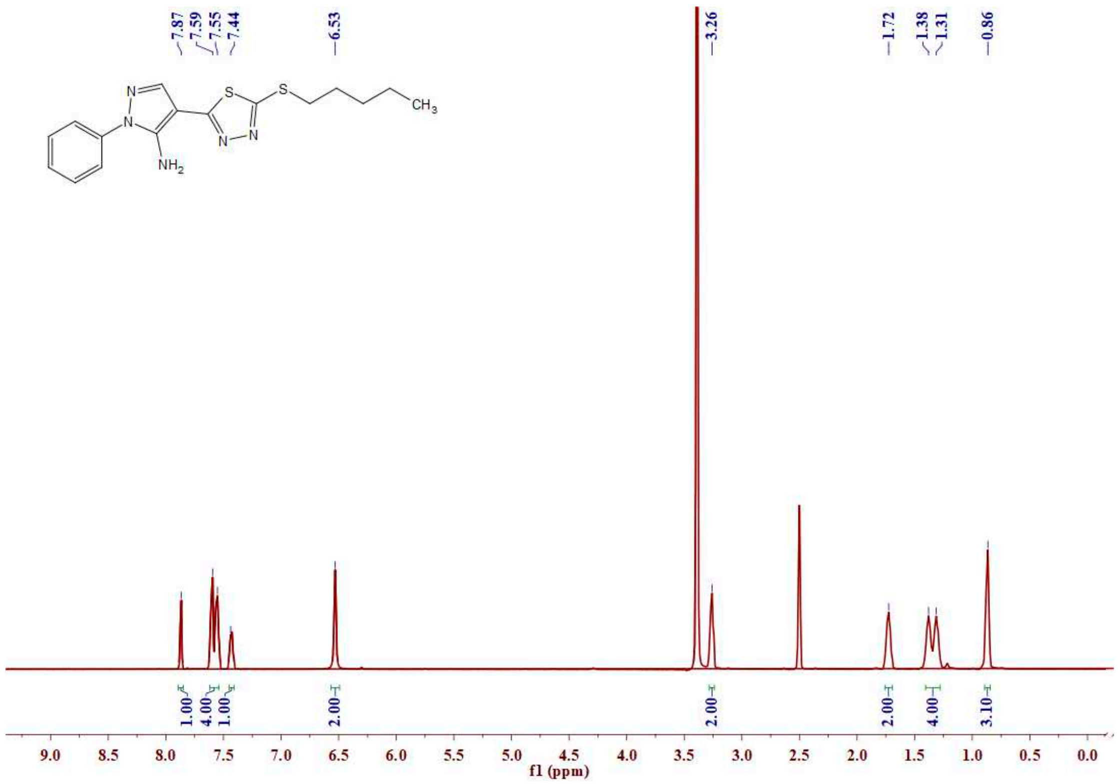
7.87
7.59
7.55
7.44
6.53
3.26
1.72
1.38
1.31
0.86
N
N
S
S
N
N
CH3
NH2
1.00
4.00
1.00
2.00
2.00
2.00
4.00
3.10
9.0 8.5 8.0 7.5 7.0 6.5 6.0 5.5 5.0 4.5 4.0 3.5 3.0 2.5 2.0 1.5 1.0 0.5 0.0
f1 (ppm)

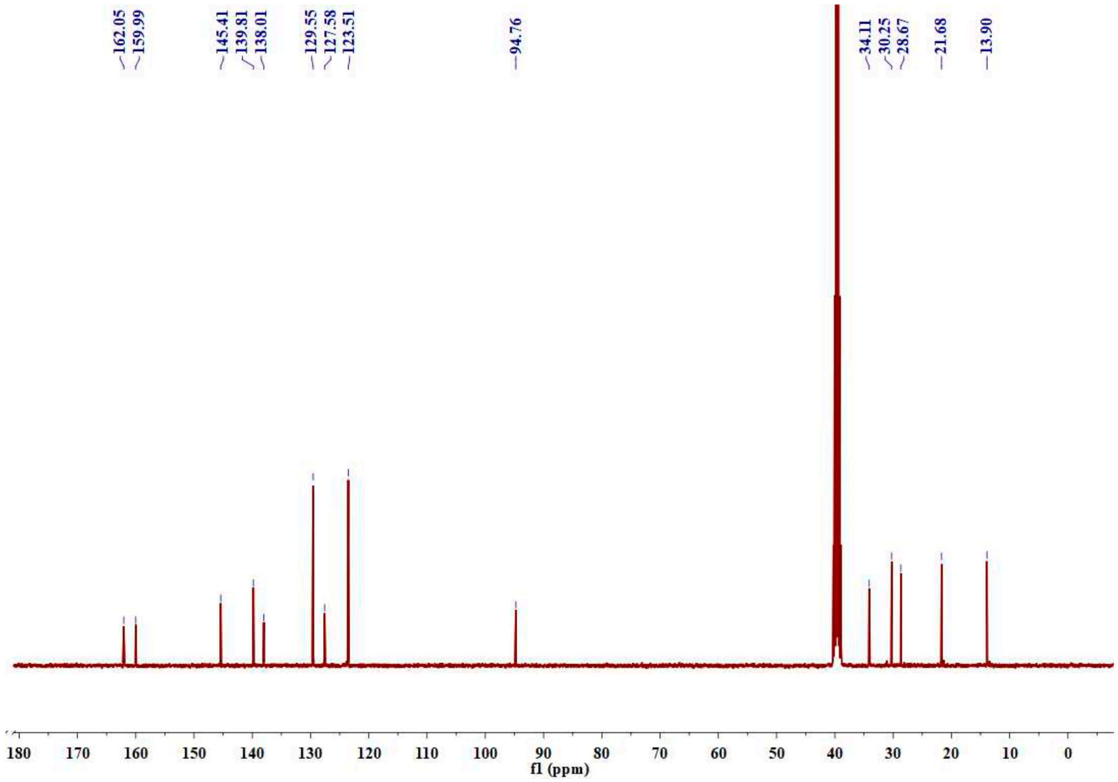
162.05
159.99
145.41
139.81
138.01
129.55
127.58
123.51
94.76
34.11
30.25
28.67
21.68
13.90
180 170 160 150 140 130 120 110 100 90 80 70 60 50 40 30 20 10 0
f1 (ppm)

92 #55 RT: 0.55 AV: 1 NL: 3.39E+008
T: FTMS + p ESI Full ms [150.0000-2200.0000]

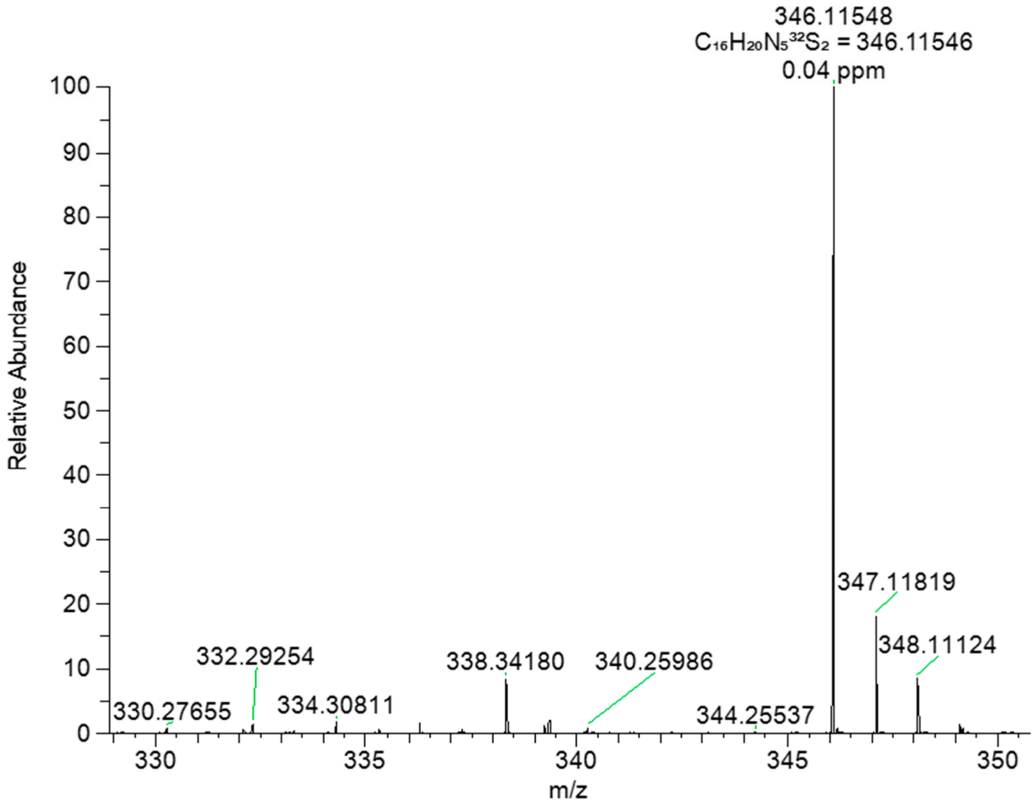

Figure S33. ^{1}H NMR, ^{13}C NMR and HRMS for title compound **E28**.
